# Supplementary material for: Genome-wide analyses of non-syndromic cleft lip with palate identify 14 novel loci and genetic heterogeneity
Source: Nat Commun. 2017 Feb 24;8:14364. doi: 10.1038/ncomms14364 (PMC5333091; doi:10.1038/ncomms14364)
Supplement: Supplementary Information — Supplementary Figures and Supplementary Tables [file ncomms14364-s1.pdf]

Supplementary Figures:

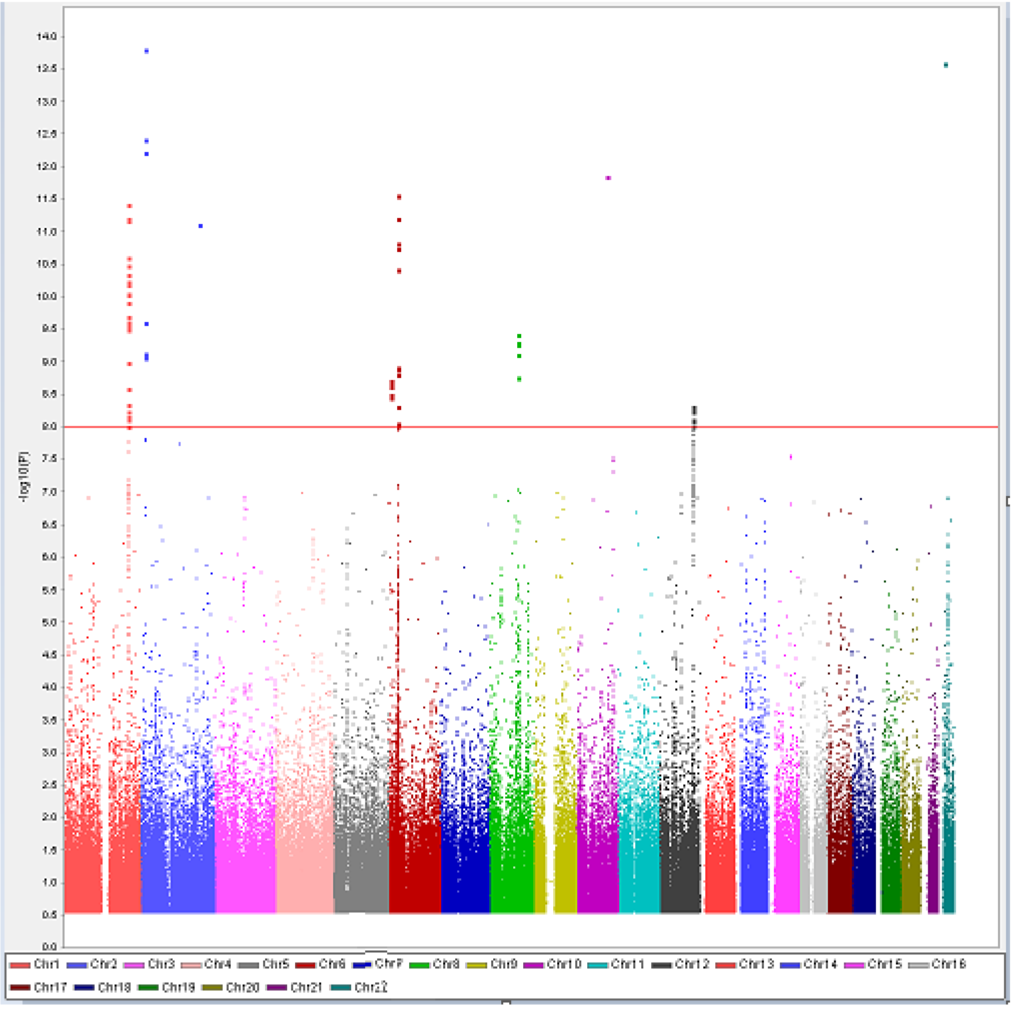

Supplementary Figure 1| Manhattan plot of the association evidence in discovery GWAS stage. The genome-wide *P* values from the Cochran-Armitage trend test on 803,202 SNPs in 2,033 NSCLP cases and 4,051 controls of Chinese Han ancestry are presented. The chromosomal distribution of all *P* values (as $-\log_{10} P$ values) is shown for each chromosome depicted in a different color. The red horizontal line presents a *P* value threshold of 10^{-8}.

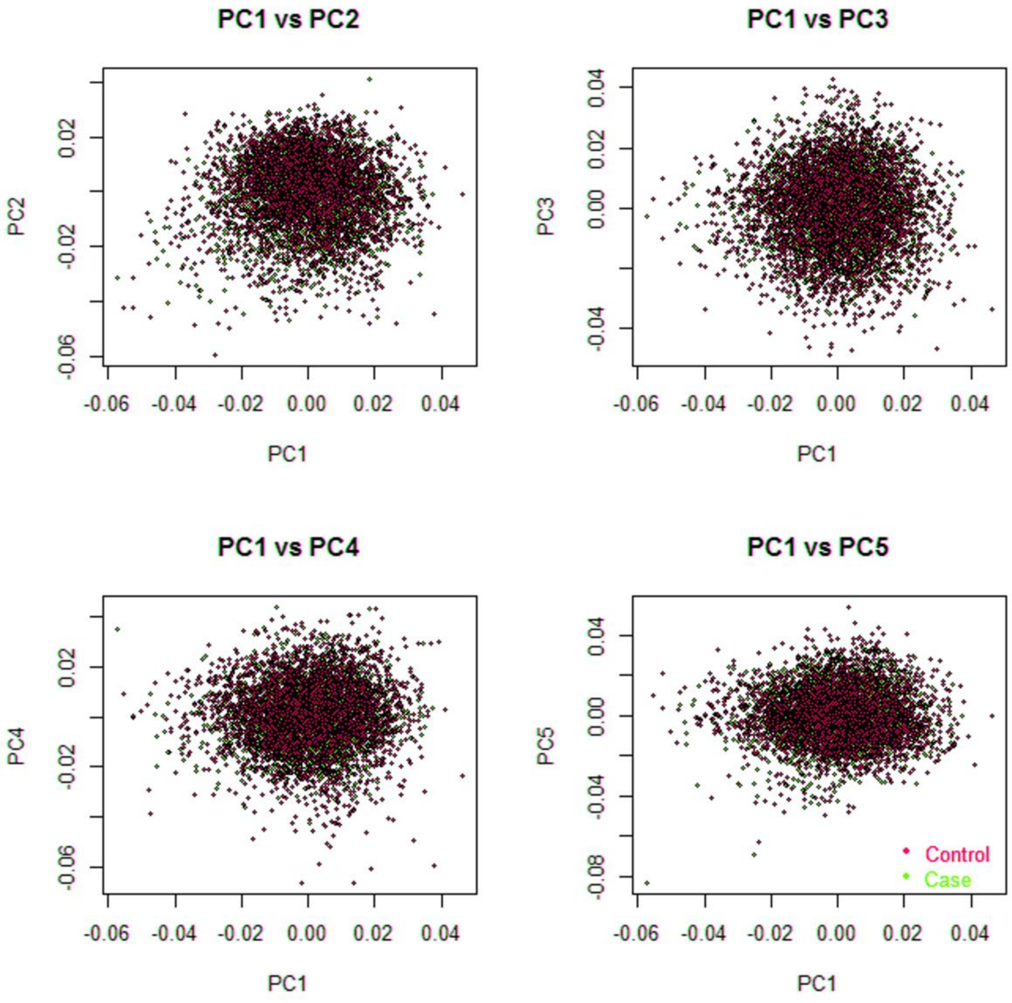

Supplementary Figure 2| The principal components analyses (PCA) were performed in 6,147 unrelated samples (2,096 NSCLP cases and 4,051 controls) in the discovery GWAS stage.

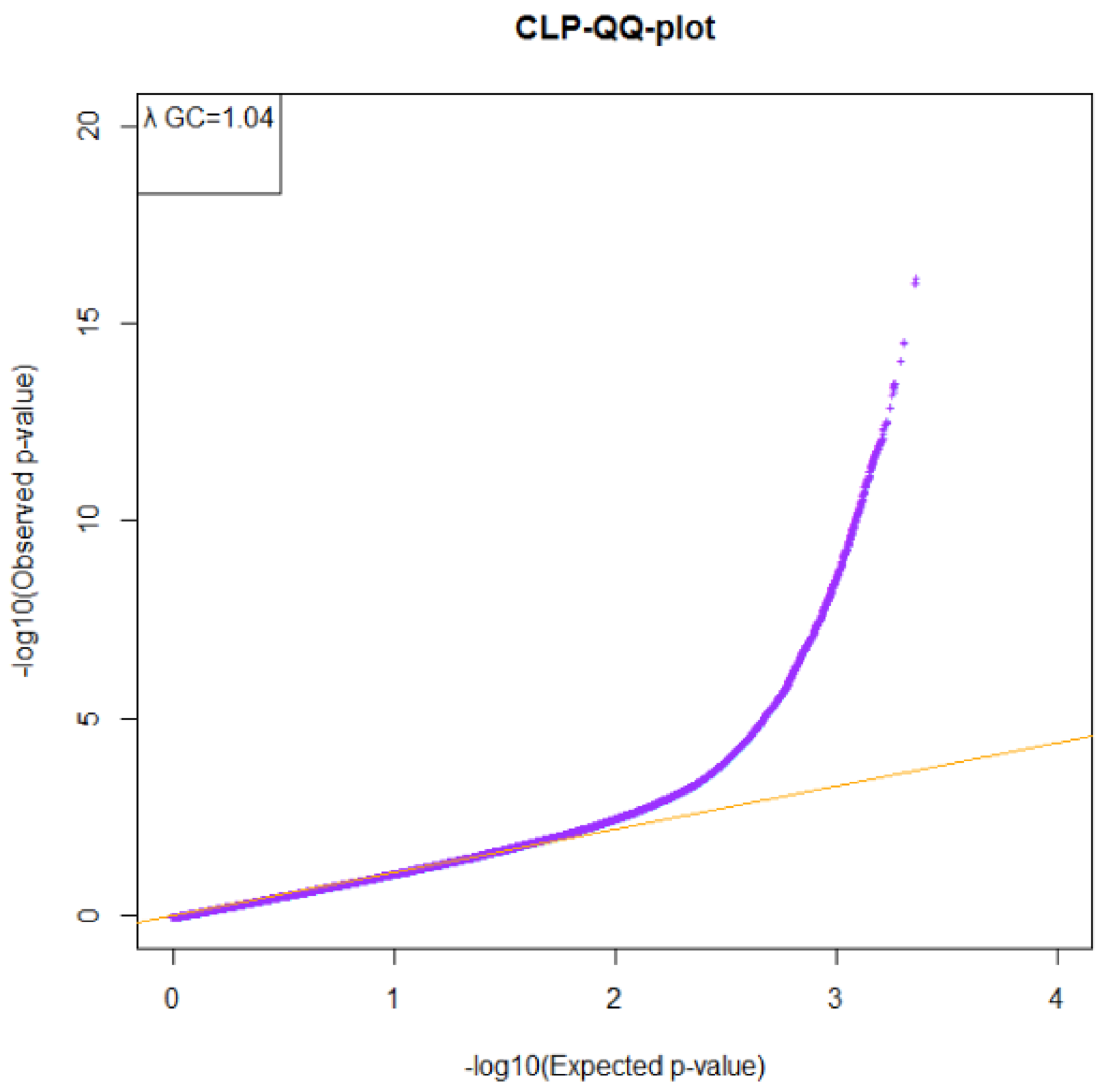

SupplementaryFigure3| Quantile-quantile (Q-Q) plots for the observed *P* values (using logistic regression test) from discovery GWAS analysis in 2,033 NSCLP cases and 4,051 controls versus the expected null distribution of *P* value for association. The plot in purple is for the *P* values of all SNPs. The genomic inflation factor (λ_{GC} = 1.04) shown in purple for all the SNPs passing QC.

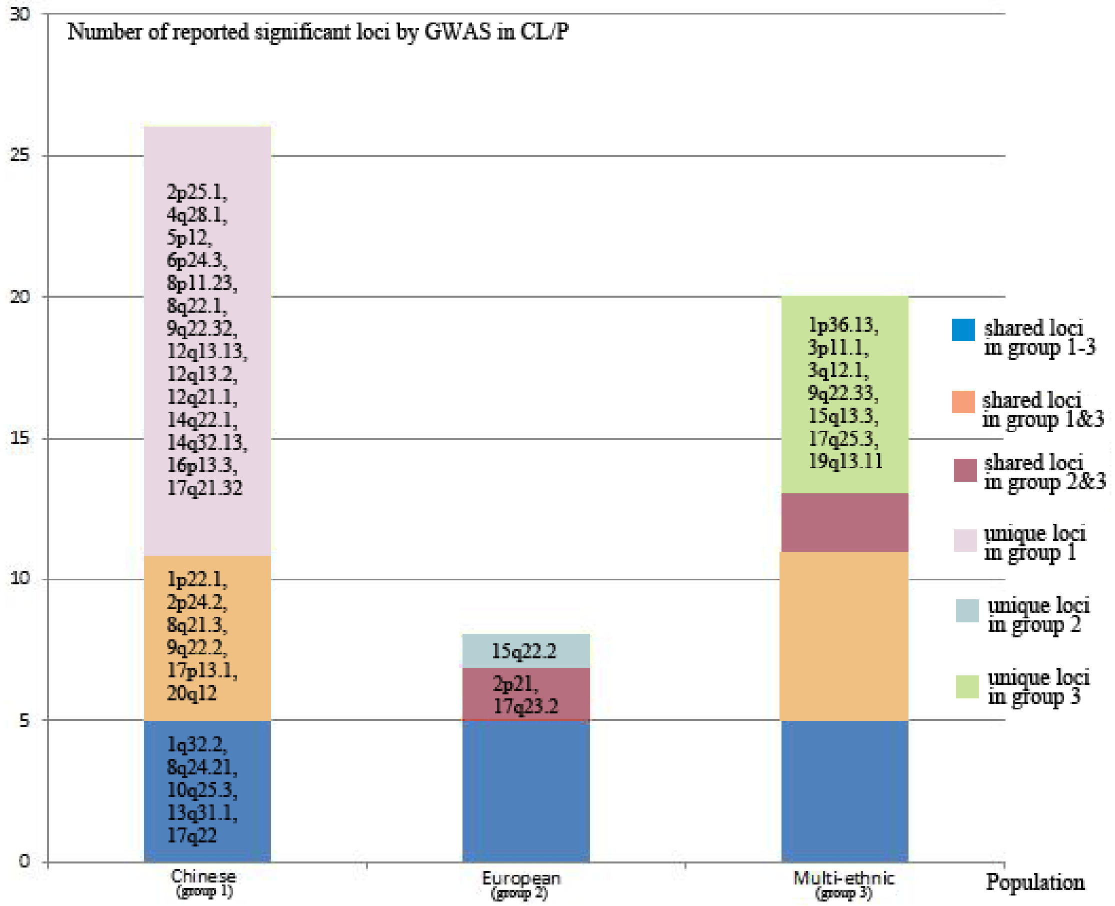

Supplementary Figure 4 | Summaryof significant associated loci with NSCL/P in different populations. Different colors represent the shared common loci or unique loci among the three groups. Multi-ethnic group include at least two races, such as European and Asian.

(a): 2p25.1 (rs287982)

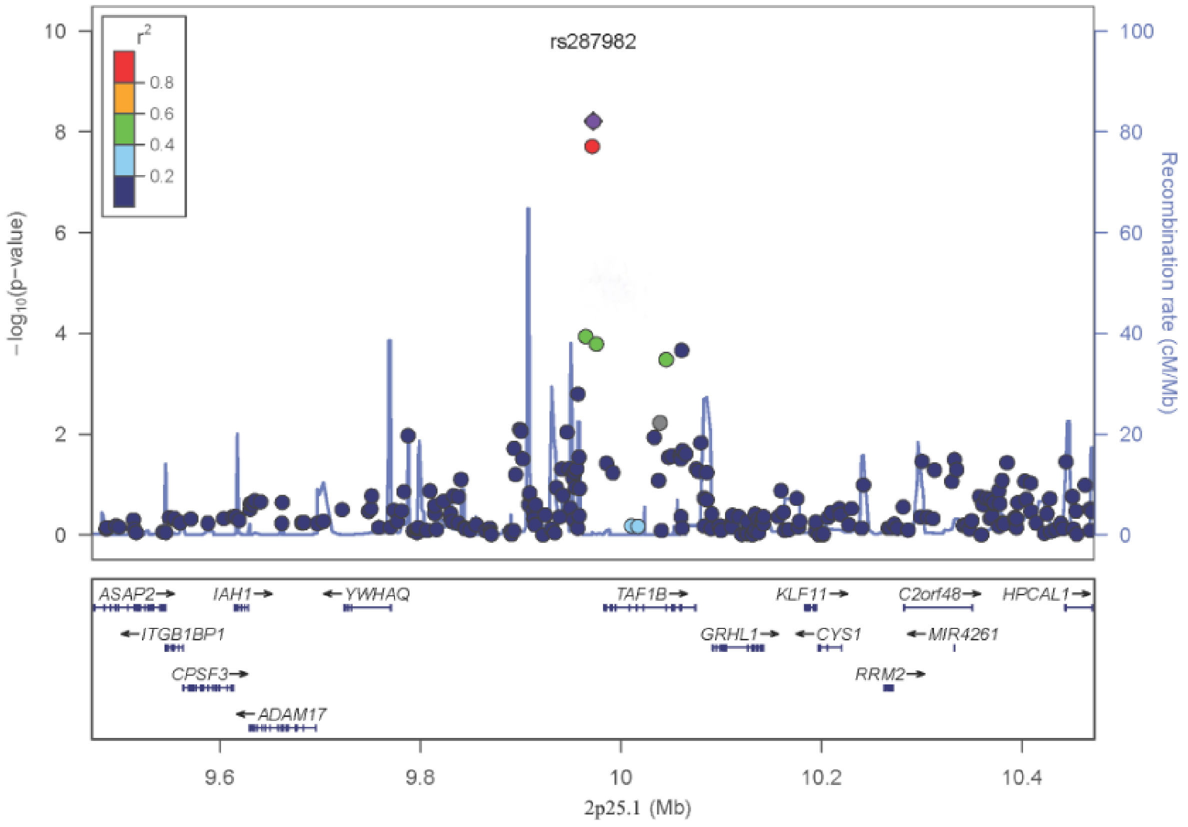

(b): 4p16.2 (rs1907989)

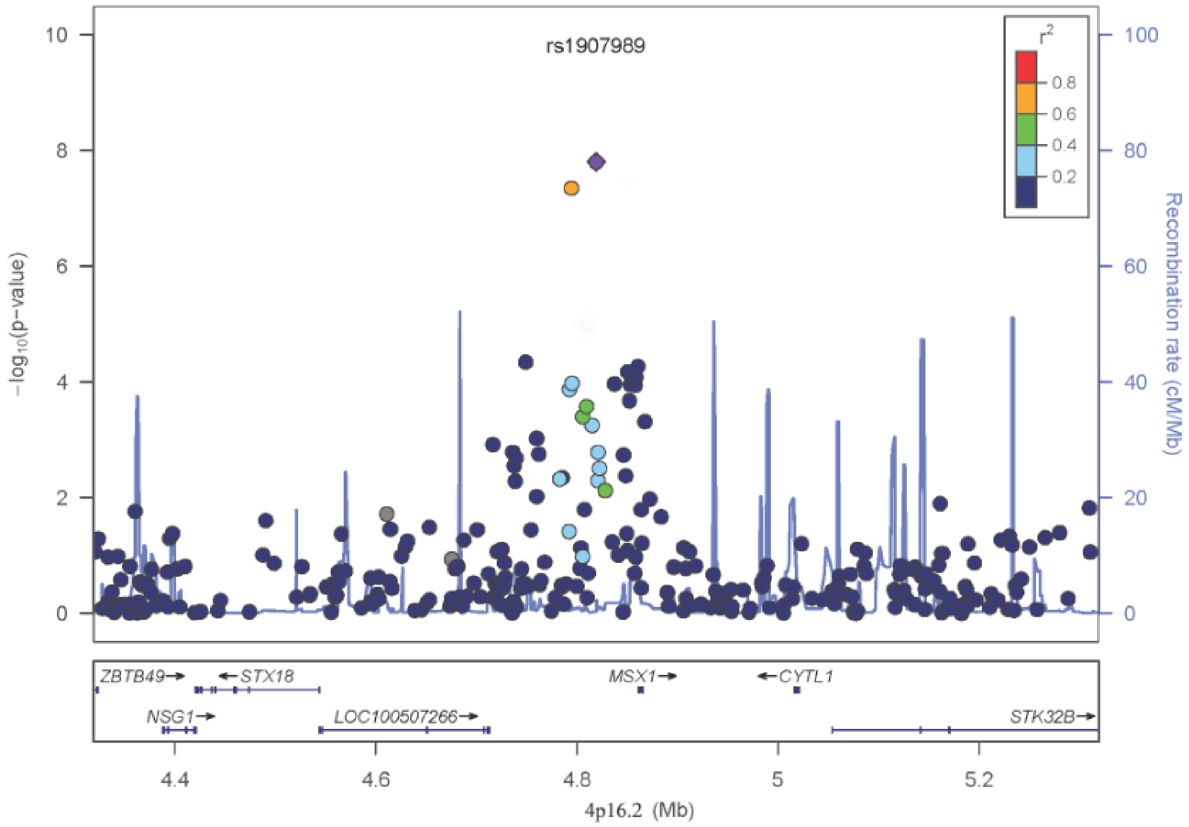

(c): 4q28.1 (rs908822)

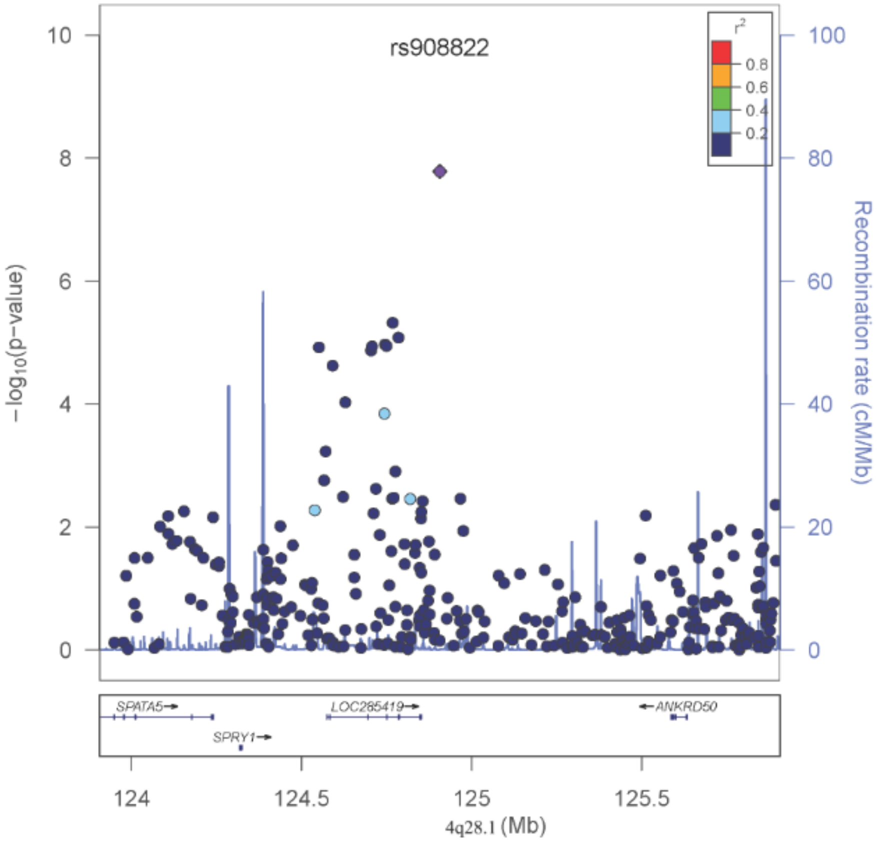

(d): 5p12 (rs10462065)

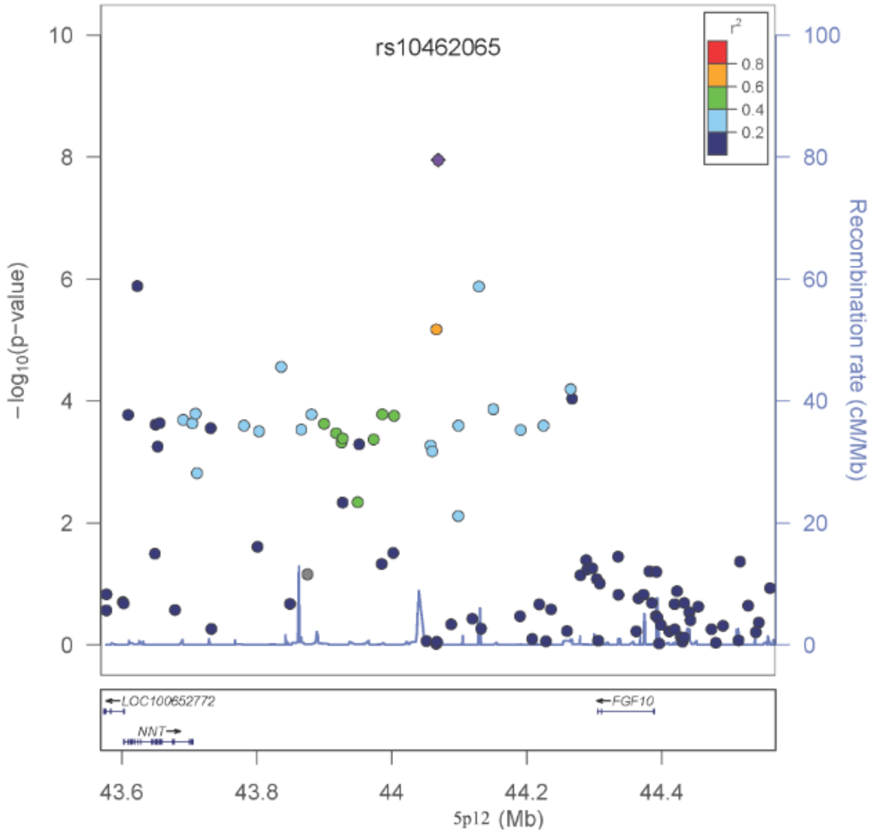

(e): 6p24.3 (rs9381107)

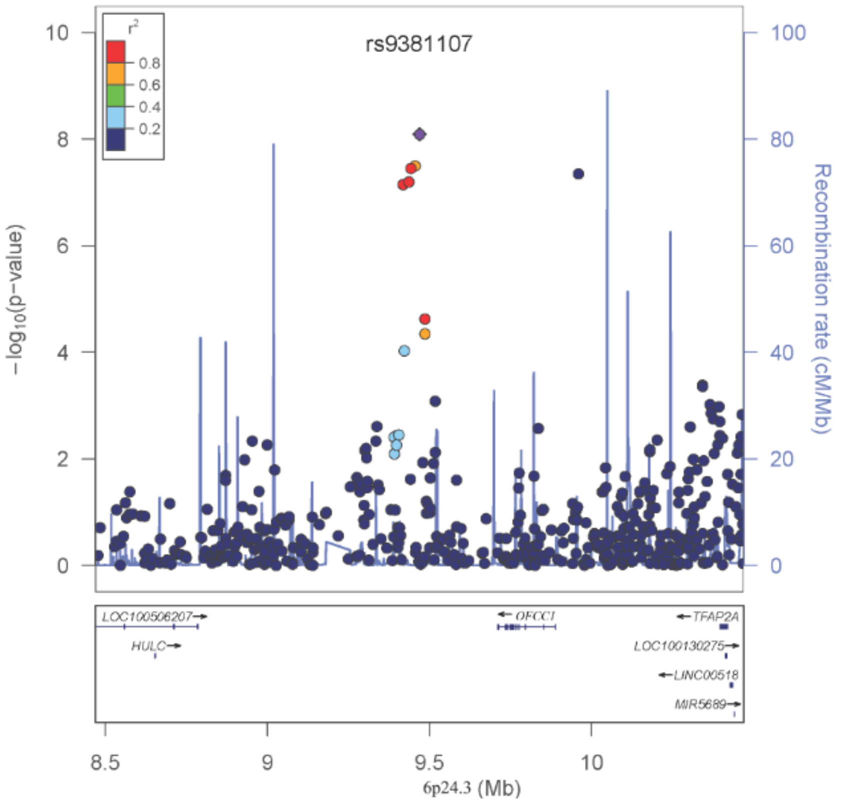

(f): 8p11.23 (rs13317)

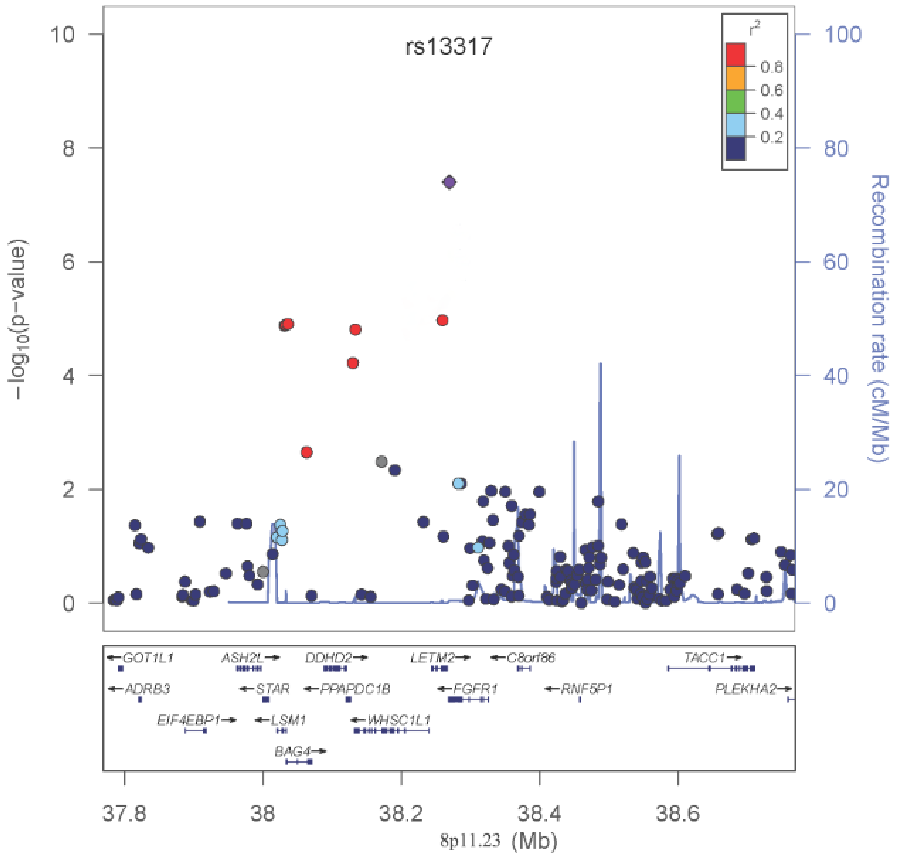

(g): 8q22.1 (rs957448)

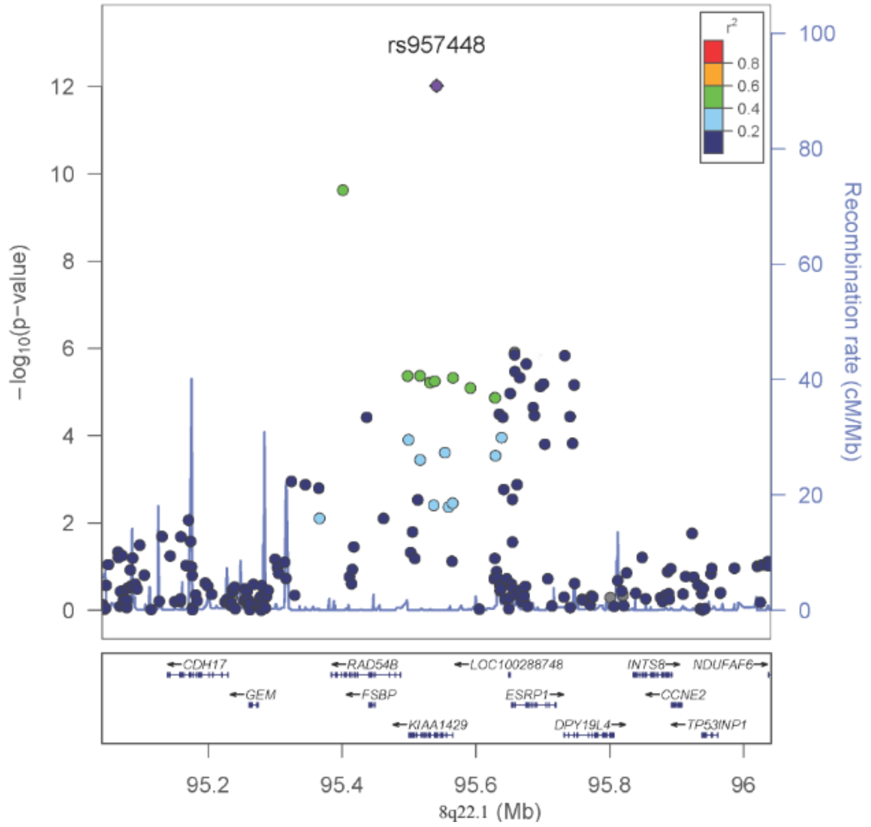

(h): 9q22.32 (rs10512248)

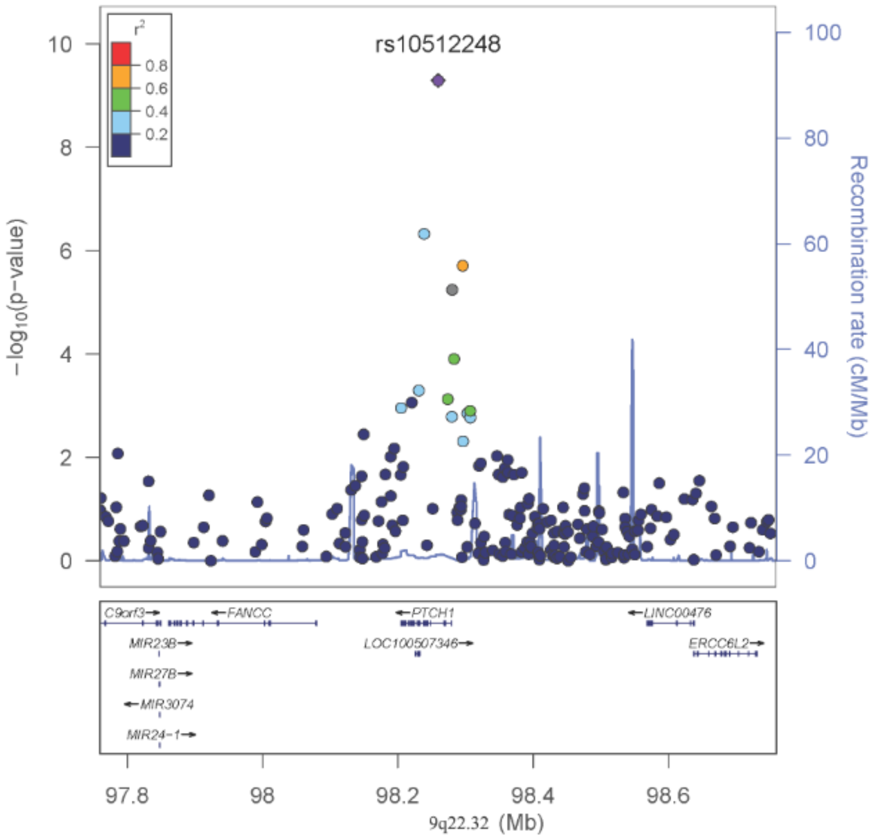

(i): 12q13.13 (rs3741442)

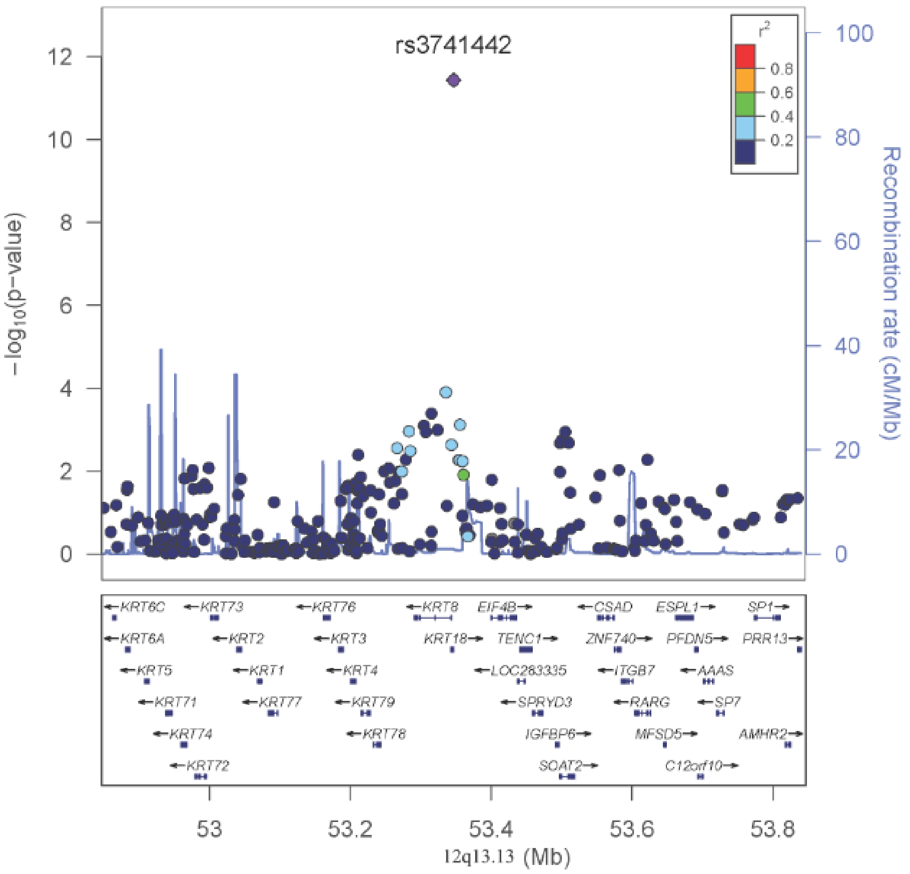

(j): 12q13.2 (rs705704)

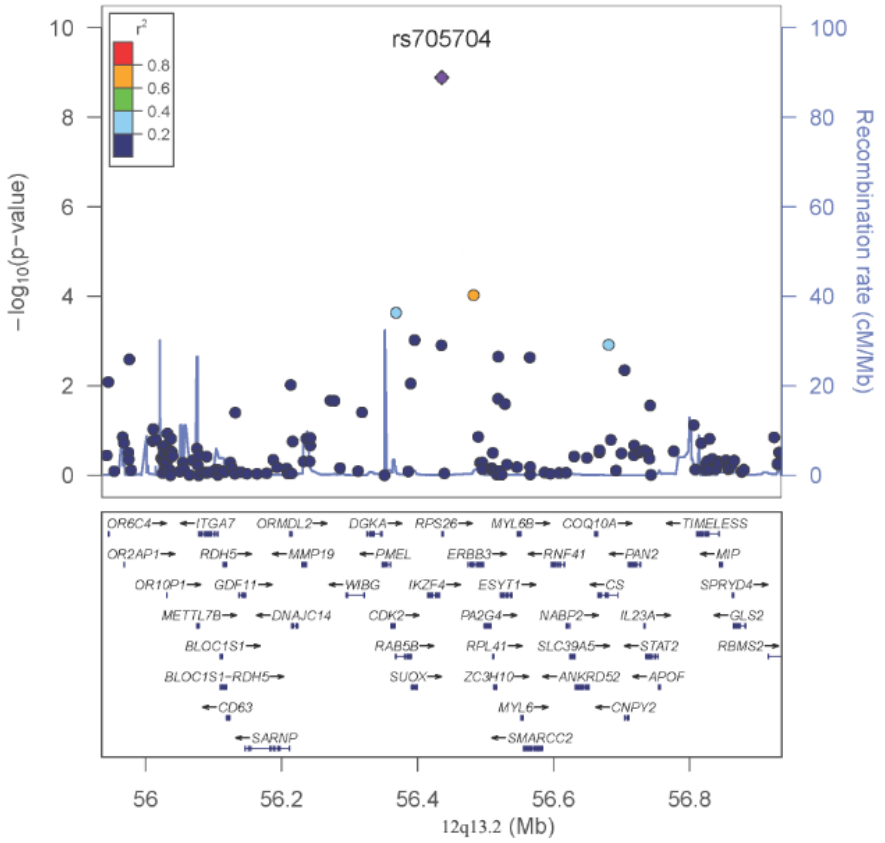

(k): 12q21.1 (rs2304269)

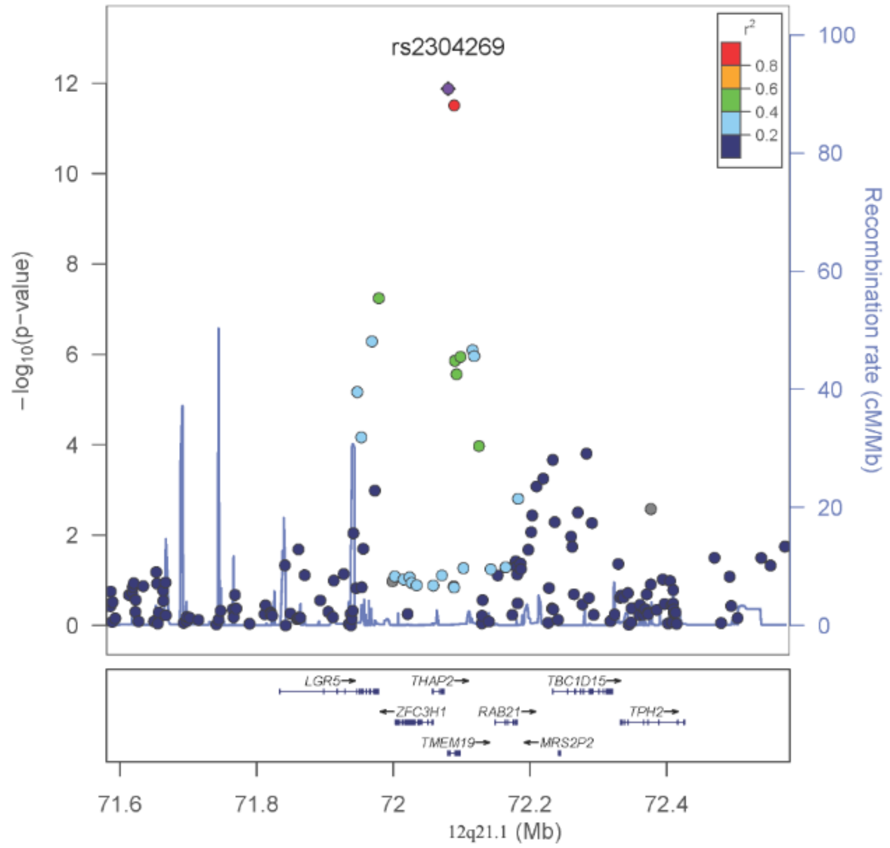

(l): 14q22.1 (rs7148069)

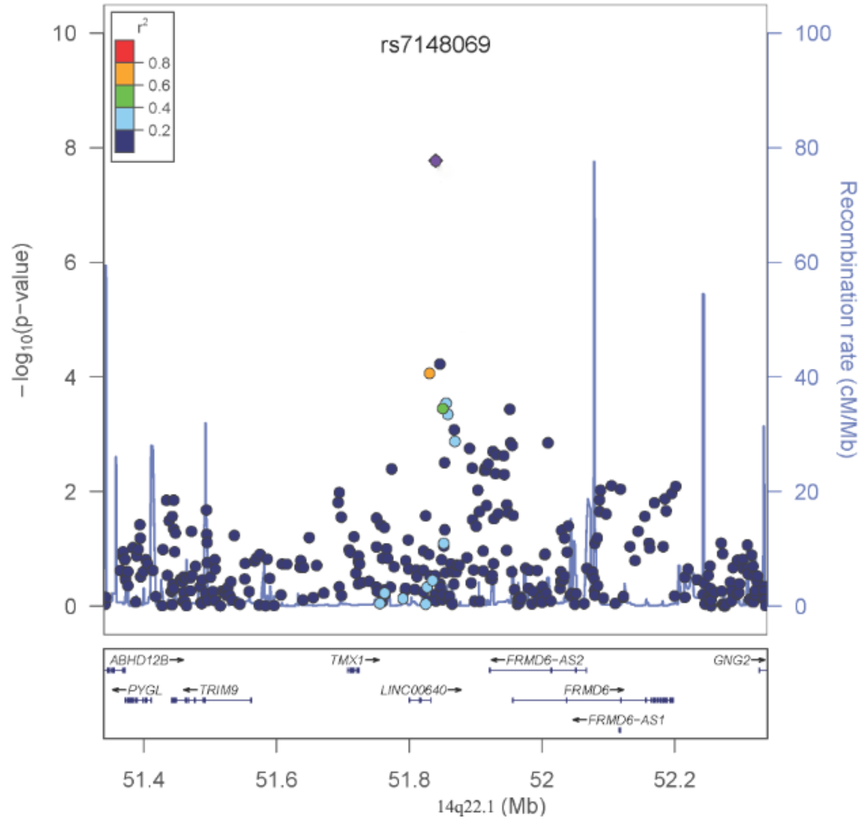

(m): 14q32.13 (rs1243572)

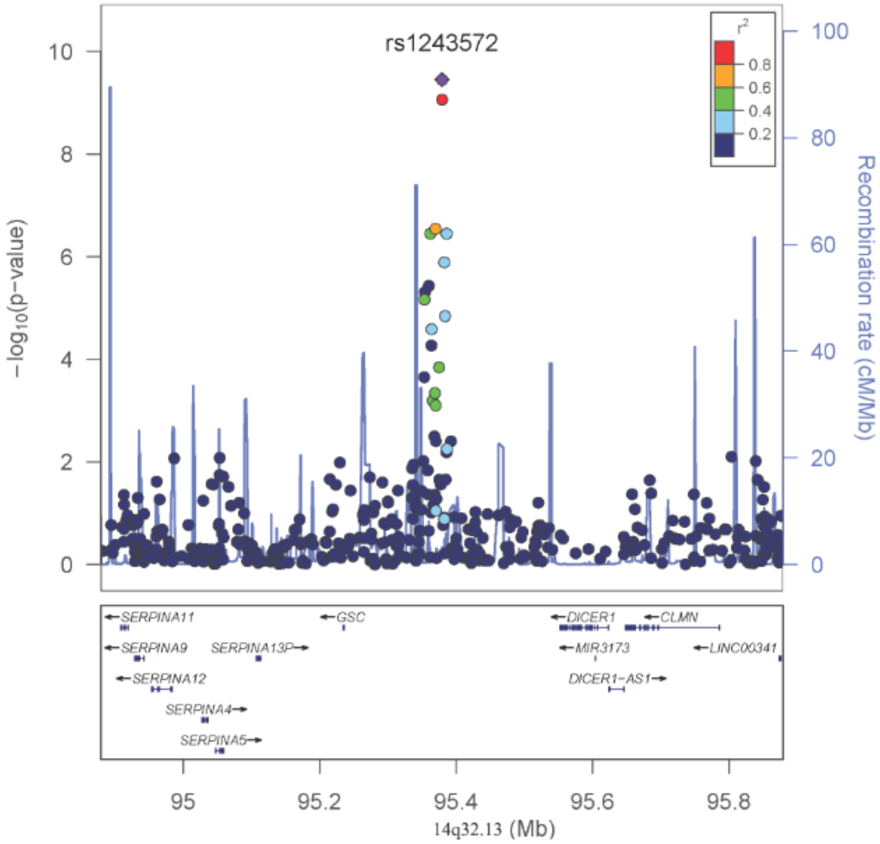

(n): 17q21.32 (rs1838105)

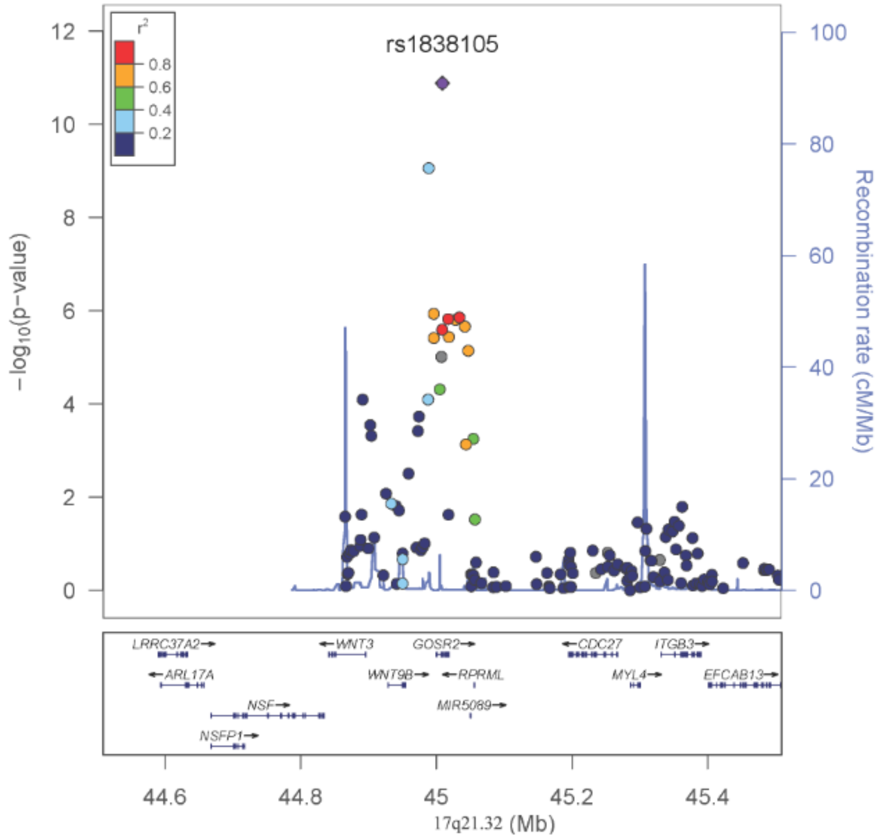

Supplementary Figure 5 | The scatter plots of the association evidence within 14 loci for NSCLP identified in the current study. The 14 loci are as following: a: 2p25.1 (rs287982); b: 4p16.2 (rs1907989); c: 4q28.1 (rs908822); d: 5p12 (rs10462065); e: 6p24.3 (rs9381107); f: 8p11.23 (rs13317); g: 8q22.1 (rs957448); h: 9q22.32 (rs10512248); i: 12q13.13 (rs3741442); j: 12q13.2 (rs705704); k: 12q21.1 (rs2304269); l: 14q22.1 (rs7148069); m: 14q32.13 (rs1243572); n: 17q21.32 (rs1838105). The P values of SNPs (shown as $-\log_{10}$ values in y-axis, obtained using the Cochran-Armitage trend test) were plotted against their chromosomal map positions (x-axis). The color of each SNP spot reflects its r^2 with the top SNP (large purple diamond) within each association locus, changing from red to blue. Estimated recombination rates were plotted in light blue. Gene annotations were adapted from the University of California at Santa Cruz Genome Browser (http://genome.ucsc.edu/).

a.

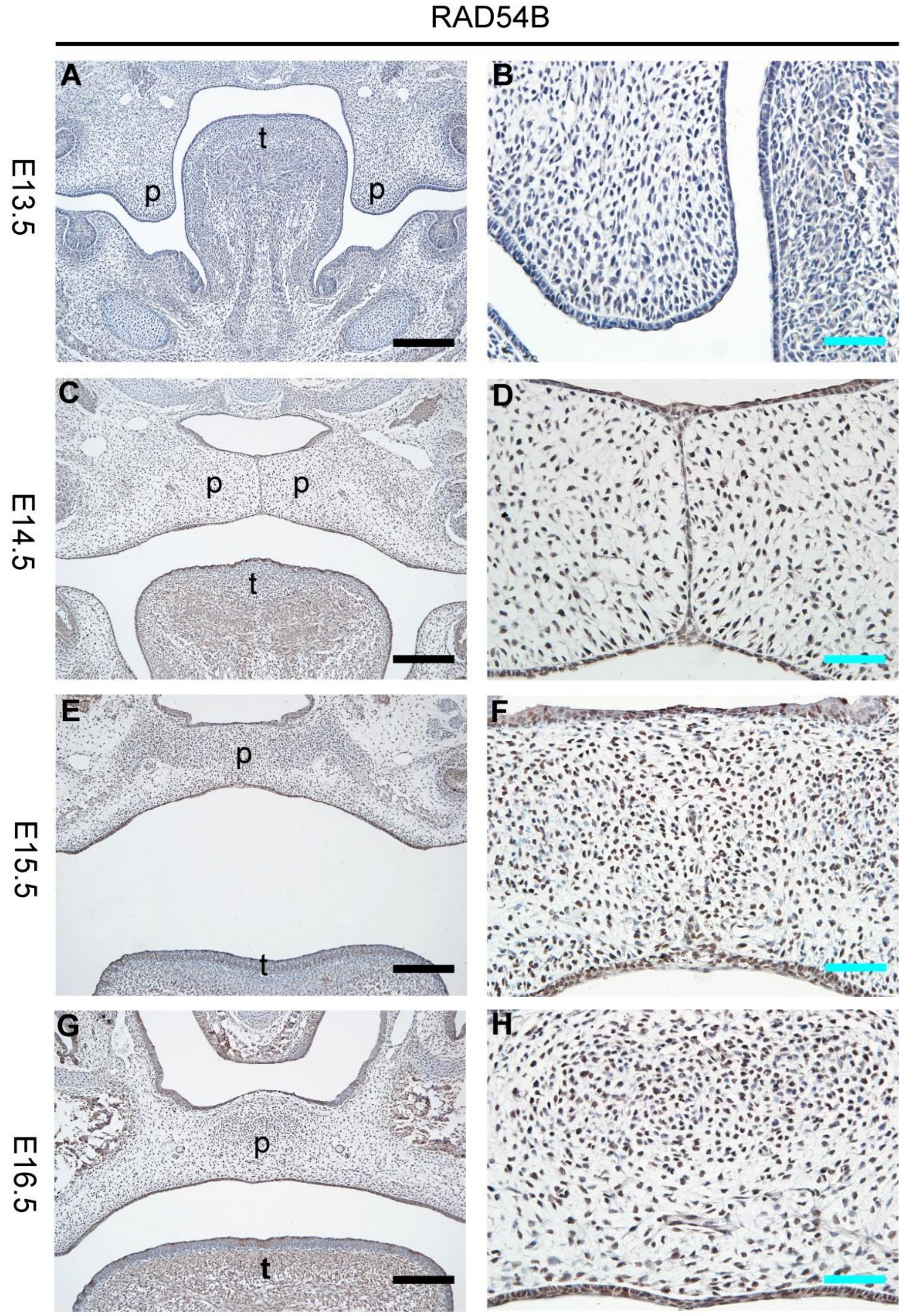

b.

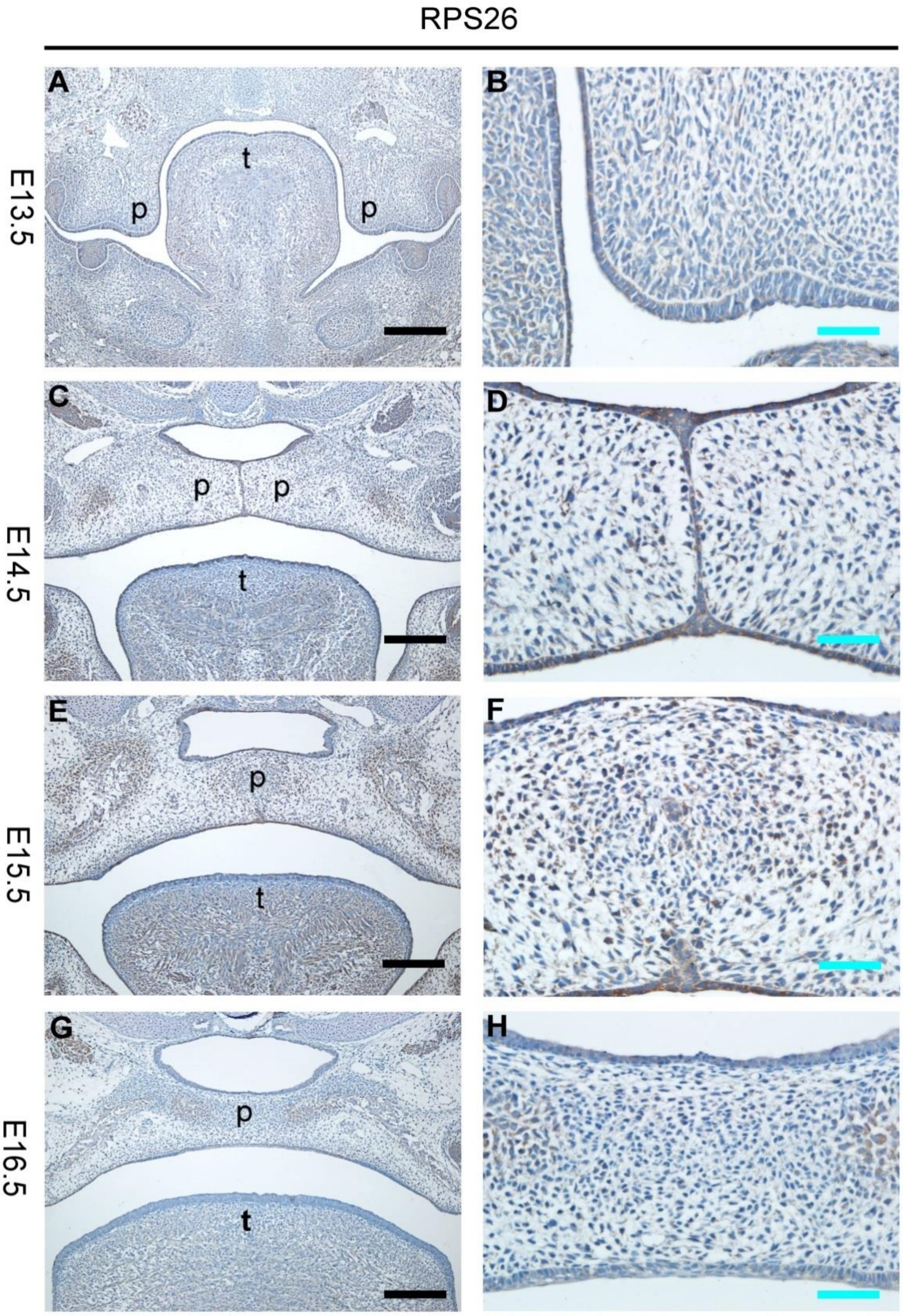

c.

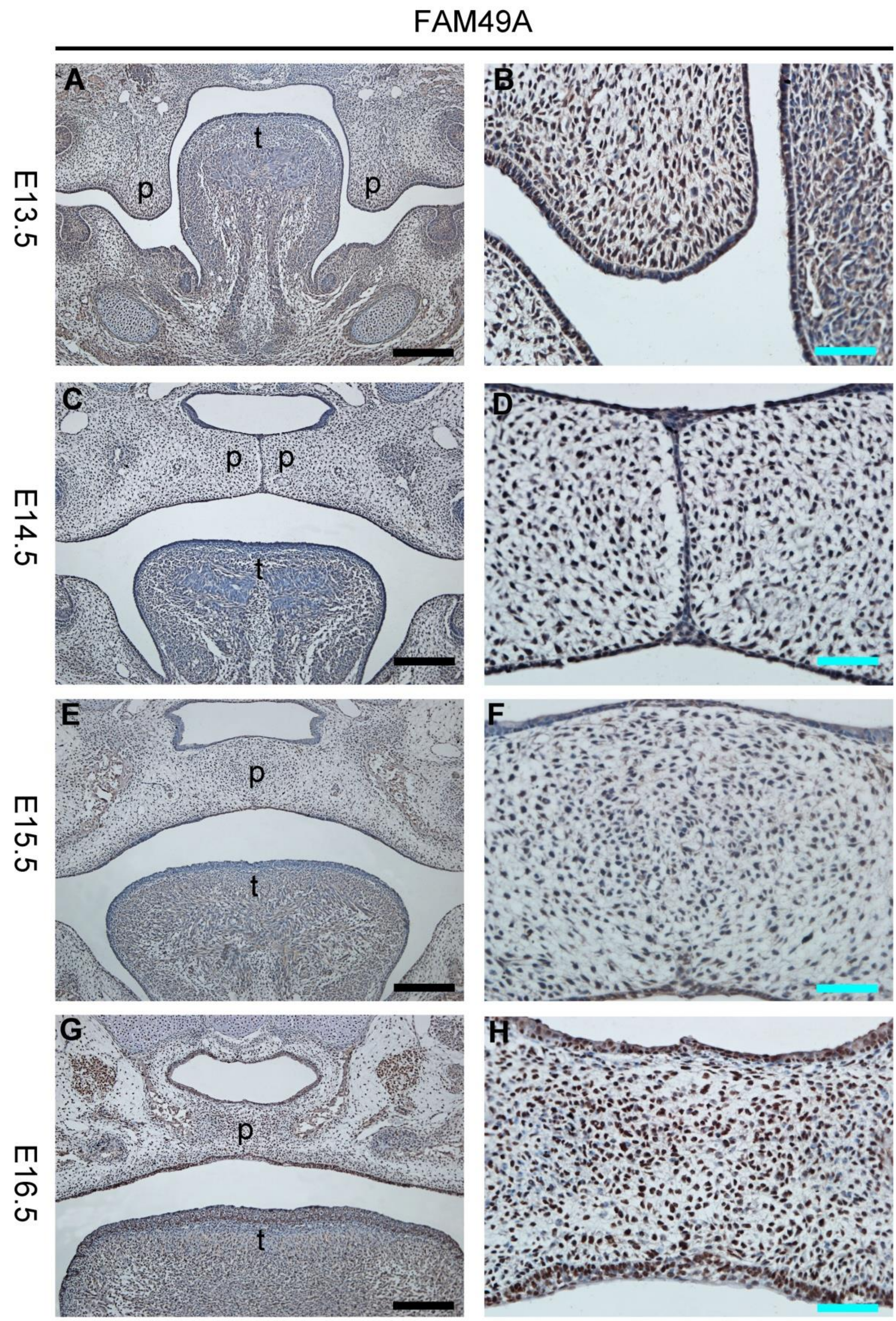

Supplementary Figure 6 | Expression studies in the mouse. Immunohistochemistry on mouse embryos from E13.5 to E16.5 shows: a. Rad54b localized in the nucleus of the palatal mesenchymal cells and epithelium cells (C-H) including the MEE during palatal fusion on E14.5 (D). Rad54b expression in palatal tissues increased with development (B, D, F, H). Rps26 localized in the cytoplasm of palatal mesenchymal cells and epithelium cells (A-H) including the MEE during palatal fusion on E14.5 (D). b. Rps26 showed increased expression from E13.5 to E15.5 (A-F) but decreased at E16.5 (G, H). c. Fam49a localized in the nucleus of palatal mesenchymal cells and epithelium cells (A-H). Positive results were stronger at E16.5 in palatal tissues (G, H). n =5; p, palatal shelf; t, tongue; Scale bars: black, 200 μm; green, 50 μm. B, D, F and H are the magnification of the palate in A, C, E and G.

Supplementary Tables:

Supplementary Table 1. Samples summary in the genome-wide association and replication studies.

				Cases		Controls	
**Stage**	**Cohort**	**Ethnicity**	**Phenotype**	**Sample size**	**Male/Female (%)**	**Sample size**	**Male/Female (%)**
GWAS	cohort 1	Chinese	NSCLP	2,096	72.0/28.0	4,051	58.7/41.3
Stage 2	cohort 2	Chinese	NSCLP	1,346	72.8/27.2	4,542	65.7/34.3
Stage 3	cohort 3	Chinese	NSCLO	1,104	61.6/38.4	3,312*	51.8/48.2
	cohort 4	Chinese	NSCPO	1,104	41.6/58.4	3,312*	51.8/48.2
Stage 4	cohort 5	Central European	NSCL/P	399	62.4/37.6	1,318	50.8/49.2
	cohort 6	Asian	NSCL/P	861 case-parent trios	64.5/35.5	NA	NA
	cohort 7	European American	NSCL/P	557 case-parent trios	64.8/36.2	NA	NA

NSCLP: non-syndromic cleft lip with palate; NSCLO: non-syndromic cleft lip only;
NSCPO: non-syndromic cleft palate only; NSCL/P: non-syndromic cleft lip with or without palate.
*The 3,312 controls were shared in cohort 3 and 4.

Supplementary Table 2. Association results of 146 SNPs in GWAS, NSCLP Replication and Meta analysis of NSCLP.

Loci	SNP	BP (hg19)	Alleles	GWAS				NSCLP Replication				Meta analysis		
				F_A	F_U	*P*	OR	F_A	F_U	*P*	OR	*P*(F)	OR(F)	P_{het}*
1p31.3	rs7554547	11973888	G/A	0.45	0.52	2.89E-05	0.78 (0.72-0.84)	0.47	0.47	9.39E-01	1.00 (0.91-1.09)	5.47E-07	0.86	0.0000
1p36.13	rs9439713	18972776	A/G	0.06	0.04	2.05E-06	1.56 (1.31-1.86)	0.05	0.04	2.06E-01	1.14 (0.93-1.39)	5.87E-06	1.36	0.0453
1p36.13	rs742071	18979874	A/C	0.06	0.04	4.73E-05	1.47 (1.24-1.74)	0.05	0.04	9.17E-03	1.30 (1.01-1.59)	4.26E-07	1.40	0.4859
1p36.13	rs11582254	20018077	A/G	0.37	0.42	1.84E-06	0.80 (0.74-0.87)	0.40	0.41	2.96E-01	0.95 (0.87-1.04)	8.69E-07	0.86	0.0106
1p36.13	rs1883567	20020466	A/C	0.36	0.41	1.02E-05	0.81 (0.75-0.88)	0.39	0.40	4.01E-01	0.96 (0.88-1.05)	8.13E-06	0.88	0.0139
1p32.2	rs6588607	56586286	A/C	0.22	0.25	5.70E-06	0.83 (0.76-0.91)	0.24	0.24	8.86E-01	1.01 (0.91-1.11)	3.78E-03	0.91	0.0152
1p32.2	rs1331862	56642998	G/A	0.23	0.26	3.52E-06	0.83 (0.76-0.91)	0.26	0.25	3.16E-01	1.05 (0.95-1.16)	2.22E-02	0.93	0.0025
1p31.1	rs277354	75291073	G/A	0.23	0.26	4.13E-05	0.86 (0.79-0.94)	0.25	0.25	8.47E-01	0.99 (0.90-1.09)	7.86E-03	0.91	0.0681
1p31.1	rs7367789	75357094	A/G	0.21	0.24	1.67E-05	0.84 (0.77-0.92)	0.23	0.23	9.62E-01	1.00 (0.90-1.11)	4.41E-03	0.91	0.0319
1p22.1	rs4147827	94548080	C/G	0.08	0.05	4.86E-05	1.41 (1.21-1.64)	0.08	0.06	3.98E-03	1.28 (1.08-1.51)	1.89E-07	1.35	0.5255
1p22.1	rs17461953	94551450	C/A	0.08	0.05	2.68E-05	1.42 (1.22-1.65)	0.08	0.06	3.01E-03	1.29 (1.09-1.52)	7.04E-08	1.36	0.5006
1p22.1	rs481931	94570016	A/C	0.33	0.37	4.82E-06	0.83 (0.77-0.90)	0.33	0.39	2.79E-08	0.77 (0.71-0.85)	1.06E-12	0.80	0.3687
1p22.1	rs4147803	94582293	G/C	0.29	0.33	1.22E-06	0.81 (0.75-0.88)	0.29	0.34	2.27E-06	0.80 (0.73-0.88)	7.97E-12	0.81	0.8369
1p13.3	rs12047887	111144007	A/G	0.17	0.15	4.35E-05	1.15 (1.04-1.28)	0.17	0.14	2.16E-04	1.25 (1.11-1.40)	7.78E-06	1.19	0.4277
1p13.3	rs4839542	111155595	A/G	0.16	0.14	3.44E-05	1.19 (1.07-1.32)	0.16	0.13	1.41E-03	1.22 (1.08-1.38)	6.74E-06	1.20	0.8090
1q23.3	rs10918593	162030654	G/A	0.33	0.29	1.31E-05	1.22 (1.12-1.32)	0.31	0.31	6.58E-01	1.02 (0.93-1.12)	1.19E-04	1.13	0.0147
1q32.2	rs861020	209977111	A/G	0.24	0.19	6.38E-12	1.34 (1.23-1.47)	0.23	0.19	6.63E-06	1.27 (1.15-1.41)	1.30E-14	1.31	0.5428
1q32.2	rs642961	209989270	A/G	0.24	0.19	3.86E-12	1.35 (1.23-1.48)	0.23	0.19	2.23E-06	1.29 (1.16-1.43)	2.76E-15	1.32	0.6061
1q32.2	rs2064163	210048819	A/C	0.38	0.44	1.04E-09	0.77 (0.71-0.83)	0.38	0.44	8.18E-09	0.77 (0.71-0.84)	8.60E-19	0.77	0.9625
1q32.2	rs9430019	210050794	A/G	0.31	0.26	1.77E-07	1.27 (1.17-1.38)	0.30	0.26	2.58E-05	1.23 (1.12-1.35)	1.68E-12	1.25	0.6420
1q32.2	rs11583395	218841163	A/G	0.49	0.44	4.04E-05	1.21 (1.12-1.30)	0.47	0.46	2.66E-01	1.05 (0.96-1.15)	1.11E-05	1.14	0.0378
1q32.2	rs201830371	218845278	A/G	0.49	0.44	5.57E-05	1.20 (1.12-1.30)	0.47	0.47	7.87E-01	1.01 (0.93-1.10)	1.42E-04	1.12	0.0098

2p25.1	rs287980	9971366	G/A	0.23	0.27	1.99E-05	0.82 (0.75-0.89)	0.23	0.27	5.54E-04	0.84 (0.76-0.93)	1.94E-08	0.83	0.8120
2p25.1	rs287982	9972442	G/A	0.23	0.27	1.58E-05	0.82 (0.75-0.89)	0.23	0.27	2.28E-04	0.83 (0.75-0.92)	6.15E-09	0.82	0.8981
2p24.2	rs10172734	16733054	G/A	0.26	0.32	3.85E-13	0.76 (0.70-0.83)	0.26	0.33	1.58E-11	0.72 (0.65-0.79)	2.89E-20	0.74	0.4992
2p24.2	rs7552	16733928	A/G	0.24	0.30	1.57E-14	0.74 (0.68-0.81)	0.24	0.31	5.64E-12	0.71 (0.64-0.78)	5.83E-22	0.73	0.5814
2p22.2	rs7590255	37990079	G/A	0.03	0.02	2.73E-06	1.83 (1.43-2.34)	0.02	0.02	1.26E-01	1.25 (0.94-1.67)	3.71E-06	1.56	0.0896
2q21.1	rs17491637	130176577	A/G	0.12	0.16	1.82E-08	0.71 (0.63-0.80)	0.16	0.15	3.60E-01	1.06 (0.94-1.19)	4.89E-04	0.86	0.0000
2q32.1	rs11677862	184898361	A/G	0.38	0.42	1.17E-05	0.82 (0.76-0.89)	0.41	0.41	8.72E-01	0.99 (0.91-1.09)	1.26E-04	0.89	0.0070
2q32.1	rs12623132	184954425	A/C	0.40	0.44	2.10E-06	0.83 (0.77-0.90)	0.41	0.43	1.63E-01	0.94 (0.86-1.03)	1.00E-05	0.88	0.0789
2q35	rs6734545	220653996	A/C	0.20	0.18	8.61E-05	1.16 (1.05-1.27)	0.21	0.17	4.61E-05	1.25 (1.13-1.40)	8.03E-07	1.20	0.3864
2q37.1	rs77866552	233552664	A/G	0.02	0.04	1.73E-06	0.50 (0.39-0.64)	0.03	0.04	8.74E-02	0.81 (0.64-1.03)	7.08E-07	0.64	0.0165
3p24.3	rs78991648	17152071	A/G	0.22	0.26	6.30E-05	0.83 (0.76-0.90)	0.26	0.24	3.64E-02	1.11 (1.01-1.23)	8.23E-02	0.94	0.0000
3p23	rs1395165	31178850	A/G	0.42	0.47	7.82E-05	0.83 (0.77-0.89)	0.44	0.44	7.58E-01	1.01 (0.93-1.11)	4.65E-04	0.90	0.0021
3p23	rs12485312	31219954	A/G	0.41	0.46	5.96E-05	0.83 (0.77-0.90)	0.43	0.43	8.86E-01	1.01 (0.92-1.10)	5.27E-04	0.90	0.0049
3q12.1	rs1038294	99503728	A/G	0.13	0.17	1.18E-07	0.73 (0.66-0.81)	0.15	0.16	2.12E-01	0.93 (0.82-1.05)	4.47E-07	0.81	0.0127
3q12.1	rs1688766	99647850	A/G	0.13	0.17	1.32E-07	0.73 (0.65-0.81)	0.15	0.16	2.28E-01	0.93 (0.82-1.05)	4.83E-07	0.81	0.0113
3q13.31	rs9847858	117141815	A/G	0.32	0.35	8.86E-05	0.85 (0.78-0.92)	0.33	0.33	5.87E-01	1.03 (0.94-1.12)	6.63E-03	0.92	0.0068
3q26.2	rs10936656	170568001	A/C	0.34	0.37	7.47E-05	0.86 (0.79-0.93)	0.36	0.37	2.53E-01	0.95 (0.87-1.04)	3.30E-04	0.90	0.1672
3q29	rs17597256	196767854	G/A	0.24	0.27	7.80E-05	0.85 (0.78-0.93)	0.24	0.27	1.24E-02	0.88 (0.80-0.97)	1.48E-05	0.86	0.7255
3q29	rs7649443	196931821	A/G	0.24	0.27	8.71E-05	0.86 (0.79-0.94)	0.25	0.27	4.39E-03	0.87 (0.78-0.96)	9.23E-06	0.86	0.9600
4p16.2	rs34246903	4794195	C/A	0.38	0.43	2.57E-05	0.82 (0.76-0.89)	0.40	0.43	1.09E-02	0.89 (0.82-0.97)	4.45E-08	0.85	0.2344
4p16.2	rs1907989	4818925	A/G	0.46	0.51	2.29E-06	0.81 (0.75-0.87)	0.48	0.50	1.94E-02	0.90 (0.83-0.98)	1.58E-08	0.85	0.1130
4q28.1	rs7692299	124710584	A/G	0.09	0.06	3.72E-07	1.49 (1.30-1.73)	0.07	0.07	2.60E-01	1.10 (0.93-1.31)	9.29E-07	1.32	0.0180
4q28.1	rs908822	124906257	A/G	0.10	0.07	6.13E-07	1.46 (1.28-1.66)	0.10	0.09	4.46E-02	1.16 (1.00-1.34)	4.33E-08	1.31	0.0545
4q31.3	rs7686589	151337243	A/G	0.37	0.33	3.08E-05	1.18 (1.09-1.28)	0.36	0.35	4.63E-01	1.03 (0.95-1.13)	3.05E-04	1.12	0.0563
4q31.3	rs66509882	151543429	C/A	0.37	0.33	3.41E-06	1.20 (1.11-1.30)	0.35	0.35	9.55E-01	1.00 (0.92-1.10)	4.71E-04	1.11	0.0102
4q32.1	rs4417922	157006346	G/A	0.30	0.34	1.55E-06	0.82 (0.76-0.89)	0.32	0.32	9.49E-01	1.00 (0.91-1.09)	4.59E-04	0.90	0.0085

4q34.3	rs2333571	177761552	A/G	0.34	0.36	7.18E-05	0.89 (0.82-0.96)	0.35	0.38	3.41E-02	0.91 (0.83-0.99)	3.33E-04	0.90	0.8141
4q34.3	rs13124707	177802518	G/A	0.35	0.38	6.52E-05	0.88 (0.82-0.96)	0.36	0.39	7.10E-03	0.88 (0.81-0.97)	4.48E-05	0.88	0.9965
5p12	rs10462065	44068846	A/C	0.24	0.20	3.51E-07	1.26 (1.15-1.38)	0.23	0.20	2.04E-03	1.18 (1.06-1.31)	1.12E-08	1.22	0.4835
5p12	rs4242107	44129083	A/G	0.44	0.40	6.08E-07	1.17 (1.09-1.26)	0.43	0.40	7.44E-03	1.13 (1.03-1.23)	1.32E-06	1.15	0.5979
5p12	rs10462084	44901349	G/A	0.03	0.05	2.18E-05	0.68 (0.55-0.83)	0.04	0.05	3.78E-02	0.80 (0.64-0.99)	2.52E-05	0.73	0.3877
6p24.3	rs7771515	9442330	A/G	0.35	0.40	2.41E-09	0.80 (0.74-0.87)	0.38	0.40	1.17E-01	0.93 (0.85-1.02)	2.33E-07	0.86	0.0322
6p24.3	rs9381107	9469238	A/G	0.31	0.37	1.97E-09	0.79 (0.73-0.86)	0.33	0.36	1.40E-02	0.89 (0.81-0.98)	2.72E-09	0.83	0.0900
6p24.3	rs11965246	9959207	C/A	0.04	0.07	1.21E-05	0.61 (0.51-0.73)	0.07	0.06	2.16E-01	1.12 (0.94-1.33)	4.09E-03	0.83	0.0000
6p23	rs12203226	14951008	A/C	0.43	0.39	3.24E-06	1.20 (1.11-1.30)	0.39	0.40	2.17E-01	0.95 (0.87-1.03)	6.66E-03	1.08	0.0006
7p21.2	rs10260512	14856700	A/G	0.40	0.44	4.98E-05	0.87 (0.80-0.94)	0.40	0.41	2.39E-01	0.95 (0.87-1.04)	4.61E-04	0.90	0.2182
7p21.1	rs76523544	19308308	C/A	0.08	0.11	2.17E-05	0.67 (0.58-0.77)	0.10	0.09	6.88E-01	1.03 (0.89-1.19)	9.80E-05	0.82	0.0000
7q36.2	rs10256809	155079596	A/G	0.07	0.10	3.19E-07	0.65 (0.56-0.75)	0.10	0.09	2.68E-01	1.09 (0.94-1.26)	6.84E-04	0.84	0.0000
8p22	rs17227506	13458794	A/G	0.39	0.44	8.24E-06	0.82 (0.76-0.89)	0.40	0.42	1.46E-01	0.94 (0.86-1.02)	2.70E-06	0.87	0.0575
8p11.23	rs13317	38269514	G/A	0.30	0.34	4.10E-06	0.83 (0.77-0.90)	0.32	0.35	7.43E-03	0.88 (0.80-0.97)	3.96E-08	0.85	0.4601
8p11.23	rs3925	38281658	A/G	0.27	0.32	1.47E-06	0.81 (0.74-0.88)	0.30	0.32	3.24E-02	0.90 (0.82-0.99)	2.66E-07	0.85	0.1398
8q21.3	rs12543318	88868340	A/C	0.32	0.38	2.43E-07	0.78 (0.72-0.84)	0.33	0.37	7.83E-04	0.86 (0.78-0.94)	8.80E-12	0.81	0.2050
8q21.3	rs1034832	88918331	C/A	0.31	0.36	5.92E-07	0.79 (0.73-0.85)	0.32	0.35	1.65E-03	0.86 (0.79-0.95)	1.35E-10	0.82	0.2243
8q22.1	rs12681366	95401265	G/A	0.44	0.49	2.95E-06	0.82 (0.76-0.88)	0.44	0.48	2.48E-04	0.85 (0.78-0.93)	2.35E-10	0.83	0.5965
8q22.1	rs957448	95541302	G/A	0.44	0.50	3.78E-10	0.78 (0.72-0.84)	0.46	0.49	7.59E-04	0.86 (0.79-0.94)	9.60E-13	0.81	0.1260
8q22.2	rs11774066	99455311	A/G	0.42	0.38	8.65E-05	1.18 (1.09-1.28)	0.42	0.39	3.85E-03	1.14 (1.04-1.24)	3.39E-07	1.16	0.6166
8q22.2	rs1487022	100529826	A/C	0.44	0.39	5.64E-05	1.19 (1.10-1.28)	0.44	0.41	1.43E-03	1.15 (1.06-1.26)	7.84E-08	1.17	0.7214
8q24.12	rs1993392	121240646	G/A	0.13	0.16	6.63E-06	0.78 (0.70-0.87)	0.15	0.15	4.74E-01	0.96 (0.85-1.08)	1.57E-04	0.85	0.0319
8q24.21	rs7845615	129888794	A/G	0.22	0.18	5.11E-06	1.28 (1.17-1.41)	0.20	0.17	8.59E-05	1.25 (1.12-1.39)	1.03E-10	1.27	0.8110
8q24.21	rs7017252	129950844	A/G	0.08	0.05	3.27E-06	1.59 (1.36-1.85)	0.07	0.05	4.18E-08	1.62 (1.36-1.93)	8.47E-16	1.60	0.8960
9p24.1	rs327753	7793309	G/A	0.28	0.32	8.34E-05	0.83 (0.77-0.90)	0.30	0.30	9.01E-01	1.01 (0.92-1.11)	1.45E-03	0.90	0.0094
9p24.1	rs2990600	7796329	C/A	0.27	0.31	3.47E-05	0.82 (0.76-0.89)	0.30	0.29	7.55E-01	1.02 (0.92-1.12)	1.26E-03	0.90	0.0043

9p23	rs408960	9069839	G/A	0.45	0.42	5.34E-05	1.14 (1.05-1.23)	0.45	0.45	8.99E-01	1.01 (0.92-1.10)	1.02E-02	1.08	0.0717
9p23	rs324475	9077607	C/A	0.44	0.41	9.27E-05	1.13 (1.05-1.22)	0.44	0.44	7.77E-01	1.01 (0.93-1.10)	1.05E-02	1.08	0.1111
9p23	rs77401103	10546603	G/A	0.06	0.09	5.74E-07	0.64 (0.55-0.75)	0.08	0.09	7.84E-01	0.98 (0.84-1.14)	3.54E-05	0.79	0.0009
9p23	rs542733	14098867	G/A	0.40	0.45	1.75E-05	0.84 (0.76-0.90)	0.43	0.42	5.58E-01	1.03 (0.94-1.12)	2.31E-03	0.91	0.0024
9p23	rs498010	14116484	A/G	0.29	0.33	6.24E-05	0.83 (0.77-0.90)	0.30	0.31	9.47E-01	1.00 (0.91-1.10)	9.05E-04	0.90	0.0136
9p21.3	rs35825512	20167602	A/G	0.03	0.05	5.39E-05	0.63 (0.51-0.77)	0.04	0.03	1.38E-02	1.32 (1.06-1.64)	1.43E-01	0.89	0.0000
9p21.3	rs7863754	20168739	A/G	0.03	0.05	4.11E-05	0.63 (0.51-0.78)	0.04	0.03	9.06E-03	1.34 (1.07-1.67)	1.88E-01	0.90	0.0000
9q22.2	rs7871395	92209587	A/G	0.30	0.26	4.54E-05	1.23 (1.13-1.34)	0.29	0.26	1.07E-03	1.17 (1.07-1.29)	6.06E-09	1.21	0.5782
9q22.32	rs10512248	98259703	C/A	0.29	0.34	1.80E-07	0.79 (0.73-0.86)	0.30	0.33	3.19E-03	0.87 (0.79-0.95)	5.10E-10	0.82	0.2026
9q22.32	rs28361526	98295863	A/G	0.30	0.34	1.65E-05	0.83 (0.77-0.90)	0.31	0.34	3.02E-02	0.90 (0.82-0.99)	1.95E-06	0.86	0.2949
9q33.1	rs13294988	119099873	G/A	0.47	0.43	1.23E-05	1.17 (1.08-1.26)	0.45	0.43	2.22E-02	1.11 (1.02-1.21)	6.68E-06	1.14	0.4994
10q24.2	rs474558	99695194	A/C	0.26	0.20	1.43E-12	1.40 (1.28-1.53)	0.25	0.26	2.62E-01	0.94 (0.86-1.04)	5.05E-06	1.17	0.0000
10q25.3	rs6585429	118893231	G/A	0.39	0.44	4.92E-08	0.81 (0.75-0.87)	0.39	0.45	2.22E-06	0.81 (0.74-0.88)	7.14E-13	0.81	0.9967
11q13.3	rs12792211	69025553	A/G	0.26	0.23	9.52E-05	1.21 (1.11-1.32)	0.26	0.24	1.69E-01	1.07 (0.97-1.18)	4.90E-05	1.15	0.1364
12q13.13	rs3741442	53346750	G/A	0.46	0.41	1.17E-06	1.22 (1.13-1.32)	0.46	0.41	3.36E-06	1.23 (1.13-1.34)	3.72E-12	1.22	0.9598
12q13.2	rs705704	56435412	A/G	0.27	0.23	4.39E-05	1.22 (1.12-1.33)	0.28	0.24	5.97E-05	1.22 (1.11-1.34)	1.29E-09	1.22	0.9839
12q21.1	rs2304269	72080272	G/A	0.38	0.44	1.07E-07	0.79 (0.73-0.85)	0.41	0.45	1.12E-04	0.84 (0.77-0.92)	1.32E-12	0.81	0.3903
12q21.1	rs7967428	72089040	G/A	0.38	0.44	1.63E-07	0.79 (0.73-0.85)	0.41	0.45	1.57E-04	0.84 (0.77-0.92)	3.08E-12	0.81	0.3871
12q24.11	rs2188380	111386127	G/A	0.12	0.17	5.75E-09	0.67 (0.60-0.74)	0.15	0.15	9.18E-01	1.01 (0.89-1.14)	1.49E-07	0.80	0.0000
12q24.11	rs12229654	111414461	C/A	0.13	0.18	2.59E-08	0.68 (0.61-0.76)	0.15	0.16	4.22E-01	0.95 (0.85-1.07)	1.06E-08	0.79	0.0000
12q24.11	rs4766453	111692124	A/G	0.51	0.47	5.13E-05	1.20 (1.12-1.30)	0.49	0.47	1.92E-01	1.06 (0.97-1.15)	7.76E-06	1.14	0.0563
12q24.13	rs11066150	112518803	A/G	0.34	0.42	5.21E-09	0.72 (0.67-0.78)	0.35	0.37	7.32E-02	0.92 (0.84-1.01)	3.41E-13	0.80	0.0006
12q24.13	rs12229892	112923393	A/G	0.41	0.48	7.86E-09	0.75 (0.69-0.80)	0.43	0.44	2.92E-01	0.95 (0.87-1.04)	1.84E-10	0.83	0.0000
13q31.1	rs9545308	80639405	A/C	0.15	0.12	4.09E-05	1.30 (1.17-1.45)	0.14	0.11	2.34E-04	1.27 (1.12-1.44)	2.00E-09	1.29	0.8103
14q13.2	rs1168980	36400983	C/A	0.16	0.20	3.49E-05	0.80 (0.73-0.88)	0.20	0.19	1.63E-01	1.08 (0.97-1.21)	2.18E-02	0.92	0.0005
14q13.2	rs17103683	36426134	A/G	0.18	0.21	7.09E-06	0.79 (0.71-0.86)	0.21	0.20	1.65E-01	1.08 (0.97-1.20)	7.12E-03	0.91	0.0000

14q13.3	rs1956424	37531902	A/G	0.27	0.24	2.33E-07	1.19 (1.10-1.30)	0.25	0.25	9.29E-01	1.01 (0.91-1.11)	1.91E-03	1.11	0.0265
14q13.3	rs10133673	37537352	G/A	0.27	0.24	4.49E-07	1.19 (1.09-1.29)	0.25	0.23	4.25E-03	1.16 (1.05-1.28)	1.44E-06	1.17	0.8053
14q22.1	rs7148069	51839645	A/G	0.23	0.19	1.94E-05	1.27 (1.16-1.40)	0.22	0.20	7.21E-03	1.16 (1.04-1.28)	1.69E-08	1.22	0.2538
14q22.1	rs10134081	51848843	G/A	0.12	0.09	5.00E-06	1.41 (1.25-1.59)	0.12	0.10	8.72E-02	1.13 (0.98-1.29)	1.23E-07	1.28	0.0382
14q24.3	rs9671386	75151047	G/A	0.27	0.30	7.93E-05	0.87 (0.80-0.94)	0.28	0.28	9.95E-01	1.00 (0.91-1.10)	1.12E-02	0.92	0.0534
14q32.13	rs1243572	95379499	G/A	0.48	0.42	1.37E-07	1.26 (1.17-1.36)	0.45	0.42	6.09E-03	1.13 (1.04-1.23)	3.52E-10	1.20	0.1138
14q32.13	rs1243573	95379583	C/A	0.48	0.42	2.83E-07	1.25 (1.16-1.35)	0.45	0.42	7.30E-03	1.13 (1.03-1.23)	8.61E-10	1.20	0.1178
15q22.2	rs78512626	61432877	C/A	0.12	0.15	8.05E-06	0.76 (0.67-0.85)	0.14	0.15	3.84E-01	0.95 (0.84-1.07)	7.02E-05	0.84	0.0261
15q24.1	rs2289187	74744399	G/A	0.55	0.48	2.73E-08	1.33 (1.24-1.44)	0.48	0.50	1.34E-01	0.94 (0.86-1.02)	3.86E-11	1.21	0.0005
15q24.1	rs6495117	74899500	G/A	0.45	0.52	2.95E-08	0.75 (0.69-0.81)	0.48	0.50	1.70E-01	0.94 (0.86-1.03)	6.29E-11	0.83	0.0005
16p13.3	rs2283487	3969886	G/A	0.42	0.47	3.18E-06	0.83 (0.76-0.89)	0.43	0.47	3.91E-05	0.83 (0.76-0.91)	1.27E-10	0.83	0.9121
16p13.3	rs17136624	3996282	A/G	0.25	0.22	8.43E-05	1.21 (1.11-1.32)	0.27	0.23	3.11E-06	1.28 (1.15-1.42)	3.82E-10	1.24	0.5269
16p13.3	rs4786640	5243669	C/A	0.52	0.48	9.15E-05	1.19 (1.10-1.28)	0.48	0.49	2.78E-01	0.95 (0.87-1.04)	6.89E-03	1.08	0.0005
16p13.11	rs7198766	16105965	G/A	0.24	0.29	7.95E-05	0.78 (0.71-0.85)	0.26	0.25	8.41E-02	1.09 (0.99-1.20)	1.92E-03	0.90	0.0000
16p13.11	rs7188937	16118620	A/C	0.24	0.29	6.91E-05	0.78 (0.72-0.85)	0.26	0.25	2.01E-01	1.07 (0.97-1.18)	6.08E-04	0.89	0.0000
16q22.2	rs217181	72114002	A/G	0.32	0.29	2.05E-05	1.15 (1.06-1.25)	0.32	0.31	5.06E-02	1.10 (1.00-1.20)	1.34E-04	1.13	0.5508
17p13.1	rs2872615	8914693	G/A	0.43	0.48	7.45E-06	0.80 (0.74-0.87)	0.43	0.47	1.06E-04	0.84 (0.77-0.92)	8.81E-12	0.82	0.5224
17p13.1	rs1880646	8929845	A/G	0.52	0.46	4.98E-06	1.25 (1.16-1.35)	0.52	0.48	2.60E-04	1.17 (1.08-1.28)	1.69E-11	1.22	0.4104
17q21.32	rs4968247	44988703	A/G	0.37	0.41	4.11E-06	0.84 (0.78-0.91)	0.37	0.41	2.43E-05	0.83 (0.76-0.90)	8.70E-10	0.83	0.8605
17q21.32	rs1838105	45008935	A/G	0.45	0.39	1.84E-07	1.26 (1.17-1.36)	0.42	0.38	3.48E-04	1.17 (1.08-1.28)	1.31E-11	1.22	0.3543
17q22	rs12951993	54760614	A/G	0.36	0.32	1.19E-05	1.21 (1.12-1.31)	0.36	0.33	2.88E-02	1.11 (1.01-1.21)	5.26E-07	1.16	0.2119
17q22	rs227731	54773238	C/A	0.36	0.32	1.74E-05	1.22 (1.12-1.32)	0.36	0.32	1.19E-03	1.16 (1.06-1.27)	8.83E-09	1.19	0.5623
18q11.2	rs3903236	22122490	C/A	0.29	0.33	2.87E-05	0.82 (0.76-0.89)	0.32	0.32	9.35E-01	1.00 (0.91-1.09)	3.64E-04	0.89	0.0077
18q11.2	rs79280643	22126108	A/G	0.29	0.33	6.64E-05	0.82 (0.76-0.90)	0.32	0.32	7.53E-01	0.99 (0.90-1.08)	2.79E-04	0.89	0.0136
18q22.3	rs117386717	69155820	G/A	0.02	0.03	7.86E-07	0.56 (0.43-0.72)	0.03	0.04	5.89E-01	0.94 (0.74-1.19)	8.42E-04	0.74	0.0111
19p13.11	rs739461	19715794	A/G	0.21	0.25	4.52E-05	0.81 (0.74-0.89)	0.22	0.23	3.37E-01	0.95 (0.86-1.05)	4.24E-05	0.87	0.0456

20q12	rs6129653	39275603	A/G	0.35	0.31	2.99E-06	1.21 (1.12-1.31)	0.35	0.30	5.78E-07	1.26 (1.15-1.39)	8.57E-12	1.23	0.5970
20q13.2	rs6126344	50407502	A/C	0.37	0.41	1.80E-05	0.87 (0.81-0.94)	0.38	0.42	2.33E-05	0.83 (0.76-0.90)	5.66E-08	0.85	0.4962
21q22.3	rs2018054	45703660	C/A	0.11	0.14	4.51E-05	0.77 (0.68-0.86)	0.13	0.13	8.53E-01	0.99 (0.87-1.12)	5.05E-04	0.86	0.0126
21q22.3	rs933150	45712588	A/G	0.18	0.21	3.77E-05	0.81 (0.73-0.89)	0.19	0.20	3.26E-01	0.95 (0.85-1.06)	1.06E-04	0.87	0.0684
22q11.21	rs7287092	19561423	A/G	0.19	0.16	9.64E-05	1.25 (1.13-1.37)	0.18	0.17	3.85E-01	1.05 (0.94-1.18)	1.16E-04	1.16	0.0532
22q11.21	rs2073764	19799410	A/G	0.01	0.04	2.61E-14	0.31 (0.23-0.42)	0.04	0.05	3.76E-01	0.91 (0.73-1.12)	2.20E-07	0.63	0.0000
22q12.1	rs8135014	28046561	A/G	0.14	0.11	6.51E-05	1.26 (1.12-1.41)	0.13	0.13	6.58E-01	1.03 (0.90-1.17)	9.97E-04	1.15	0.0468
22q12.2	rs36604	30340138	C/A	0.15	0.10	6.34E-07	1.61 (1.43-1.80)	0.13	0.14	1.66E-01	0.91 (0.80-1.04)	2.04E-07	1.25	0.0000
22q12.2	rs5763674	30386358	G/A	0.15	0.10	4.39E-07	1.61 (1.44-1.81)	0.13	0.14	1.76E-01	0.92 (0.81-1.04)	1.22E-07	1.26	0.0000
22q12.2	rs11089442	30689998	A/G	0.38	0.32	1.24E-07	1.33 (1.23-1.44)	0.35	0.36	9.45E-02	0.93 (0.85-1.01)	1.98E-05	1.14	0.0000
22q12.2	rs2839998	30729507	A/G	0.38	0.32	1.28E-07	1.33 (1.23-1.44)	0.35	0.36	8.09E-02	0.92 (0.84-1.01)	2.42E-05	1.14	0.0000
22q12.2	rs2072196	30975513	A/G	0.44	0.38	4.00E-06	1.30 (1.21-1.41)	0.41	0.43	7.46E-02	0.92 (0.85-1.01)	8.20E-05	1.12	0.0000
22q12.2	rs117353193	31010417	A/G	0.05	0.03	1.13E-05	1.92 (1.59-2.32)	0.04	0.05	1.34E-01	0.85 (0.68-1.05)	4.34E-05	1.35	0.0000
22q12.2	rs5753388	31310946	G/A	0.33	0.27	9.21E-06	1.35 (1.24-1.46)	0.29	0.31	8.73E-02	0.92 (0.84-1.01)	1.80E-05	1.14	0.0000
22q12.2	rs1003480	31346752	A/G	0.33	0.26	8.65E-06	1.34 (1.24-1.46)	0.29	0.31	8.46E-02	0.92 (0.84-1.01)	2.66E-05	1.14	0.0000
22q12.2	rs2073856	31663985	A/G	0.40	0.36	2.32E-06	1.19 (1.10-1.29)	0.37	0.36	7.81E-01	1.01 (0.93-1.11)	4.14E-04	1.11	0.0184
22q12.2	rs17220418	31697842	A/G	0.40	0.36	6.08E-06	1.18 (1.09-1.28)	0.37	0.36	6.82E-01	1.02 (0.93-1.11)	5.16E-04	1.11	0.0322
22q12.2	rs2006771	31998612	A/G	0.24	0.18	2.14E-05	1.36 (1.24-1.49)	0.23	0.22	3.42E-01	1.05 (0.95-1.16)	2.54E-08	1.21	0.0009
22q12.3	rs78212183	32213984	G/A	0.11	0.07	1.29E-06	1.60 (1.40-1.83)	0.10	0.09	3.29E-01	1.08 (0.93-1.25)	4.80E-09	1.34	0.0005
22q12.3	rs5756130	36684331	A/G	0.14	0.11	8.41E-05	1.34 (1.20-1.50)	0.14	0.13	3.13E-01	1.07 (0.94-1.21)	6.17E-06	1.21	0.0184

F_A: minor allele frequency in cases; F_U: minor allele frequency in controls; OR: odds ratio.

Alleles: shown as minor allele/major allele.

NSCLP Replication: replication study in 1,346 NSCLP cases and 4,542 controls of Chinese.

Meta analysis: Meta analysis of GWAS and NSCLP Replication.

The P value of GWAS and NSCLP Replication was calculated using logistic regression test

P(F) (The P value using Cochran-Mantel–Haenszel test) and OR(F) were calculated using fixed model.

*P het: P value for heterozygosity using logistic regression test and Bonferroni correction. P het> 0.05 was considered to signify no heterogeneity.

Supplementary Table 3. Association results of 14 novel loci and 3 suggestive loci in GWAS, NSCLP Replication and Meta analysis.

Loci	SNP	BP (hg19)	Alleles	GWAS				NSCLP Replication				Meta analysis		
				F_A	F_U	*P*	OR	F_A	F_U	*P*	OR	*P*(F)	OR(F)	P_{het}*
2p25.1	rs287980	9971366	G/A	0.23	0.27	1.99E-05	0.82 (0.75-0.89)	0.23	0.27	5.54E-04	0.84 (0.76-0.93)	1.94E-08	0.83	0.8120
2p25.1	rs287982	9972442	G/A	0.23	0.27	1.58E-05	0.82 (0.75-0.89)	0.23	0.27	2.28E-04	0.83 (0.75-0.92)	6.15E-09	0.82	0.8981
2q35	rs6734545	220653996	A/C	0.20	0.18	8.61E-05	1.16 (1.05-1.27)	0.21	0.17	4.61E-05	1.25 (1.13-1.40)	8.03E-07	1.20	0.3864
4p16.2	rs34246903	4794195	C/A	0.38	0.43	2.57E-05	0.82 (0.76-0.89)	0.40	0.43	1.09E-02	0.89 (0.82-0.97)	4.45E-08	0.85	0.2344
4p16.2	rs1907989	4818925	A/G	0.46	0.51	2.29E-06	0.81 (0.75-0.87)	0.48	0.50	1.94E-02	0.90 (0.83-0.98)	1.58E-08	0.85	0.1130
4q28.1	rs908822	124906257	A/G	0.10	0.07	6.13E-07	1.46 (1.28-1.66)	0.10	0.09	4.46E-02	1.16 (1.00-1.34)	4.33E-08	1.31	0.0545
5p12	rs10462065	44068846	A/C	0.24	0.20	3.51E-07	1.26 (1.15-1.38)	0.23	0.20	2.04E-03	1.18 (1.06-1.31)	1.12E-08	1.22	0.4835
5p12	rs4242107	44129083	A/G	0.44	0.40	6.08E-07	1.17 (1.09-1.26)	0.43	0.40	7.44E-03	1.13 (1.03-1.23)	1.32E-06	1.15	0.5979
6p24.3	rs9381107	9469238	A/G	0.31	0.37	1.97E-09	0.79 (0.73-0.86)	0.33	0.36	1.40E-02	0.89 (0.81-0.98)	2.72E-09	0.83	0.0900
8p11.23	rs13317	38269514	G/A	0.30	0.34	4.10E-06	0.83 (0.77-0.90)	0.32	0.35	7.43E-03	0.88 (0.80-0.97)	3.96E-08	0.85	0.4601
8q22.1	rs12681366	95401265	G/A	0.44	0.49	2.95E-06	0.82 (0.76-0.88)	0.44	0.48	2.48E-04	0.85 (0.78-0.93)	2.35E-10	0.83	0.5965
8q22.1	rs957448	95541302	G/A	0.44	0.50	3.78E-10	0.78 (0.72-0.84)	0.46	0.49	7.59E-04	0.86 (0.79-0.94)	9.60E-13	0.81	0.1260
8q22.2	rs11774066	99455311	A/G	0.42	0.38	8.65E-05	1.18 (1.09-1.28)	0.42	0.39	3.85E-03	1.14 (1.04-1.24)	3.39E-07	1.16	0.6166
8q22.2	rs1487022	100529826	A/C	0.44	0.39	5.64E-05	1.19 (1.10-1.28)	0.44	0.41	1.43E-03	1.15 (1.06-1.26)	7.84E-08	1.17	0.7214
9q22.32	rs10512248	98259703	C/A	0.29	0.34	1.80E-07	0.79 (0.73-0.86)	0.30	0.33	3.19E-03	0.87 (0.79-0.95)	5.10E-10	0.82	0.2026
9q22.32	rs28361526	98295863	A/G	0.30	0.34	1.65E-05	0.83 (0.77-0.90)	0.31	0.34	3.02E-02	0.90 (0.82-0.99)	1.95E-06	0.86	0.2949
12q13.13	rs3741442	53346750	G/A	0.46	0.41	1.17E-06	1.22 (1.13-1.32)	0.46	0.41	3.36E-06	1.23 (1.13-1.34)	3.72E-12	1.22	0.9598
12q13.2	rs705704	56435412	A/G	0.27	0.23	4.39E-05	1.22 (1.12-1.33)	0.28	0.24	5.97E-05	1.22 (1.11-1.34)	1.29E-09	1.22	0.9839
12q21.1	rs2304269	72080272	G/A	0.38	0.44	1.07E-07	0.79 (0.73-0.85)	0.41	0.45	1.12E-04	0.84 (0.77-0.92)	1.32E-12	0.81	0.3903
12q21.1	rs7967428	72089040	G/A	0.38	0.44	1.63E-07	0.79 (0.73-0.85)	0.41	0.45	1.57E-04	0.84 (0.77-0.92)	3.08E-12	0.81	0.3871
14q22.1	rs7148069	51839645	A/G	0.23	0.19	1.94E-05	1.27 (1.16-1.40)	0.22	0.20	7.21E-03	1.16 (1.04-1.28)	1.69E-08	1.22	0.2538
14q32.13	rs1243572	95379499	G/A	0.48	0.42	1.37E-07	1.26 (1.17-1.36)	0.45	0.42	6.09E-03	1.13 (1.04-1.23)	3.52E-10	1.20	0.1138

14q32.13	rs1243573	95379583	C/A	0.48	0.42	2.83E-07	1.25 (1.16-1.35)	0.45	0.42	7.30E-03	1.13 (1.03-1.23)	8.61E-10	1.20	0.1178
17q21.32	rs4968247	44988703	A/G	0.37	0.41	4.11E-06	0.84 (0.78-0.91)	0.37	0.41	2.43E-05	0.83 (0.76-0.90)	8.70E-10	0.83	0.8605
17q21.32	rs1838105	45008935	A/G	0.45	0.39	1.84E-07	1.26 (1.17-1.36)	0.42	0.38	3.48E-04	1.17 (1.08-1.28)	1.31E-11	1.22	0.3543
20q13.2	rs6126344	50407502	A/C	0.37	0.41	1.80E-05	0.87 (0.81-0.94)	0.38	0.42	2.33E-05	0.83 (0.76-0.90)	5.66E-08	0.85	0.4962

F_A: minor allele frequency in cases; F_U: minor allele frequency in controls; OR: odds ratio.

Alleles: shown as minor allele/major allele.

P(F) and OR(F) were calculated using fixed model.

NSCLP Replication: replication study in 1,346 NSCLP cases and 4,542 controls of Chinese.

Meta analysis: Meta analysis of GWAS and NSCLP Replication.

The P value of GWAS and NSCLP Replication were calculated using logistic regression test

P(F) (The P value using Cochran-Mantel–Haenszel test) and OR(F) were calculated using fixed model.

*P het: P value for heterozygosity using logistic regression test Bonferroni correction. P het> 0.05 was considered to signify no heterogeneity.

Supplementary Table 4. Association results for 22 previously reported NSCL/P loci in GWAS, NSCLP Replication and Meta analysis.

Loci	SNP	BP (hg19)	Gene	Alleles	GWAS				NSCLP Replication				Meta analysis		
					F_A	F_U	*P*	OR	F_A	F_U	*P*	OR	*P*(F)	OR(F)	P_{het}*
1p36.13	rs742071	18979874	*PAX7*	A/C	0.06	0.04	4.73E-05	1.47 (1.24-1.74)	0.05	0.04	9.17E-03	1.30 (1.07-1.59)	4.26E-07	1.40	0.4859
1p22.1	rs4147827	94548080	*ABCA4*	C/G	0.08	0.05	4.86E-05	1.41 (1.21-1.64)	0.08	0.06	3.98E-03	1.28 (1.08-1.51)	1.89E-07	1.35	0.5255
1p22.1	rs17461953	94551450	*ABCA4*	C/A	0.08	0.05	2.68E-05	1.42 (1.22-1.65)	0.08	0.06	3.01E-03	1.29 (1.09-1.52)	7.04E-08	1.36	0.5006
1p22.1	rs481931	94570016	*ABCA4*	A/C	0.33	0.37	4.82E-06	0.83 (0.77-0.90)	0.33	0.39	2.79E-08	0.77 (0.71-0.85)	1.06E-12	0.80	0.3687
1p22.1	rs4147803	94582293	*ABCA4*	G/C	0.29	0.33	1.22E-06	0.81 (0.75-0.88)	0.29	0.34	2.27E-06	0.80 (0.73-0.88)	7.97E-12	0.81	0.8369
1q32.2	rs861020	209977111	*IRF6*	A/G	0.24	0.19	6.38E-12	1.34 (1.23-1.47)	0.23	0.19	6.63E-06	1.27 (1.15-1.41)	1.30E-14	1.31	0.5428
1q32.2	rs642961	209989270	*IRF6*	A/G	0.24	0.19	3.86E-12	1.35 (1.23-1.48)	0.23	0.19	2.23E-06	1.29 (1.16-1.43)	2.76E-15	1.32	0.6061
1q32.2	rs2064163	210048819	*IRF6*	A/C	0.38	0.44	1.04E-09	0.77 (0.71-0.83)	0.38	0.44	8.18E-09	0.77 (0.71-0.84)	8.60E-19	0.77	0.9625
1q32.2	rs9430019	210050794	*IRF6*	A/G	0.31	0.26	1.77E-07	1.27 (1.17-1.38)	0.30	0.26	2.58E-05	1.23 (1.12-1.35)	1.68E-12	1.25	0.6420
2p24.2	rs10172734	16733054	*FAM49A*	G/A	0.26	0.32	3.85E-13	0.76 (0.70-0.83)	0.26	0.33	1.58E-11	0.72 (0.65-0.79)	2.89E-20	0.74	0.4992
2p24.2	rs7552	16733928	*FAM49A*	A/G	0.24	0.30	1.57E-14	0.74 (0.68-0.81)	0.24	0.31	5.64E-12	0.71 (0.64-0.78)	5.83E-22	0.73	0.5814
2p21	rs7590268	43540125	*THADA*	C/A	0.07	0.05	3.02E-04	1.33 (1.14-1.56)	NA	NA	NA	NA	NA	NA	NA
2p21	rs6544647	43578419	*THADA*	A/G	0.08	0.06	2.33E-05	1.37 (1.18-1.58)	NA	NA	NA	NA	NA	NA	NA
3p11.1	rs10511145	89597679	*EPHA3*	A/C	0.17	0.20	1.53E-04	0.83 (0.75-0.91)	NA	NA	NA	NA	NA	NA	NA
3q12.1	rs1038294	99503728	*COL8A1*	A/G	0.13	0.17	1.18E-07	0.73 (0.66-0.81)	0.15	0.16	2.12E-01	0.93 (0.82-1.05)	4.47E-07	0.81	0.0127
3q12.1	rs1688766	99647850	*COL8A1*	A/G	0.13	0.17	1.32E-07	0.73 (0.65-0.81)	0.15	0.16	2.28E-01	0.93 (0.82-1.05)	4.83E-07	0.81	0.0113
8q21.3	rs12543318	88868340	*DCAF4L2*	A/C	0.32	0.38	2.43E-07	0.78 (0.72-0.84)	0.33	0.37	7.83E-04	0.86 (0.78-0.94)	8.80E-12	0.81	0.2050
8q21.3	rs1034832	88918331	*DCAF4L2*	C/A	0.31	0.36	5.92E-07	0.79 (0.73-0.85)	0.32	0.35	1.65E-03	0.86 (0.79-0.95)	1.35E-10	0.82	0.2243
8q24.21	rs7845615	129888794	*MYC*	A/G	0.22	0.18	5.11E-06	1.28 (1.17-1.41)	0.20	0.17	8.59E-05	1.25 (1.12-1.39)	1.03E-10	1.27	0.8110
8q24.21	rs7017252	129950844	*MYC*	A/G	0.08	0.05	3.27E-06	1.59 (1.36-1.85)	0.07	0.05	4.18E-08	1.62 (1.36-1.93)	8.47E-16	1.60	0.8960
9q22.2	rs7871395	92209587	*GADD45G*	A/G	0.30	0.26	4.54E-05	1.23 (1.13-1.34)	0.29	0.26	1.07E-03	1.17 (1.07-1.29)	6.06E-09	1.21	0.5782
9q22.33	rs7043516	100617433	*FOXE1*	C/A	0.04	0.06	1.68E-04	0.70 (0.59-0.85)	NA	NA	NA	NA	NA	NA	NA

10q25.3	rs6585429	118893231	*VAX1*	G/A	0.39	0.44	4.92E-08	0.81 (0.75-0.87)	0.39	0.45	2.22E-06	0.81 (0.74-0.88)	7.14E-13	0.81	0.9967
13q31.1	rs9545308	80639405	*SPRY2*	A/C	0.15	0.12	4.09E-05	1.30 (1.17-1.45)	0.14	0.11	2.34E-04	1.27 (1.12-1.44)	2.00E-09	1.29	0.8103
15q13.3	rs1258763	33050423	*GREM1*	A/G	0.08	0.07	2.21E-02	1.18 (1.02-1.37)	NA	NA	NA	NA	NA	NA	NA
15q22.2	rs78512626	61432877	*TPM1*	C/A	0.12	0.15	8.05E-06	0.76 (0.67-0.85)	0.14	0.15	3.84E-01	0.95 (0.84-1.07)	7.02E-05	0.84	0.0261
16p13.3	rs2283487	3969886	*CREBBP / ADCY9*	G/A	0.42	0.47	3.18E-06	0.83 (0.76-0.89)	0.43	0.47	3.91E-05	0.83 (0.76-0.91)	1.27E-10	0.83	0.9121
16p13.3	rs17136624	3996282	*CREBBP / ADCY9*	A/G	0.25	0.22	8.43E-05	1.21 (1.11-1.32)	0.27	0.23	3.11E-06	1.28 (1.15-1.42)	3.82E-10	1.24	0.5269
17p13.1	rs2872615	8914693	*NTN1*	G/A	0.43	0.48	7.45E-06	0.80 (0.74-0.87)	0.43	0.47	1.06E-04	0.84 (0.77-0.92)	8.81E-12	0.82	0.5224
17p13.1	rs1880646	8929845	*NTN1*	A/G	0.52	0.46	4.98E-06	1.25 (1.16-1.35)	0.52	0.48	2.60E-04	1.17 (1.08-1.28)	1.69E-11	1.22	0.4104
17q22	rs12951993	54760614	*NOG*	A/G	0.36	0.32	1.19E-05	1.21 (1.12-1.31)	0.36	0.33	2.88E-02	1.11 (1.01-1.21)	5.26E-07	1.16	0.2119
17q22	rs227731	54773238	*NOG*	C/A	0.36	0.32	1.74E-05	1.22 (1.12-1.32)	0.36	0.32	1.19E-03	1.16 (1.06-1.27)	8.83E-09	1.19	0.5623
17q23.2	rs16946713	61087073	*TANC2 / DCAF7*	G/A	0.07	0.06	5.94E-02	1.16 (0.99-1.35)	NA	NA	NA	NA	NA	NA	NA
17q25.3	rs2273280	76103663	*RBFOX3*	A/G	0.15	0.18	5.05E-05	0.79 (0.71-0.88)	NA	NA	NA	NA	NA	NA	NA
17q25.3	rs76579845	76598716	*RBFOX3*	A/C	0.02	0.04	2.08E-07	0.51 (0.40-0.65)	NA	NA	NA	NA	NA	NA	NA
19q13.11	rs10404307	33512011	*RHPN2*	C/A	0.15	0.18	8.99E-06	0.79 (0.71-0.88)	NA	NA	NA	NA	NA	NA	NA
20q12	rs6129653	39275603	*MAFB*	A/G	0.35	0.31	2.99E-06	1.21 (1.12-1.31)	0.35	0.30	5.78E-07	1.26 (1.15-1.39)	8.57E-12	1.23	0.5970

F_A: minor allele frequency in cases; F_U: minor allele frequency in controls; OR: odds ratio.

Alleles: shown as minor allele/major allele.

NSCLP Replication: replication study in 1,346 NSCLP cases and 4,542 controls of Chinese.

Meta analysis: Meta analysis of GWAS and NSCLP Replication.

The P value of GWAS and NSCLP Replication using using logistic regression test

P(F) (The P value using Cochran-Mantel–Haenszel test) and OR(F) were calculated using fixed model.

*P het: P value for heterozygosity using logistic regression test Bonferroni correction. P het> 0.05 was considered to signify no heterogeneity.

Supplementary Table 5. Independent Secondary signals observed in the discovery and NSCLP Replication samples when performing conditional on one previously reported loci.

Loci	Gene	SNP	Alleles	Combined Discovery and Replication 1					Logistic condition			LD	
				MAF		***P***	**OR**	**95% CI**	**Condition**	***P***	**OR**	**D'**	r^2
				**Cases**	**Controls**								
1q32.2	*IRF6*	rs861020	A/G	0.23	0.19	1.30E-14	1.31	(1.23-1.41)	rs642961	NA	NA	0.99	0.98
									rs2064163	1.64E-06	1.20	0.82	0.12
									rs9430019	8.79E-17	1.34	0.24	0.01
	IRF6/DIEXF	rs642961	A/G	0.23	0.19	2.76E-15	1.32	(1.23-1.41)	rs861020	NA	NA	0.99	0.98
									rs2064163	9.80E-07	1.20	0.82	0.12
									rs9430019	1.81E-17	1.35	0.25	0.01
	DIEXF/SYT14	rs2064163	A/C	0.38	0.44	8.60E-19	0.77	(0.73-0.82)	rs861020	6.43E-11	0.81	0.82	0.12
									rs642961	1.05E-10	0.82	0.82	0.12
									rs9430019	1.43E-09	0.81	1.00	0.28
	DIEXF/SYT14	rs9430019	A/G	0.31	0.26	1.68E-12	1.25	(1.18-1.34)	rs861020	5.64E-15	1.28	0.24	0.01
									rs642961	1.06E-14	1.28	0.25	0.01
									rs2064163	4.51E-03	1.11	1.00	0.28

MAF: minor allele frequency; OR: odds ratio; 95%CI: 95% confidence interval; LD: linkage disequilibrium; D': D prime; r2: r square.

Alleles: shown as minor allele/major allele.

P value using stepwise subset-based conditional logistic regression

Supplementary Table 6. Association results for 40 SNPs in NSCLO Replication.

Loci	SNP	BP (hg19)	Alleles	NSCLO Replication				New/Reported
				F_A	F_U	*P*	OR	
2p25.1	rs287980	9971366	G/A	0.25	0.26	2.34E-01	0.93 (0.84-1.05)	New
2p25.1	rs287982	9972442	G/A	0.25	0.26	1.65E-01	0.92 (0.83-1.03)	New
4p16.2	rs34246903	4794195	C/A	0.40	0.42	2.91E-01	0.95 (0.86-1.05)	New
4p16.2	rs1907989	4818925	A/G	0.48	0.50	6.42E-02	0.91 (0.83-1.01)	New
4q28.1	rs908822	124906257	A/G	0.09	0.08	7.64E-02	1.17 (0.98-1.38)	New
5p12	rs10462065	44068846	A/C	0.24	0.22	7.92E-02	1.11 (0.99-1.24)	New
6p24.3	rs9381107	9469238	A/G	0.32	0.35	4.79E-02	0.90 (0.81-1.00)	New
8p11.23	rs13317	38269514	G/A	0.31	0.33	7.59E-02	0.91 (0.82-1.01)	New
8q22.1	rs12681366	95401265	G/A	0.46	0.48	6.16E-02	0.91 (0.83-1.01)	New
8q22.1	rs957448	95541302	G/A	0.47	0.49	5.72E-02	0.91 (0.83-1.00)	New
9q22.32	rs10512248	98259703	C/A	0.33	0.35	4.47E-02	0.90 (0.81-1.00)	New
12q13.13	rs3741442	53346750	G/A	0.44	0.42	6.42E-02	1.10 (0.99-1.21)	New
12q13.2	rs705704	56435412	A/G	0.24	0.25	2.84E-01	0.94 (0.84-1.05)	New
12q21.1	rs2304269	72080272	G/A	0.41	0.44	2.04E-02	0.89 (0.81-0.98)	New
12q21.1	rs7967428	72089040	G/A	0.41	0.44	6.56E-02	0.91 (0.83-1.01)	New
14q22.1	rs7148069	51839645	A/G	0.21	0.20	6.21E-01	1.03 (0.91-1.16)	New
14q32.13	rs1243572	95379499	G/A	0.47	0.43	**7.75E-04**	1.18 (1.07-1.30)	New
14q32.13	rs1243573	95379583	C/A	0.46	0.43	8.19E-03	1.14 (1.03-1.26)	New
17q21.32	rs4968247	44988703	A/G	0.37	0.40	1.44E-03	0.85 (0.77-0.94)	New
17q21.32	rs1838105	45008935	A/G	0.43	0.38	**5.98E-04**	1.19 (1.08-1.31)	New
1p22.1	rs4147803	94582293	G/C	0.30	0.34	**9.89E-04**	0.84 (0.75-0.93)	Reported
1q32.2	rs861020	209977111	A/G	0.30	0.18	**1.02E-28**	1.86 (1.67-2.08)	Reported

1q32.2	rs642961	209989270	A/G	0.30	0.18	**1.69E-30**	1.90 (1.70-2.13)	Reported
1q32.2	rs2064163	210048819	A/C	0.39	0.45	**1.06E-06**	0.78 (0.71-0.86)	Reported
1q32.2	rs9430019	210050794	A/G	0.30	0.26	**2.28E-04**	1.22 (1.10-1.36)	Reported
2p24.2	rs10172734	16733054	G/A	0.25	0.34	**1.69E-14**	0.65 (0.58-0.73)	Reported
2p24.2	rs7552	16733928	A/G	0.26	0.32	**1.62E-07**	0.75 (0.67-0.83)	Reported
8q21.3	rs12543318	88868340	A/C	0.31	0.37	**1.84E-07**	0.76 (0.68-0.84)	Reported
8q21.3	rs1034832	88918331	C/A	0.28	0.34	**1.05E-07**	0.75 (0.67-0.83)	Reported
8q24.21	rs7845615	129888794	A/G	0.21	0.18	8.40E-03	1.18 (1.04-1.33)	Reported
8q24.21	rs7017252	129950844	A/G	0.07	0.05	2.88E-03	1.36 (1.11-1.66)	Reported
9q22.2	rs7871395	92209587	A/G	0.31	0.27	**1.22E-04**	1.23 (1.11-1.37)	Reported
10q25.3	rs6585429	118893231	G/A	0.38	0.44	**8.42E-07**	0.78 (0.71-0.86)	Reported
13q31.1	rs9545308	80639405	A/C	0.14	0.12	4.95E-03	1.23 (1.06-1.42)	Reported
16p13.3	rs2283487	3969886	G/A	0.45	0.47	1.47E-01	0.93 (0.84-1.03)	Reported
16p13.3	rs17136624	3996282	A/G	0.24	0.22	1.54E-01	1.09 (0.97-1.22)	Reported
17p13.1	rs2872615	8914693	G/A	0.43	0.47	**8.21E-04**	0.85 (0.77-0.93)	Reported
17p13.1	rs1880646	8929845	A/G	0.51	0.49	6.30E-02	1.10 (1.00-1.21)	Reported
17q22	rs227731	54773238	C/A	0.36	0.34	2.98E-02	1.12 (1.01-1.24)	Reported
20q12	rs6129653	39275603	A/G	0.34	0.28	**2.34E-06**	1.28 (1.16-1.42)	Reported

F_A: minor allele frequency in cases; F_U: minor allele frequency in controls; OR: odds ratio.

Alleles: shown as minor allele/major allele.

NSCLO Replication: replication study in 1,104 NSCLO cases and 3,312 controls of Chinese.

P value using logistic regression test

Supplementary Table 7. Association results of 40 SNPs in NSCPO Replication.

Loci	SNP	BP (hg19)	Alleles	NSCPO Replication				New/Reported
				F_A	F_U	*P*	OR	
2p25.1	rs287980	9971366	G/A	0.27	0.26	7.87E-01	1.02 (0.91-1.13)	New
2p25.1	rs287982	9972442	C/T	0.27	0.26	7.63E-01	1.02 (0.91-1.14)	New
4p16.2	rs34246903	4794195	C/A	0.41	0.42	7.77E-01	0.99 (0.89-1.09)	New
4p16.2	rs1907989	4818925	G/A	0.50	0.50	5.78E-01	1.03 (0.93-1.13)	New
4q28.1	rs908822	124906257	T/C	0.09	0.08	5.21E-01	1.06 (0.89-1.26)	New
5p12	rs10462065	44068846	A/C	0.22	0.22	7.17E-01	0.98 (0.87-1.10)	New
6p24.3	rs9381107	9469238	A/G	0.34	0.35	2.81E-01	0.95 (0.85-1.05)	New
8p11.23	rs13317	38269514	C/T	0.32	0.33	3.83E-01	0.95 (0.86-1.06)	New
8q22.1	rs12681366	95401265	C/T	0.47	0.48	4.01E-01	0.96 (0.87-1.06)	New
8q22.1	rs957448	95541302	G/A	0.48	0.49	1.33E-01	0.93 (0.84-1.02)	New
9q22.32	rs10512248	98259703	G/T	0.31	0.35	**1.23E-04**	0.82 (0.73-0.91)	New
12q13.13	rs3741442	53346750	C/T	0.44	0.42	9.72E-02	1.09 (0.99-1.20)	New
12q13.2	rs705704	56435412	A/G	0.25	0.25	1.00E+00	1.00 (0.89-1.12)	New
12q21.1	rs2304269	72080272	C/T	0.45	0.44	3.03E-01	1.05 (0.95-1.16)	New
12q21.1	rs7967428	72089040	G/A	0.45	0.44	1.72E-01	1.07 (0.97-1.18)	New
14q22.1	rs7148069	51839645	T/C	0.19	0.20	4.90E-01	0.96 (0.85-1.08)	New
14q32.13	rs1243572	95379499	C/T	0.44	0.43	2.14E-01	1.06 (0.96-1.17)	New
14q32.13	rs1243573	95379583	C/A	0.45	0.43	8.60E-02	1.09 (0.99-1.20)	New
17q21.32	rs4968247	44988703	T/C	0.40	0.40	4.34E-01	0.96 (0.87-1.06)	New
17q21.32	rs1838105	45008935	A/G	0.38	0.38	9.30E-01	1.00 (0.90-1.10)	New
1p22.1	rs4147803	94582293	C/G	0.32	0.34	2.72E-01	0.94 (0.85-1.05)	Reported
1q32.2	rs861020	209977111	A/G	0.17	0.18	6.63E-02	0.89 (0.78-1.01)	Reported

1q32.2	rs642961	209989270	A/G	0.17	0.18	1.16E-01	0.90 (0.79-1.03)	Reported
1q32.2	rs2064163	210048819	T/G	0.44	0.45	8.25E-01	0.99 (0.90-1.09)	Reported
1q32.2	rs9430019	210050794	T/C	0.19	0.26	**1.58E-11**	0.66 (0.59-0.75)	Reported
2p24.2	rs10172734	16733054	G/A	0.33	0.34	4.36E-01	0.96 (0.87-1.06)	Reported
2p24.2	rs7552	16733928	A/G	0.28	0.32	1.81E-03	0.84 (0.76-0.94)	Reported
8q21.3	rs12543318	88868340	A/C	0.35	0.37	1.18E-01	0.92 (0.83-1.02)	Reported
8q21.3	rs1034832	88918331	G/T	0.35	0.34	5.45E-01	1.03 (0.93-1.14)	Reported
8q24.21	rs7845615	129888794	T/C	0.19	0.18	3.06E-01	1.07 (0.94-1.21)	Reported
8q24.21	rs7017252	129950844	T/C	0.07	0.05	**4.49E-04**	1.43 (1.17-1.74)	Reported
9q22.2	rs7871395	92209587	T/C	0.27	0.27	6.28E-01	1.03 (0.92-1.15)	Reported
10q25.3	rs6585429	118893231	G/A	0.43	0.44	2.96E-01	0.95 (0.86-1.05)	Reported
13q31.1	rs9545308	80639405	T/G	0.14	0.12	7.49E-03	1.22 (1.05-1.40)	Reported
16p13.3	rs2283487	3969886	G/A	0.47	0.47	9.49E-01	1.00 (0.90-1.10)	Reported
16p13.3	rs17136624	3996282	A/G	0.22	0.22	6.91E-01	1.02 (0.91-1.15)	Reported
17p13.1	rs2872615	8914693	C/T	0.49	0.47	5.82E-02	1.10 (1.00-1.21)	Reported
17p13.1	rs1880646	8929845	A/G	0.47	0.49	2.52E-01	0.94 (0.86-1.04)	Reported
17q22	rs227731	54773238	G/T	0.33	0.34	4.40E-01	0.96 (0.87-1.06)	Reported
20q12	rs6129653	39275603	T/C	0.31	0.28	8.99E-03	1.15 (1.04-1.28)	Reported

F_A: minor allele frequency in cases; F_U: minor allele frequency in controls; OR: odds ratio.

Alleles: shown as minor allele/major allele.

NSCPO Replication: replication study in 1,104 NSCPO cases and 3,312 controls of Chinese.

The P value using logistic regression test;

Supplementary Table 8. Genotype-phenotype analysis.

	Loci	SNP	BP (hg19)	Allele	F_A	F_U	F-Healthy*	*P*	OR[#]	Gene
Gender (NSCLP)	1q32.2	rs861020	209977111	**A**/G	0.25	0.20	0.19	**2.94E-04**	1.27 (1.12-1.45)	*IRF6*
		rs642961	209989270	**A**/G	0.25	0.20	0.19	**3.47E-04**	1.27 (1.11-1.44)	*IRF6*
		rs2064163	210048819	A/**C**	0.36	0.41	0.44	**1.38E-04**	1.23 (1.11-1.37)	*IRF6*
		rs9430019	210050794	**A**/G	0.32	0.28	0.26	1.26E-03	1.21 (1.08-1.36)	*IRF6*
Mother's Age (NSCLP)	8q21.3	rs12543318	88868340	A/**C**	0.27	0.34	0.38	**3.29E-04**	1.43 (1.18-1.72)	*MMP16*
		rs1034832	88918331	C/**A**	0.26	0.32	0.36	2.98E-03	1.35 (1.11-1.64)	*MMP16*

NSCLP: non-syndromic cleft lip with palate; OR: odds ratio.

F_A: minor allele frequency of male or older mothers (> 35y) in NSCLP, respectively.

F_U: minor allele frequency of female or standard age mothers in the reference group (25 - 35y) in NSCLP, respectively.

Alleles: shown as minor allele/major allele, and risk allele was in bold.

[#]OR was calculated based on risk allele.

*Minor allele frequency in controls from discovery GWAS stage.

The P value using logistic regression test;

Supplementary Table 9. Association results for 24 SNPs in foreign replication data sets.

Loci	SNP	BP	Central European replication					Asian case-parent trios for replication								European ancestry case-parent trios for replication								New /Reported
			**Alleles**	**F_A**	**F_U**	**OR**	***P***	**MAF**	**Beta coef**	**SE**	**Test stat**	**RR**	**Trios**	***P***	**Missing**	**MAF**	**Beta coef**	**SE**	**Test stat**	**RR**	**Trios**	***P***	**Missing**	
2p25.1	rs287980	9971366	G/A	0.20	0.21	0.94	5.34E-01	0.26	-0.06	0.08	0.54	0.94	544	4.63E-01	0.00	0.21	-0.03	0.10	0.09	0.97	324	7.62E-01	0.00	New
2p25.1	rs287982	9972442	C/T	0.20	0.21	0.94	5.21E-01	0.26	-0.06	0.08	0.54	0.94	544	4.63E-01	0.00	0.21	-0.04	0.10	0.16	0.96	321	6.85E-01	0.00	New
4p16.2	rs34246903	4794195	A/C	0.70	0.66	1.22	**2.39E-02**	0.42	-0.16	0.07	4.99	0.85	604	**2.54E-02**	0.02	0.33	0.12	0.09	1.92	1.13	397	1.65E-01	0.01	New
4p16.2	rs1907989	4818925	A/G	0.57	0.61	0.83	**1.92E-02**	0.49	0.20	0.07	8.33	1.22	646	**3.91E-03**	0.00	0.36	0.06	0.09	0.43	1.06	423	5.14E-01	0.00	New
4q28.1	rs908822	124906257	C/T	0.92	0.93	0.83	2.44E-01	0.08	0.34	0.13	7.24	1.41	230	**7.14E-03**	0.00	0.07	0.14	0.16	0.77	1.15	144	3.80E-01	0.00	New
5p12	rs10462065	44068846	C/A	0.89	0.89	1.05	7.28E-01	0.24	0.18	0.08	4.71	1.19	510	**3.01E-02**	0.01	0.12	0.05	0.12	0.19	1.05	237	6.66E-01	0.00	New
6p24.3	rs9381107	9469238	G/A	0.84	0.82	1.18	1.43E-01	0.32	0.02	0.08	0.04	1.02	503	8.43E-01	0.05	0.15	-0.03	0.11	0.08	0.97	271	7.75E-01	0.00	New
8p11.23	rs13317	38269514	T/C	0.79	0.78	1.08	4.46E-01	0.31	-0.08	0.07	1.24	0.92	582	2.65E-01	0.00	0.23	-0.28	0.09	8.70	0.76	377	**3.17E-03**	0.00	New
8q22.1	rs12681366	95401265	T/C	0.68	0.66	1.09	3.01E-01	0.45	-0.04	0.07	0.34	0.96	639	5.62E-01	0.00	0.31	-0.11	0.09	1.45	0.90	381	2.28E-01	0.00	New
8q22.1	rs957448	95541302	A/G	0.77	0.74	1.14	1.49E-01	0.47	-0.11	0.07	2.44	0.90	647	1.18E-01	0.00	0.23	-0.08	0.10	0.69	0.92	348	4.07E-01	0.00	New
9q22.32	rs10512248	98259703	T/G	0.66	0.67	0.93	4.34E-01	0.28	-0.21	0.09	6.03	0.81	441	**1.41E-02**	0.09	0.32	-0.15	0.09	2.71	0.86	381	1.00E-01	0.02	New
12q13.13	rs3741442	53346750	C/T	0.99	1.00	0.72	5.42E-01	0.54	-0.26	0.07	14.64	0.77	630	**1.30E-04**	0.01	0.02	-0.73	0.33	5.01	0.48	38	**2.52E-02**	0.00	New
12q13.2	rs705704	56435412	G/A	0.66	0.69	0.86	7.44E-02	0.23	0.03	0.08	0.19	1.04	516	6.61E-01	0.00	0.35	0.28	0.09	10.58	1.32	439	**1.15E-03**	0.00	New
12q21.1	rs2304269	72080272	T/C	0.95	0.95	1.05	7.81E-01	0.43	-0.16	0.07	4.92	0.85	564	**2.66E-02**	0.04	0.07	-0.08	0.17	0.25	0.92	135	6.17E-01	0.00	New
12q21.1	rs7967428	72089040	A/G	0.95	0.95	1.00	9.91E-01	0.43	-0.16	0.07	5.15	0.85	630	**2.32E-02**	0.00	0.07	-0.07	0.17	0.17	0.93	136	6.78E-01	0.00	New
14q22.1	rs7148069	51839645	C/T	0.68	0.67	1.02	7.92E-01	0.17	0.02	0.10	0.02	1.02	306	8.75E-01	0.07	0.31	0.10	0.10	1.05	1.11	336	3.06E-01	0.04	New
14q32.13	rs1243572	95379499	T/C	0.21	0.22	0.91	3.65E-01	0.57	-0.02	0.07	0.07	0.98	656	7.84E-01	0.00	0.23	-0.07	0.10	0.46	0.94	355	4.99E-01	0.00	New
14q32.13	rs1243573	95379583	A/C	0.20	0.22	0.92	4.10E-01	0.57	-0.02	0.07	0.07	0.98	656	7.84E-01	0.00	0.23	-0.07	0.10	0.46	0.94	355	4.99E-01	0.00	New
17q21.32	rs4968247	44988703	C/T	0.34	0.33	1.07	4.81E-01	0.61	0.23	0.08	9.13	1.26	528	**2.52E-03**	0.05	0.33	0.14	0.09	2.40	1.15	372	1.21E-01	0.03	New

17q21.32	rs1838105	45008935	A/G	0.39	0.33	1.27	**5.83E-03**	0.40	0.17	0.07	6.00	1.19	625	**1.43E-02**	0.00	0.38	0.12	0.08	2.22	1.13	449	1.37E-01	0.00	New
2p24.2	rs10172734	16733054	G/A	0.68	0.72	0.82	**2.42E-02**	0.29	-0.14	0.08	3.59	0.87	555	5.83E-02	0.01	0.66	-0.20	0.09	5.49	0.82	423	**1.91E-02**	0.00	Reported
2p24.2	rs7552	16733928	A/G	0.68	0.72	0.82	**2.52E-02**	0.27	-0.15	0.08	3.55	0.86	546	5.95E-02	0.01	0.66	-0.20	0.09	5.28	0.82	424	**2.15E-02**	0.00	Reported
16p13.3	rs2283487	3969886	A/G	0.38	0.35	1.13	1.49E-01	0.55	0.10	0.07	1.95	1.10	614	1.62E-01	0.01	0.36	0.22	0.08	6.92	1.25	440	**8.51E-03**	0.00	Reported
16p13.3	rs17136624	3996282	G/A	0.70	0.73	0.87	1.19E-01	0.22	0.08	0.08	0.83	1.08	480	3.61E-01	0.00	0.28	0.09	0.09	0.90	1.09	397	3.43E-01	0.00	Reported

Central European Replication: replication study in 399 NSCL/P cases and 1,318 controls of Central European origin.

Asian case-parent trios for replication: replication study in 861 NSCL/P case-parent trios of Asian ancestry (including: Chinese, Singaporean, Taiwanese, Korean and Filipino).

European ancestry case-parent trios for replication: replication study in 557 NSCL/P case-parent trios of European ancestry.

The P value using logistic regression test;

Supplementary Table 10. Comparisons of the allele frequencies for 9 previously reported SNPs obtained from the 22 reported loci among different populations based on GWAS data.

**Loci**	Index SNPs	Alleles	MAF_cases	MAF_controls	Population	Types
1p22.1	rs481931	A	0.339	/	Asian & European	trios
			0.351	/	European	trios
			0.332	/	Asian	trios
		A/C	0.33	0.37	Chinese Han	case-control
	rs560426	G	0.399	/	Asian & European	trios
			0.471	/	European	trios
			0.342	/	Asian	trios
		G/A	0.32	0.3	Chinese Han	case-control
1q32.2	rs861020	A	0.245	/	Asian & European	trios
			0.246	/	European	trios
			0.244	/	Asian	trios
		A/G	0.24	0.19	Chinese Han	case-control
	rs642961	A/G	0.26	/	Norway	trios
			0.24	/	Denmark	trios
			0.25	/	EUROCRAN	trios
			0.24	/	Europe	trios
			0.32	/	Philippines	trios
		A/G	0.24	0.19	Chinese Han	case-control

8q24.21	**rs987525**	**A/C**	**0.381(A)**	**0.202(A)**	Central European	case-control
		**A/C**	**0.07**	**0.07**	Chinese Han	case-control
	**rs1530300**	**C/T**	**0.478(C)**	**0.301(C)**	Central European	case-control
		**G/A**	**0.05**	**0.04**	Chinese Han	case-control
	**rs7017252**	**T/C**	**0.449(C)**	**0.393(T)**	Central European	case-control
		**A/G**	**0.08**	**0.05**	Chinese Han	case-control
15q13.3	rs1258763	G/A	0.252 (nsCLP_hard+soft)	/	European, Asian, Central European, Mexican, Yemeni	Meta (including trios)
			0.119 (nsCLP_soft)	/	European, Asian, Central European, Mexican, Yemeni	Meta (including trios)
		A/G	0.08	0.07	Chinese Han	case-control
17q22	rs227731	C/A	0.496 (A)	0.416 (C)	Central European	case-control
		C/A	0.36	0.32	Chinese Han	case-control

Supplementary Table 11. Expression results for candidate genes identified in GWAS on mouse embryo drawn from EMAGE database.

Gene	Chr.	Symbol	ID	Structures	Level	Theiler Stage	Stage Given	EMAGE ID
**branchial arch**								
IRF6	1	*Irf6*	MGI:1859211	1st branchial arch mandibular component	weak	TS17	10.5 dpc	EMAGE:26246
IRF6	1	*Irf6*	MGI:1859211	1st branchial arch maxillary component	weak	TS17	10.5 dpc	EMAGE:26246
IRF6	1	*Irf6*	MGI:1859211	2nd branchial arch	weak	TS17	10.5 dpc	EMAGE:26246
MSX1	4	*Msx1*	MGI:97168	1st branchial arch	strong	TS15	9.5 dpc	EMAGE:3839
MSX1	4	*Msx1*	MGI:97168	1st branchial arch maxillary component	moderate	TS15	9.5 dpc	EMAGE:5411
MSX1	4	*Msx1*	MGI:97168	1st branchial arch mandibular component	moderate	TS15	9.5 dpc	EMAGE:5411
MSX1	4	*Msx1*	MGI:97168	1st branchial arch mandibular component	weak	TS15	9.5 dpc	EMAGE:24726
MSX1	4	*Msx1*	MGI:97168	2nd branchial arch	weak	TS15	9.5 dpc	EMAGE:3839
MSX1	4	*Msx1*	MGI:97168	2nd branchial arch mesenchyme	moderate	TS15	9.5 dpc	EMAGE:5411
MSX1	4	*Msx1*	MGI:97168	3rd branchial arch	weak	TS15	9.5 dpc	EMAGE:3839
MSX1	4	*Msx1*	MGI:97168	1st branchial arch maxillary component	moderate	TS17	10.5dpc	EMAGE:5290
MSX1	4	*Msx1*	MGI:97168	1st branchial arch maxillary component	weak	TS17	10.5 dpc	EMAGE:26194
MSX1	4	*Msx1*	MGI:97168	1st branchial arch mandibular component	weak	TS17	10.5 dpc	EMAGE:26194
MSX1	4	*Msx1*	MGI:97168	1st branchial arch mandibular component mesenchyme	strong	TS18	10.5 dpc	EMAGE:653
MSX1	4	*Msx1*	MGI:97168	1st branchial arch maxillary component mesenchyme	strong	TS18	10.5 dpc	EMAGE:653
MSX1	4	*Msx1*	MGI:97168	1st branchial arch maxillary component mesenchyme	strong	TS18	10.5 dpc	EMAGE:3277
MSX1	4	*Msx1*	MGI:97168	1st branchial arch mandibular component ectoderm	strong	TS17	10.5dpc	EMAGE:5290

MSX1	4	*Msx1*	MGI:97168	2nd branchial arch	weak	TS17	10.5 dpc	EMAGE:26194
MSX1	4	*Msx1*	MGI:97168	branchial arch	strong	TS18	10.5 dpc	EMAGE:3518
MSX1	4	*Msx1*	MGI:97168	1st branchial arch mandibular component mesenchyme	moderate	TS18	11 dpc	EMAGE:493
MSX1	4	*Msx1*	MGI:97168	1st branchial arch maxillary component mesenchyme	moderate	TS18	11 dpc	EMAGE:493
MSX1	4	*Msx1*	MGI:97168	1st branchial arch mandibular component mesenchyme	-	TS18	11.5 dpc	EMAGE:158
MSX1	4	*Msx1*	MGI:97168	1st branchial arch maxillary component mesenchyme	-	TS18	11.5 dpc	EMAGE:158
MSX1	4	*Msx1*	MGI:97168	1st branchial arch mandibular component mesenchyme derived from neural crest	-	TS18	11.5 dpc	EMAGE:158
MSX1	4	*Msx1*	MGI:97168	1st branchial arch maxillary component mesenchyme derived from neural crest	-	TS18	11.5 dpc	EMAGE:158
MSX1	4	*Msx1*	MGI:97168	1st branchial groove	-	TS18	11.5 dpc	EMAGE:158
MSX1	4	*Msx1*	MGI:97168	2nd branchial arch mesenchyme derived from neural crest	-	TS18	11.5 dpc	EMAGE:158
MSX1	4	*Msx1*	MGI:97168	2nd branchial arch	strong	TS19	11.5 dpc	EMAGE:27695
MSX1	4	*Msx1*	MGI:97168	2nd branchial arch mesenchyme	detected	TS19	11.5 dpc	EMAGE:3278
MSX1	4	*Msx1*	MGI:97168	3rd branchial arch mesenchyme	-	TS18	11.5 dpc	EMAGE:158
MSX1	4	*Msx1*	MGI:97168	3rd branchial arch mesenchyme derived from neural crest	-	TS18	11.5 dpc	EMAGE:158
MSX1	4	*Msx1*	MGI:97168	4th branchial arch mesenchyme derived from neural crest	-	TS18	11.5 dpc	EMAGE:158
MSX1	4	*Msx1*	MGI:97168	1st branchial arch mesenchyme derived from neural crest	strong	TS14	17 Somite no.	EMAGE:628
MSX1	4	*Msx1*	MGI:97168	2nd branchial arch mesenchyme derived from neural crest	strong	TS14	17 Somite no.	EMAGE:628
SPRY1	4	*Spry1*	MGI:1345139	1st branchial arch mandibular component mesenchyme	-	TS17	10.5 dpc	EMAGE:3624
SPRY1	4	*Spry1*	MGI:1345139	1st branchial arch maxillary component mesenchyme	-	TS17	10.5 dpc	EMAGE:3624
CDK2	12	*Cdk2*	MGI:104772	1st branchial arch maxillary component	strong	TS15	9.5 dpc	EMAGE:25561
CDK2	12	*Cdk2*	MGI:104772	1st branchial arch mandibular component	strong	TS15	9.5 dpc	EMAGE:25561
CDK2	12	*Cdk2*	MGI:104772	2nd branchial arch	strong	TS15	9.5 dpc	EMAGE:25561
CDK2	12	*Cdk2*	MGI:104772	1st branchial arch mandibular component	strong	TS17	10.5 dpc	EMAGE:27046
CDK2	12	*Cdk2*	MGI:104772	1st branchial arch maxillary component	strong	TS17	10.5 dpc	EMAGE:27046
ERBB3	12	*Erbb3*	MGI:95411	branchial arch	detected	TS16	10.5 dpc	EMAGE:5299

ERBB3	12	*Erbb3*	MGI:95411	3rd branchial arch mesenchyme derived from neural crest	detected	TS16	10.5 dpc	EMAGE:5299
ERBB3	12	*Erbb3*	MGI:95411	4th branchial arch mesenchyme derived from neural crest	detected	TS16	10.5 dpc	EMAGE:5299
SPRY2	13	*Spry2*	MGI:1345138	1st branchial arch mandibular component mesenchyme	detected	TS15	9.75 dpc	EMAGE:4618
SPRY2	13	*Spry2*	MGI:1345138	1st branchial arch maxillary component mesenchyme	detected	TS15	9.75 dpc	EMAGE:4618
SPRY2	13	*Spry2*	MGI:1345138	1st branchial groove ectoderm	detected	TS15	9.75 dpc	EMAGE:4618
SPRY2	13	*Spry2*	MGI:1345138	1st branchial pouch	detected	TS15	9.75 dpc	EMAGE:4618
SPRY2	13	*Spry2*	MGI:1345138	2nd branchial arch	detected	TS15	9.75 dpc	EMAGE:4618
SPRY2	13	*Spry2*	MGI:1345138	2nd branchial groove ectoderm	detected	TS15	9.75 dpc	EMAGE:4618
SPRY2	13	*Spry2*	MGI:1345138	2nd branchial pouch	detected	TS15	9.75 dpc	EMAGE:4618
SPRY2	13	*Spry2*	MGI:1345138	3rd branchial arch	detected	TS15	9.75 dpc	EMAGE:4618
SPRY2	13	*Spry2*	MGI:1345138	3rd branchial groove ectoderm	detected	TS15	9.75 dpc	EMAGE:4618
SPRY2	13	*Spry2*	MGI:1345138	3rd branchial pouch	detected	TS15	9.75 dpc	EMAGE:4618
GSC	14	*Gsc*	MGI:95841	1st branchial arch	detected	TS17	10.5 dpc	EMAGE:732
GSC	14	*Gsc*	MGI:95841	1st branchial arch	detected	TS17	10.5 dpc	EMAGE:3973
GSC	14	*Gsc*	MGI:95841	1st branchial arch mandibular component	strong	TS17	10.5 dpc	EMAGE:26593
GSC	14	*Gsc*	MGI:95841	1st branchial arch maxillary component	strong	TS17	10.5 dpc	EMAGE:26593
GSC	14	*Gsc*	MGI:95841	2nd branchial arch	detected	TS17	10.5 dpc	EMAGE:732
GSC	14	*Gsc*	MGI:95841	2nd branchial arch	detected	TS17	10.5 dpc	EMAGE:3973
GSC	14	*Gsc*	MGI:95841	2nd branchial arch	strong	TS17	10.5 dpc	EMAGE:26593
GSC	14	*Gsc*	MGI:95841	branchial arch	strong	TS17	10.5 dpc	EMAGE:732
GSC	14	*Gsc*	MGI:95841	1st branchial groove	strong	TS19	11.5 dpc	EMAGE:5405
GSC	14	*Gsc*	MGI:95841	2nd branchial arch	strong	TS19	11.5 dpc	EMAGE:28094
WNT9B	17	*Wnt9b*	MGI:1197020	1st branchial arch maxillary component	strong	TS17	10.5 dpc	EMAGE:4987
WNT9B	17	*Wnt9b*	MGI:1197020	1st branchial arch maxillary component	strong	TS18	10.5 dpc	EMAGE:5279
WNT9B	17	*Wnt9b*	MGI:1197020	1st branchial arch mandibular component	strong	TS17	10.5 dpc	EMAGE:4987
WNT9B	17	*Wnt9b*	MGI:1197020	1st branchial arch mandibular component ectoderm	strong	TS18	10.5 dpc	EMAGE:5279

WNT9B	17	*Wnt9b*	MGI:1197020	2nd branchial arch	strong	TS17	10.5 dpc	EMAGE:4987
WNT9B	17	*Wnt9b*	MGI:1197020	3rd branchial arch	strong	TS17	10.5 dpc	EMAGE:4987
MAFB	20	*Mafb*	MGI:1914981	1st branchial arch maxillary component	strong	TS17	10.5 dpc	EMAGE:26188
**maxilla**								
IRF6	1	*Irf6*	MGI:1859211	maxillary process	strong	TS19	11.5 dpc	EMAGE:27747
MSX1	4	*Msx1*	MGI:97168	maxillary process	strong	TS19	11.5 dpc	EMAGE:27695
MSX1	4	*Msx1*	MGI:97168	maxillary process	detected	TS19	11.5 dpc	EMAGE:3278
GSC	14	*Gsc*	MGI:95841	maxillary process	strong	TS19	11.5 dpc	EMAGE:5405
GSC	14	*Gsc*	MGI:95841	maxillary process	strong	TS19	11.5 dpc	EMAGE:28094
GSC	14	*Gsc*	MGI:95841	maxillary process epithelium	strong	TS20	12.5 dpc	EMAGE:5390
WNT9B	17	*Wnt9b*	MGI:1197020	maxillary process	strong	TS19	11.5 dpc	EMAGE:4989
WNT9B	17	*Wnt9b*	MGI:1197020	maxillary process	moderate	TS20	12.5 dpc	EMAGE:4984
WNT9B	17	*Wnt9b*	MGI:1197020	maxillary process epithelium	moderate	TS20	12.5 dpc	EMAGE:4984
MAFB	20	*Mafb*	MGI:1914981	maxillary process	strong	TS19	11.5 dpc	EMAGE:27689
**mandible**								
IRF6	1	*Irf6*	MGI:1859211	lower jaw molar	strong	TS23	14.5 dpc	EMAGE:6816
MSX1	4	*Msx1*	MGI:97168	mandibular process	strong	TS19	11.5 dpc	EMAGE:27695
MSX1	4	*Msx1*	MGI:97168	mandibular process	moderate	TS19	11.5 dpc	EMAGE:5291
MSX1	4	*Msx1*	MGI:97168	mandibular process mesenchyme	detected	TS19	11.5 dpc	EMAGE:3278
KRT18	12	*Krt18*	MGI:96692	lower jaw incisor	moderate	TS23	14.5 dpc	EMAGE:11923
KRT18	12	*Krt18*	MGI:96692	lower jaw molar	moderate	TS23	14.5 dpc	EMAGE:11923
GSC	14	*Gsc*	MGI:95841	mandibular process	strong	TS19	11.5 dpc	EMAGE:5405
GSC	14	*Gsc*	MGI:95841	mandibular process	strong	TS19	11.5 dpc	EMAGE:28094
WNT9B	17	*Wnt9b*	MGI:1197020	mandibular process	strong	TS19	11.5 dpc	EMAGE:4989

**lip**								
MSX1	4	*Msx1*	MGI:97168	rest of upper lip	strong	TS21	13.5 dpc	EMAGE:5413
MSX1	4	*Msx1*	MGI:97168	rest of upper lip	strong	TS22	-	EMAGE:5414
WNT9B	17	*Wnt9b*	MGI:1197020	upper lip	moderate	TS21	13.5 dpc	EMAGE:4985
WNT9B	17	*Wnt9b*	MGI:1197020	upper lip	strong	TS22	14.5 dpc	EMAGE:4986
MAFB	20	*Mafb*	MGI:1914981	lower lip	moderate	TS23	14.5 dpc	EMAGE:19184
**palate**								
KRT18	12	*Krt18*	MGI:96692	palatal shelf epithelium	moderate	TS23	14.5 dpc	EMAGE:11923
GSC	14	*Gsc*	MGI:95841	palatal shelf	strong	TS20	12.5 dpc	EMAGE:5390
GSC	14	*Gsc*	MGI:95841	palatal shelf	weak	TS21	13.5 dpc	EMAGE:5391
WNT9B	17	*Wnt9b*	MGI:1197020	primary palate epithelium	weak	TS20	12.5 dpc	EMAGE:4984
WNT9B	17	*Wnt9b*	MGI:1197020	palatal shelf epithelium	weak	TS21	13.5 dpc	EMAGE:4985
**oral**								
IRF6	1	*Irf6*	MGI:1859211	oral epithelium	strong	TS23	14.5 dpc	EMAGE:6816
VAX1	10	*Vax1*	MGI:1277163	oral region epithelium	detected	TS17	12.5 dpc	EMAGE:542
KRT18	12	*Krt18*	MGI:96692	oral epithelium	moderate	TS23	14.5 dpc	EMAGE:11923
KRT8	12	*Krt8*	MGI:1921377	oral epithelium	moderate	TS23	14.5 dpc	EMAGE:10450
**frontal process**								
MSX1	4	*Msx1*	MGI:97168	frontal process mesenchyme	-	TS18	11.5 dpc	EMAGE:158
**philtrum**								
MSX1	4	*Msx1*	MGI:97168	philtrum	weak	TS21	13.5 dpc	EMAGE:5413
**<u>others</u>**								

**nose**								
IRF6	1	*Irf6*	MGI:1859211	naris	strong	TS23	14.5 dpc	EMAGE:6816
MSX1	4	*Msx1*	MGI:97168	nose	strong	TS20	12.5 dpc	EMAGE:5412
MSX1	4	*Msx1*	MGI:97168	nose	-	TS18	11.5 dpc	EMAGE:158
BAG4	8	*Bag4*	MGI:1914634	naris	strong	TS23	14.5 dpc	EMAGE:10095
VAX1	10	*Vax1*	MGI:1277163	nose	detected	TS17	12.5 dpc	EMAGE:542
KRT18	12	*Krt18*	MGI:96692	naris	strong	TS23	14.5 dpc	EMAGE:11923
ERBB3	12	*Erbb3*	MGI:95411	anterior naris epithelium	moderate	TS23	14.5 dpc	EMAGE:16072
ERBB3	12	*Erbb3*	MGI:95411	external naris epithelium	moderate	TS23	14.5 dpc	EMAGE:16072
WNT9B	17	*Wnt9b*	MGI:1197020	nose	strong	TS22	14.5 dpc	EMAGE:4986
**nasal**								
MSX1	4	*Msx1*	MGI:97168	fronto-nasal process	weak	TS15	9.5 dpc	EMAGE:3839
MSX1	4	*Msx1*	MGI:97168	medial-nasal process	strong	TS18	10.5 dpc	EMAGE:653
MSX1	4	*Msx1*	MGI:97168	medial-nasal process	strong	TS18	10.5 dpc	EMAGE:3277
MSX1	4	*Msx1*	MGI:97168	medial-nasal process	strong	TS17	10.5dpc	EMAGE:5290
MSX1	4	*Msx1*	MGI:97168	latero-nasal process	strong	TS18	10.5 dpc	EMAGE:653
MSX1	4	*Msx1*	MGI:97168	latero-nasal process	strong	TS18	10.5 dpc	EMAGE:3277
MSX1	4	*Msx1*	MGI:97168	latero-nasal process	strong	TS17	10.5dpc	EMAGE:5290
MSX1	4	*Msx1*	MGI:97168	medial-nasal process mesenchyme	moderate	TS18	11 dpc	EMAGE:493
MSX1	4	*Msx1*	MGI:97168	latero-nasal process mesenchyme	moderate	TS18	11 dpc	EMAGE:493
MSX1	4	*Msx1*	MGI:97168	nasal epithelium	-	TS18	11.5 dpc	EMAGE:158
MSX1	4	*Msx1*	MGI:97168	medial-nasal process	detected	TS19	11.5 dpc	EMAGE:3278
MSX1	4	*Msx1*	MGI:97168	medial-nasal process	strong	TS19	11.5 dpc	EMAGE:5291
MSX1	4	*Msx1*	MGI:97168	medial-nasal process mesenchyme	-	TS18	11.5 dpc	EMAGE:158
MSX1	4	*Msx1*	MGI:97168	latero-nasal process	detected	TS19	11.5 dpc	EMAGE:3278

MSX1	4	*Msx1*	MGI:97168	latero-nasal process	strong	TS19	11.5 dpc	EMAGE:5291
MSX1	4	*Msx1*	MGI:97168	latero-nasal process mesenchyme	-	TS18	11.5 dpc	EMAGE:158
MSX1	4	*Msx1*	MGI:97168	nasal cavity epitheliumnasal cavity epithelium	detected	TS20	12.5 dpc	EMAGE:646
SPRY1	4	*Spry1*	MGI:1345139	fronto-nasal process	-	TS19	10.5 dpc	EMAGE:1252
FGF10	5	*Fgf10*	MGI:1099809	nasal epithelium	detected	TS17	10.5 dpc	EMAGE:3769
VAX1	10	*Vax1*	MGI:1277163	fronto-nasal process	detected	TS15	9.5 dpc	EMAGE:253
VAX1	10	*Vax1*	MGI:1277163	fronto-nasal process ectoderm	detected	TS15	9.5 dpc	EMAGE:253
SPRY2	13	*Spry2*	MGI:1345138	fronto-nasal process	detected	TS18	10.5 dpc	EMAGE:4619
GSC	14	*Gsc*	MGI:95841	fronto-nasal process	detected	TS17	10.5 dpc	EMAGE:3973
GSC	14	*Gsc*	MGI:95841	medial-nasal process	strong	TS19	11.5 dpc	EMAGE:5405
GSC	14	*Gsc*	MGI:95841	latero-nasal process	strong	TS19	11.5 dpc	EMAGE:5405
WNT9B	17	*Wnt9b*	MGI:1197020	medial-nasal process	strong	TS17	10.5 dpc	EMAGE:4987
WNT9B	17	*Wnt9b*	MGI:1197020	medial-nasal process	strong	TS18	10.5 dpc	EMAGE:5279
WNT9B	17	*Wnt9b*	MGI:1197020	latero-nasal process	strong	TS17	10.5 dpc	EMAGE:4987
WNT9B	17	*Wnt9b*	MGI:1197020	latero-nasal process	strong	TS18	10.5 dpc	EMAGE:5279
WNT9B	17	*Wnt9b*	MGI:1197020	medial-nasal process	weak	TS19	11.5 dpc	EMAGE:4989
WNT9B	17	*Wnt9b*	MGI:1197020	latero-nasal process	moderate	TS19	11.5 dpc	EMAGE:4989
NOG	17	*Nog*	MGI:104327	nasal septum	weak	TS23	14.5 dpc	EMAGE:8431
**maxilla**								
PTCH1	9	*Ptch1*	MGI:105373	maxilla	moderate	TS23	14.5 dpc	EMAGE:16248
PTCH1	9	*Ptch1*	MGI:105373	maxilla	-	TS23	14.5 dpc	EMAGE:21444
PTCH1	9	*Ptch1*	MGI:105373	upper jaw	-	TS23	14.5 dpc	EMAGE:21444
**mandible**								
IRF6	1	*Irf6*	MGI:1859211	lower jaw molar	strong	TS23	14.5 dpc	EMAGE:6816
MMP16	8	*Mmp16*	MGI:1276107	mandible	moderate	TS23	14.5 dpc	EMAGE:7073

PTCH1	9	*Ptch1*	MGI:105373	mandible	moderate	TS23	14.5 dpc	EMAGE:16248
PTCH1	9	*Ptch1*	MGI:105373	mandible	-	TS23	14.5 dpc	EMAGE:21444
PTCH1	9	*Ptch1*	MGI:105373	lower jaw	-	TS23	14.5 dpc	EMAGE:21444
KRT18	12	*Krt18*	MGI:96692	lower jaw incisor	moderate	TS23	14.5 dpc	EMAGE:11923
KRT18	12	*Krt18*	MGI:96692	lower jaw molar	moderate	TS23	14.5 dpc	EMAGE:11923
NOG	17	*Nog*	MGI:104327	mandible	weak	TS23	14.5 dpc	EMAGE:8431
**tooth**								
IRF6	1	*Irf6*	MGI:1859211	upper jaw incisor	strong	TS23	14.5 dpc	EMAGE:6816
IRF6	1	*Irf6*	MGI:1859211	upper jaw molar	strong	TS23	14.5 dpc	EMAGE:6816
PTCH1	9	*Ptch1*	MGI:105373	upper jaw incisor	weak	TS23	14.5 dpc	EMAGE:16248
PTCH1	9	*Ptch1*	MGI:105373	lower jaw incisor	weak	TS23	14.5 dpc	EMAGE:16248
KRT18	12	*Krt18*	MGI:96692	upper jaw incisor	moderate	TS23	14.5 dpc	EMAGE:11923
KRT18	12	*Krt18*	MGI:96692	upper jaw molar	moderate	TS23	14.5 dpc	EMAGE:11923
**embryo**								
MSX1	4	*Msx1*	MGI:97168	surface ectoderm	strong	TS15	9.5 dpc	EMAGE:548
MSX1	4	*Msx1*	MGI:97168	surface ectoderm	detected	TS15	9.5 dpc	EMAGE:647
MSX1	4	*Msx1*	MGI:97168	embryo	detected	TS21	12.5 dpc	EMAGE:3844
MSX1	4	*Msx1*	MGI:97168	extraembryonic component	strong	TS14	17 Somite no.	EMAGE:628
SPRY1	4	*Spry1*	MGI:1345139	embryo	-	TS15	9.5 dpc	EMAGE:4873
SPRY1	4	*Spry1*	MGI:1345139	embryo	-	TS17	10.5 dpc	EMAGE:4874
SPRY1	4	*Spry1*	MGI:1345139	embryo	-	TS19	11.5 dpc	EMAGE:4875
SPRY1	4	*Spry1*	MGI:1345139	embryo	-	TS20	12.5 dpc	EMAGE:4876
FGF10	5	*Fgf10*	MGI:1099809	embryo	detected	TS17	10.5 dpc	EMAGE:6108
FGF10	5	*Fgf10*	MGI:1099809	surface ectoderm	detected	TS17	10.5 dpc	EMAGE:3769
FGF10	5	*Fgf10*	MGI:1099809	embryo		TS23	14.5 dpc	EMAGE:9080

PTCH1	9	*Ptch1*	MGI:105373	embryo	detected	TS17	9.0 dpc	EMAGE:813
PTCH1	9	*Ptch1*	MGI:105373	embryo	detected	TS17	9.0 dpc	EMAGE:3423
PTCH1	9	*Ptch1*	MGI:105373	embryo	strong	TS18	10.5 dpc	EMAGE:3696
PTCH1	9	*Ptch1*	MGI:105373	embryo	-	TS23	14.5 dpc	EMAGE:21444
VAX1	10	*Vax1*	MGI:1277163	embryo	detected	TS12	8.0 dpc	EMAGE:252
VAX1	10	*Vax1*	MGI:1277163	surface ectoderm	detected	TS12	8.0 dpc	EMAGE:252
KRT8	12	*Krt8*	MGI:1921377	embryo	weak	TS23	14.5 dpc	EMAGE:10450
RPS26	12	*Rps26*	MGI:1351628	embryo	strong	TS23	14.5 dpc	EMAGE:11827
ERBB3	12	*Erbb3*	MGI:95411	embryo	detected	TS17	10.5 dpc	EMAGE:6113
ERBB3	12	*Erbb3*	MGI:95411	extraembryonic component	moderate	TS23	14.5 dpc	EMAGE:16072
SPRY2	13	*Spry2*	MGI:1345138	embryo	detected	TS15	9.5 dpc	EMAGE:4868
SPRY2	13	*Spry2*	MGI:1345138	embryo	detected	TS17	10.5 dpc	EMAGE:4869
SPRY2	13	*Spry2*	MGI:1345138	embryo	detected	TS19	10.5 dpc	EMAGE:4870
SPRY2	13	*Spry2*	MGI:1345138	embryo	detected	TS20	12.5 dpc	EMAGE:4872
DICER1	14	*Dicer1*	MGI:2177178	embryo	detected	TS16	10.5 dpc	EMAGE:4991
GSC	14	*Gsc*	MGI:95841	primitive endoderm	detected	TS09	6.5 dpc	EMAGE:89
GSC	14	*Gsc*	MGI:95841	embryo	detected	TS11	HF Downs & Davies	EMAGE:3510
GSC	14	*Gsc*	MGI:95841	embryo endoderm	detected	TS11	HF Downs & Davies	EMAGE:3510
GSC	14	*Gsc*	MGI:95841	embryo mesoderm	detected	TS11	HF Downs & Davies	EMAGE:3510
CREBBP	16	*Cerbbp*	MGI:1098280	embryo	detected	TS15	9.5 dpc	EMAGE:4846
WNT9B	17	*Wnt9b*	MGI:1197020	embryo	detected	TS15	9.5 dpc	EMAGE:6158
WNT9B	17	*Wnt9b*	MGI:1197020	embryo	detected	TS17	10.5 dpc	EMAGE:6157
WNT9B	17	*Wnt9b*	MGI:1197020	embryo	detected	TS19	11.5 dpc	EMAGE:3963

WNT9B	17	*Wnt9b*	MGI:1197020	embryo	-	TS23	14.5 dpc	EMAGE:9281
NOG	17	*Nog*	MGI:104327	embryo mesoderm	detected	TS11	7.75 dpc	EMAGE:747
**cranium**								
MMP16	8	*Mmp16*	MGI:1276107	cranium	strong	TS23	14.5 dpc	EMAGE:7073
NOG	17	*Nog*	MGI:104327	viscerocranium	weak	TS23	14.5 dpc	EMAGE:8431

Data was derived from EMAGE database (http://www.emouseatlas.org/emage/home.php).

Supplementary Table 12. Mutant mouse models with OFC malformations from MGI database.

Gene	Chr.	Allelic Composition (Genetic Background)	Annotated Term	Reference	Cleft rate (%)	PMID
**Cleft palate:**						
IRF6	1	$Irf6^{clft1}/Irf6^{clft1}$	cleft secondary palate	J:160190	-	20196077
IRF6	1	$Irf6^{Gt(OST398253)Lex}/Irf6^{Gt(OST398253)Lex}$	failure of palatal shelf elevation	J:160190	-	20196077
IRF6	1	$Irf6^{tm1Mjd}/Irf6^{tm1Mjd}$	failure of palatal shelf elevation	J:160190	-	20196077
IRF6	1	Irf6tm1Mjd/Irf6+<br>SfnEr/Sfn+<br>(involves: 129S1/Sv * 129X1/SvJ * C57BL/6)	cleft palate	J:116077	-	17041603
IRF6	1	$Irf6^{tm1Mjd}/Irf6^{tm1Mjd}$	cleft secondary palate	J:116077	-	17041603
IRF6	1	$Irf6^{Gt(OST398253)Lex}/Irf6^{Gt(OST398253)Lex}$<br>(involves: 129S5/SvEvBrd * C57BL/6)	cleft secondary palate	J:115343	-	17041601
MSX1	4	$Msx1^{tm1Rilm}/Msx1^{tm1Rilm}$	abnormal palatal shelf fusion at midline	J:17489	100	7914451
MSX1	4	(either: (involves: 129S4/SvJae-$Msx1^{tm1Rilm}$) or (involves: 129S4/SvJae * BALB/c) or (involves: 129X1/SvJae * C57BL/6J))	cleft secondary palate	J:17489	100	7914451
MSX1	4	$Dlx5^{tm1Levi}/Dlx5^{tm1Levi}$	cleft secondary palate	J:104286	-	16330189

		$Msx1^{tm1Bero}$/$Msx1^{tm1Bero}$				
MSX1	4	$Msx1^{tm1Bero}$/$Msx1^{tm1Bero}$	cleft secondary palate	J:42035	100	9256350
				J:104286	-	16330189
		(B6.129P2-$Msx1^{tm1Bero}$)	palatal shelf hypoplasia	J:104286	-	16330189
MSX1	4	$Msx1^{tm1Bero}$/$Msx1^{tm1Bero}$	cleft palate	J:136241	-	18442049
		$Msx2^{tm1Bero}$/$Msx2^{tm1Yvla}$				
		(involves: 129/Sv * BALB/c * C57BL/6 * SJL)				
MSX1	4	$Msx1^{tm1Rem}$/$Msx1^{tm1Rem}$	cleft palate	J:128624	-	17654563
		Tg(Msx2-cre)5Rem/0				
		(involves: 129S6/SvEvTac * C57BL/6 * SJL)				
MSX1	4	$Msx1^{tm2.1(cre/ERT2)Bero}$/$Msx1^{tm2.1(cre/ERT2)Bero}$	cleft secondary palate	J:194129	-	23090744
		$Msx2^{tm1Bero}$/$Msx2^{tm1Bero}$				
		(involves: 129/Sv * 129P2/OlaHsd * 129S2/SvPas * C57BL/6)				
FGF10	5	$Fgf10^{tm1Wss}$/$Fgf10^{tm1Wss}$	cleft secondary palate	J:90909	100	15199404
FGFR1	8	$Fgfr1^{tm1Jpa}$/$Fgfr1^{tm1.1Jpa}$	cleft palate	J:81179	80	12514106
		Tg(Wnt1-cre)11Rth/0				
		(involves: 129S1/Sv * 129X1/SvJ * C57BL/6 * CBA * ICR)				
FGFR1	8	$Fgfr1^{tm1Jpa}$/$Fgfr1^{tm1Jpa}$	cleft palate	J:81179	80	12514106
		Tg(Wnt1-cre)11Rth/0				
		(involves: 129S1/Sv * 129X1/SvJ * C57BL/6 * CBA * ICR)				
FGFR1	8	$Fgfr1^{tm2Jrt}$/$Fgfr1^{tm2Jrt}$	cleft palate	J:81179	80	12514106
FGFR1	8	Pdgfratm9(Pdgfra/Fgfr1)Sor/Pdgfra+	cleft palate	J:83560	100	12748302
		(either: (involves: 129S4/SvJaeSor) or (involves: 129S4/SvJaeSor * C57BL/6))				
MMP16	8	$Mmp16^{tm1Khol}$/$Mmp16^{tm1Khol}$	palatal shelf hypoplasia	J:130212	80	18022611
NOG	17	Osr2tm2(cre)Jian/Osr2+	cleft palate	J:149232	-	19341725

		Tg(CAG-Nog)1Ych/0<br>(involves: 129S1/Sv * 129S7/SvEvBrd * C57BL/6)				
PTCH1	9	Ptch1^{dl}/Ptch1^{dl}	absent palatal shelf	J:204468	10	23897749
PTCH1	9	Ptch1^{dl}/Ptch1^{tm1Mps}<br>(involves: 129S1/SvImJ * 129X1/SvJ * C57BL/6J)	palatal shelf hypoplasia	J:204468	10	23897749
VAX1	10	Vax1^{tm1Grl}/Vax1^{tm1Grl}<br>(involves: 129S1/Sv * C57BL/6)	cleft palate	J:58996	100	10601035
VAX1	10	Vax1^{tm1Grl}/Vax1^{tm1Grl}<br>Vax2^{tm1Grl}/Vax2^{tm1Grl}<br>(involves: 129S1/Sv)	cleft palate	J:98488	-	15905411
VAX1	10	Vax1^{tm1Pgr}/Vax1^{tm1Pgr}<br>(either: (involves: 129S1/Sv * 129X1/SvJ * C57BL/6) or (involves: 129S1/Sv * 129X1/SvJ * NMRI))	cleft palate	J:58997	100	10601036
SPRY2	13	Ednrbs-36Pub/Ednrb+<br>Spry2tm1.1Mrt/Spry2+<br>(involves: 101/Rl * 129P2/OlaHsd * C3H/Rl * C57BL/6J)	cleft secondary palate	J:125229	-	17693063
SPRY2	13	Ednrb$^{s-36Pub}$/Ednrb$^{s-36Pub}$<br>Tg(Spry2)2Tpo/0<br>(B6.Cg-Ednrb$^{s-36Pub}$ Tg(Spry2)2Tpo)	cleft secondary palate	J:125229	-	17693063
SPRY2	13	Ednrb$^{s-36Pub}$/Ednrb$^{s-36Pub}$<br>Tg(Spry2)69Tpo/0<br>(B6.Cg-Ednrb$^{s-36Pub}$ Tg(Spry2)69Tpo)	cleft secondary palate	J:125229	-	17693063
GSC;<br>*MSX1*	14; 4	Gsc^{tm1Pgr}/Gsc^{tm1Pgr}<br>Msx1^{tm1Rilm}/Msx1^{tm1Rilm}<br>(involves: 129S1/Sv * 129S4/SvJae * 129X1/SvJ)	cleft palate	J:59750	-	10512191
CREBBP	16	Crebbp^{tm2Pkb}/Crebbp^{tm2Pkb}<br>(involves: 129P2/OlaHsd * C57BL/6)	cleft secondary palate	J:103607	50	16237459

WNT9B	17	Pbx1tm1Mlc/Pbx1+<br>Wnt9b^{tm1Amc}/Wnt9b^{tm1Amc}<br>Foxg1tm1(cre)Skm/Foxg1+<br>(involves: 129P2/OlaHsd * 129S6/SvEvTac * 129X1/SvJ)	cleft palate	J:178316	100	21982646
WNT9B	17	Wnt9b^{tm1Amc}/Wnt9b^{tm1Amc}<br>Foxg1tm1(cre)Skm/Foxg1+<br>(involves: 129P2/OlaHsd * 129X1/SvJ)	cleft palate	J:178316	100	21982646
WNT9B	17	Wnt9b$^{tm1.1Amc}$/Wnt9b$^{tm1.1Amc}$<br>(involves: 129X1/SvJ * C57BL/6 * CBA)	cleft palate	J:100575	-	16054034
**Cleft lip:**						
WNT9B	17	Pbx1tm1Mlc/Pbx1+<br>Wnt9b^{tm1Amc}/Wnt9b^{tm1Amc}<br>Foxg1tm1(cre)Skm/Foxg1+<br>(involves: 129P2/OlaHsd * 129S6/SvEvTac * 129X1/SvJ)	cleft upper lip	J:178316	100	21982646
WNT9B	17	Wnt9b^{tm1Amc}/Wnt9b^{tm1Amc}<br>Foxg1tm1(cre)Skm/Foxg1+<br>(involves: 129P2/OlaHsd * 129X1/SvJ)	cleft upper lip	J:178316	-	21982646
WNT9B	17	Wnt9b^{clf1}/Wnt9b^{clf1}<br>(AEJ.A/Jur)	cleft upper lip	J:22596<br>J:92341<br>J:69826	-<br>24<br>24	3491125<br>15329828<br>11353389
WNT9B	17	Wnt9b^{clf1}/Wnt9b^{clf1}<br>clf2/clf2<br>(AEJ.A/Jur)	cleft upper lip	J:25484<br>J:92341<br>J:69826	20<br>24<br>24	7601909<br>15329828<br>11353389
WNT9B	17	Wnt9b^{clf1}/Wnt9b^{clf1}	cleft upper lip	J:92341	24	15329828

		clf2/clf2+(AEJ.A/Jur)		J:69826	24	11353389
WNT9B	17	$Wnt9b^{tm1.1Amc}/Wnt9b^{tm1.1Amc}$ (involves: 129X1/SvJ * C57BL/6 * CBA)	cleft upper lip	J:100575	-	16054034

Data was derived from the database of MGI (http://www.informatics.jax.org/).

Supplementary Table 13. Syndromic OFC from OMIM database.

Gene	Chr.	CL/P Syndrome	Phenotype OMIM number	Main phenotypes	PMID	Cleft type
IRF6	1	Van der Woude syndrome	119300	CL/P, lip pit	12219090	CL/P
		Popliteal pterygium syndrome	119500	popliteal/intercruralpterygium, syndactyly fingers and toes, congenital ankyloblepharonfiliforme, cryptorchidism, bifid scrotum, hypoplastic vagina/uterus, lip cyst, CL/P, lip pit	12219090	CL/P
MSX1	4	Tooth agenesis, selective, 1, with or without orofacial cleft	106600	tooth agenesis, CL/P	10742093	CL/P
TFAP2A	6	Branchio-oculo-facial syndrome	113620	branchial cleft sinus defects, ocular anomalies, CL/P	24783654	CL/P
FGFR1	8	Hartsfield syndrome	615465	holoprosencephaly, ectrodactyly, mental retardation, CL/P	19504604	CL/P
		Kallmann syndrome	147950	idiopathic hypogonadotropichypogonadism, anosmia, sensorineural hearing loss, CL/P	12627230<br>15001591	CL/P<br>CPO
PTCH1	9	Gorlin syndrome	109400	basal cell carcinomas, jaw cysts, ovarian calcification or fibroma, ophthalmic abnormalities, CL/P	8681379	CL/P
VAX1	10	Microphthalmia, syndromic 11	614402	microphthalmia, small optic nerve, CLP, agenesis of corpus callosum, height in third centile, weight in third centile, head circumference in third centile	22095910	CLP
RPS26	12	Diamond-Blackfan anemia 10	613309	macrocytic anemia, increased fetal hemoglobin, increased erythrocyte adenosine deaminase activity, reticulocytopenia, bone marrow shows decreased erythroid progenitors, short stature, CPO	24942156	CPO

Data was derived from Online Mendelian Inheritance In Man database (OMIM, http://omim.org/).

Supplementary Table 14. Biological categories by literature review.

Category	Chr.	Description	PMID
<u>**morphogenesis and development**</u>			
**bone morphogenesis**			
IRF6	1	mutations associated with impaired morphogenesis of the lip and palate; mutations may lead to abolish the DNA binding activity in the developing craniofacial region.	12964020; 12219090
MSX1	4	encodes a transcription factor that is highly expressed during embryogenesis and postnatal development in bone.	24929242
MSX1	4	Mutations of the MSX1 gene in humans are associated with cleft palate and (or) tooth agenesis.	24929242; 7914451
MMP16	8	Combined loss of MT1-MMP and MT3-MMP leads to severe craniofacial developmental defects.	18022611
VAX1	10	associated with non-syndromicorofacial clefts.	25171615; 25081408; 22095910
SUOX	12	The findings suggest an important role for sulfite oxidase (SUOX) in bone development.	20865119
DICER1	14	Loss of Dicer1 activity in Col2a1 chondrocytes causes lethal tracheomalacia.	21945074
WNT9B	17	a role for WNT genes in the pathogenesis of cleft lip/palate.	20890934; 18413325; 18181213; 16998816
WNT9B	17	regulatory link between WNT9B and FGF signaling during lip and upper jaw development; regulate midfacial development and lip fusion.	22461561; 16496313
NOG	17	too little expression of NOG, a growth antagonist, or overexpression of GDF5, a growth agonist, results in cartilage overgrowth and bony fusion.	17994231
<u>**Signaling**</u>			
**MAPK signaling**			
FGF2	4	FGF2 differentiates into tenocytes and the MAPK pathway is key in differentiation.	24008926

SPRY1	4	augmenting ERK-MAP kinase activity through inhibition of SPRY1; downstream of the GRB2-SOS complex to selectively uncouple growth factor signals from Ras activation and the MAPK pathway.	19043405; 11585837
FGF10	5	stimulatespreadipocyte proliferation through the Ras/MAPK pathway;also stimulates adipogenesis by inducing the expression of pRb through the Ras/MAPK pathway.	21696361
FGFR1	8	FGFR1-induced epithelial to mesenchymal transition through MAPK/PLCγ/COX-2-mediated mechanisms.	22701738
GADD45G	9	Gadd45 proteins are associated with signaling mediated by p38 mitogen-activated protein kinases (MAPK). Gadd45 proteins can contribute to p38 activation either by activation of upstream kinase(s) or by direct interaction.	24104470
SPRY2	13	ERK-MAPK signaling pathway was regulated by Sprouty2 ; Inactivation of Spry2 accelerates AKT-induced hepatocarcinogenesis via activation of MAPK and PKM2 pathways.	23239100; 22617155
**BMP signaling**			
MSX1	4	MSX1 expression was regulated by BMP signaling, and in particular that its expression was upregulated by SMAD8 activity.	16101586
NOG	17	noggin inhibits BMP signaling by blocking the molecular interfaces of the binding epitopes for both type I and type II receptors.	12478285
**WNT signaling**			
GSC	14	Higher gsc doses resulted in complete secondary axes and long-range signaling, correlating with repression of BMP and Wnt signals.	19369398
WNT9B	17	Wnt9b signal through the canonical Wnt signaling pathway to regulate midfacial development and lip fusion.	16496313
**epidermal growth factor receptor signaling pathway**			
IRF6	1	a transcription factor required for the differentiation of skin	22931925; 17041601
SPRY1	4	modulators and feedback inhibitors of signalling by epidermal growth factor (EGF) and fibroblast growth factor (FGF).	16954433
ERBB3	12	a member of the epidermal growth factor receptor family.	25000258
SPRY2	13	feedback regulator of EGFR trafficking and signaling; EGFR degradation through downregulation of the	23868981; 19364817

		EGFR-stabilizing protein Sprouty2 (Spry2).	
**ERK1/2 cascade**			
SPRY1	4	deficiency led to heightened EPO/EPOR activation of Erk1/2.	22508938
FGF10	5	FGF10 mediated RAS-regulated ERK1/2 signaling.	21764747
SPRY2	13	function to modulate signaling via the RAS/ERK1/2.	23150596
**fibroblast growth factor receptor signaling pathway**			
SPRY1	4	modulators and feedback inhibitors of signalling by epidermal growth factor (EGF) and fibroblast growth factor (FGF).	16954433
FGF10	5	FGF10 is an FGFR ligand; Shh is a downstream target of Fgf10/Fgfr2b signaling.	15199404; 16501574
FGFR1	8	FGF signaling mediates cilia length through an Fgf8/Fgf24-Fgfr1-intraflagellar transport pathway.	OMIM; 19242413
ERBB3	12	Erbb3 is a functional component of FGFR2-amplified cell growth.	18381441
SPRY2	13	antagonism of FGF signaling.	15809037
NOG	17	noggin expression is suppressed by FGF2 and syndromic FGFR signaling.	12687003
**Notch signaling pathway**			
CREBBP	16	Altered CREBBP structure resulted in changes in expression of basal levels of components of Notch.	20207472
**Rab protein signal transduction**			
RAB5B	12	presumably involved in vesicular trafficking at the plasma membrane.	1541686
**Ras protein signal transduction**			
FGF2	4	the FGF2-Ras-MAPK-dependent mechanism that maintains and modulates the LEC trait.	24357720
SPRY1	4	inhibited the Ras/Raf (164760)/Mapk pathway by preventing Ras activation.	11585837; 12402043
CDK2	12	ras-induced tumors are more susceptible to CDK2 ablation than myc-induced tumors; Ras, along with the activation of additional pathways, is required for the generation of G1 CDK activity.	17942901; 9163430
SPRY2	13	inhibited the Ras/Raf (164760)/Mapk pathway by preventing Ras activation.	11585837; 12402043
**<u>cell-related</u>**			
**apoptosis process**			
MSX1	4	msx1 promotes cell death.	15501222; 15705871

CCDC26	8	prominent expression in embryo; Knockdown of RAM promoted 9-cis RA-induced differentiation and TRAIL-triggered apoptosis.	16449964
GADD45G	9	encodes a ubiquitously expressed protein that is often induced by DNA damage and other stress signals associated with growth arrest and apoptosis.	24104470
PTCH1	9	Ptch1 promotes cell and tissue-specific apoptosis via its regulation of Caspase9 (Casp9) activity and mitochondrial function.	25292199
KRT18	12	Keratins 8 and 18 (K8/K18) provide anti-apoptotic functions upon liver injury.	20538000
KRT8	12	Keratin-deficient mice displayed significantly lower numbers of apoptotic hepatocytes than wt animals; Keratins 8 and 18 (K8/K18) provide anti-apoptotic functions upon liver injury.	22449798; 20538000
NTN1	17	functioned in cell survival by inhibiting the proapoptotic activity of Dcc.	18796601
**cell cycle**			
IRF6	1	promotes cell cycle arrest and is regulated by the proteasome in a cell cycle-dependent manner.	18212048
GADD45G	9	cooperate in activation of S and G2/M checkpoints following exposure of cells to UV irradiation.	12124778
CDK2	12	cyclin E binds to CDK2 to promote the transition of cells from G1 to S phase ; cyclin A binds to CDK2 to progress the cell cycle through the S phase and G2/M transition.	21319273 ; 18372919; 18372919; 20195506
**cell differentiation**			
FGF2	4	FGF2 was found to up-regulate to FGFR3 until the peak level of FGFR3 mRNA expression, while in hypertrophic chondrocytes, FGFR3 appeared to cause the differentiation of chondrocytes.	15878293
FGFR1	8	FGFR signaling promotes chondrocyte differentiation from condensed mesenchyme.	11585811
GADD45G	9	GADD45G as an instructor of LT-HSC differentiation under the control of differentiation-promoting cytokine receptor signaling.	25068120
MAFB	20	down regulation of Mafb are crucial for osteoclastogenesis.	23132958
**cell division**			
CDK2	12	regulating various events of eukaryotic cell division cycle.	25386824
**cell migration**			
FGF2	4	up-regulatedcten modulates cell migration induced by FGF2.	23625726

FGF10	5	stromal FGF10 induces migration and invasion in pancreatic cancer cells through interaction with FGFR2.	18594526
FGFR1	8	Fgfr1 patterns the pharyngeal region to create a permissive environment for neural crest cell migration.	12514106
NTN1	17	stimulates proliferation, induces migration, and promotes adhesion of endothelial cells and vascular smooth muscle cells with a specific activity comparable to PEGF and PDGF.	15520390
**cell proliferation**			
IRF6	1	a key determinant of the keratinocyte proliferation-differentiation switch ; a developmental feedback regulatory loop between p63 and IRF6 for the control of proliferation of epithelial cells; a primary Notch target in keratinocytes, which contributes to the role of this pathway in differentiation and tumour suppression.	17041603; 20424325; 21909072
MSX1	4	Msx1/Msx2 double-null mutation prevented full activation of Stra8, a gene required for meiosis.	22071108
SPRY1	4	inhibit cell proliferation.	15342396
MMP16	8	distinct and specific functions for MT1-MMP and MT3-MMP in skeletal development.	18470538
ERBB3	12	NRG1-driven/activated-ErbB3 autocrine loop in promoting cell proliferation in human ovarian cancer cells; Proliferation of Colorectal Cancer Cells is Mediated by Either ErbB3/ErbB2 or ErbB3/C-MET Signaling Pathways.	20227043; 24205104
SPRY2	13	inhibiting Spry2 expression in renal cell carcinoma promotes proliferation ; loss of Spry2 was associated with an increase in palate mesenchymal cell proliferation.	21195053; 23688375
NTN1	17	stimulates proliferation, induces migration, and promotes adhesion of endothelial cells and vascular smooth muscle cells with a specific activity comparable to PEGF and PDGF.	15520390
**cellular response to stimulus**			
CREBBP	16	CBP/catenin signaling in enhancing and maintaining the leukemia stem/initiating-like cell population under hypoxic conditions; induce MMP1 expression during prolonged hypoxia and regulates cell migration and invasion in cancer cells.	24720539 ; 23775082
CREBBP	16	induction of Egr1 by UV-C irradiation leads to repression of p300/CBP transcription; p300 and CBP act redundantly in UV lesions.	15225550; 23275565
**metabolic process**			
ABCA4	1	clearance of all–trans–retinal produced by the visual (retinoid) cycle required for vision.	18658157; 19304658

MMP16	8	zinc-bindingendopeptidases that degrade various components of the extracellular matrix.	OMIM
**histone-related**			
CREBBP	16	CBP and its close paralog p300 are transcriptional co-activators with intrinsic histone acetyltransferase activity.	22387215
**phospholipid-related process**			
ABCA4	1	an N-retinylidene-phosphatidylethanolamine and phosphatidylethanolamine importer.	24097981; 22735453; 15471866
FGF2	4	FGF2 binding phosphorylates both extracellular signal-related kinase (ERK) and focal adhesion kinase.	10959077; 15174091
PPAPDC1B	8	a novel type of mammalian phosphatidate phosphatase.	17590538
DDHD2	8	a phospholipase that hydrolyzes sn-1 ester bonds of phospholipids, producing 2-acyl-lysophospholipids and fatty acids.	20932832
**others**			
**eye morphogenesis**			
VAX1	10	critical for the development of the mammalian binocular visual system; Vax1 expressed restricted to the optic stalk.	25201875; 10421837
WNT9B	17	in the ocular surface ectoderm.	16258938
**brain morphogenesis**			
MSX1	4	Driving dopaminergic neuronal differentiation of ventral mesencephalic progenitors.	18385323
PTCH1	9	co-ordinated development of the brain and face is dependent in part upon XIAP mediation of Hh/Ptch1-regulated cell survival and apoptosis during embryogenesis.	25292199
VAX1	10	regulates the guidance properties of a set of anterior midline cells that orchestrate axon trajectories in the developing mammalian forebrain.	10601035
GSC	14	the morphogenetic function of the rostral AML requires Gsc activity.	10751169
MAFB	20	the dual function of Mafb in both segmentation and specification of anteroposterior identity in the hindbrain.	12490204
**lung development**			
FGF10	5	serves as an essential regulator of lung and limb formation.	9988217; 9916808
SPRY2	13	encode negative regulators of FGF10 mediated RAS-regulated ERK1/2 signaling, as essential for controlling airway shape change during development through an effect on mitotic spindle orientation.	21764747

**limb morphogenesis**			
MSX1	4	Msx1 antagonizes the myogenic activity of Pax3 in migrating limb muscle precursors via direct protein-protein interaction.	10529415
FGF10	5	induces formation of the apical ectodermal ridge and is necessary for limb development.	22965740
FGFR1	8	mutations in FGFR1 and FGFR2 with disorders of limb patterning.	11389462
PTCH1	9	Ptch1 and Ptch2 are co-expressed in the developing mouse limb bud; modifications affecting the Ptch1 cis-regulatory landscape have contributed to evolutionary diversification of artiodactyl limbs.	25448692; 24990743
DICER1	14	essential for proper limb bud positioning.	21256124
NOG	17	BMP binding affinity of site-specific variants of noggin was correlated with alterations in bone formation and apoptosis in chick limb development.	12478285
**ear morphogenesis**			
MSX1	4	each mutant mouse displays intriguing facial abnormalities including those of middle ear ossicles.	10512191
FGFR1	8	essential for the normal formation of the organ of Corti and that phenotype severity observed in Fgfr1 mutants is dependent on the dose of FGFR1.	19820032
SPRY2	13	negatively regulates FGF8 signaling during pillar cell formation and for hearing function.	15809037
DICER1	14	Removal of Dicer from sensory epithelium caused abnormal growth and subsequent degeneration of mechanosensory hair cells.	19416898
GSC	14	displays intriguing facial abnormalities including those of middle ear ossicles.	10512191
MAFB	20	loss of hindbrain kr/mafB expression in mutant mice results in an altered inductive signal from the hindbrain to the developing ear that ultimately leads to the inner ear malformations.	16325169
**other organs morphogenesis**			
IRF6	1	plays important cell-autonomous and non-cell-autonomous roles in muscular differentiation and cytoskeletal formation in the tongue; mice deficient for Irf6 have abnormal skin, limb and craniofacial development; TGFβ/SMAD4/IRF6 signaling cascade might be a well-conserved mechanism in regulating multiple organogenesis.	15472655 ; 23451037; 17041601; 23406900
FGF2	4	FGF2 delays tectal neurogenesis and, thereby, triggers a cascade of changes in tectum size and morphology.	24265789

SPRY1	4	positive (GDNF) and inhibitory (Sprouty1) signals have to be finely balanced throughout renal development to prevent hypoplasia or cystic hyperplasia.	17022962
FGF10	5	Fgf10 +/- mice were found to have aplasia of lacrimal glands and hypoplasia of salivary glands.	15654336
GADD45G	9	essential for primary sex determination, male fertility and testis development.	23516551
RAB5B	12	a small GTPase protein that regulates the motility and fusion of early endosomes.	25605758
ERBB3	12	Myocilin mediates myelination in the peripheral nervous system through ErbB2/3 signaling.	23897819 ;
ERBB3	12	NRG-1β/ErbB3/CREB signalling at the pre-mesoderm stage for stem cell cardiac differentiation.	24364879; 12134143
GSC	14	the expression of Gsc in the appendicular skeleton during mid- to late organogenesis, including in the shoulder and hip joint region, and in several ligaments and adductor muscles.	9566956
DICER1	14	Dicer-dependent processes in limiting the timespan of cortical neurogenesis.	24012747
CREBBP	16	CBP/p300 and associated transcriptional co-activators exhibit distinct expression patterns during murine craniofacial and neural tube development.	19598128
WNT9B	17	function during the early phase when the cap mesenchyme is induced to undergo morphogenesis into a nephron; convergent extension of the tubular epithelial cells; balances progenitor cell expansion and differentiation during kidney development.	24445433; 21852398; 21350016
WNT9B	17	a common organizing signal regulating diverse components of the mammalian urogenital system.	16054034
NOG	17	In the developing frog, the noggin protein also mimics the activity of the Spemann organizer, which can make dorsal tissue out of ventral tissue.	OMIM
NOG	17	Nog(-/-) embryos displayed reduction in the dorsal foregut endoderm.	GENE
NOG	17	BMP antagonism is required in both the node and LPM to facilitate L-R axis establishment in the mammalian embryo.	18550712
**axon guidance**			
FGFR1	8	FGFR1 is prominent in the cytoplasm of photoreceptors, and in their axon terminals.	12708794
NTN1	17	attract spinal commissural axons and repel trochlear axons in vitro.	8978605
**angiogenesis**			

FGF10	5	FGFs interact with FGF receptors, which are a family of tyrosine kinase membrane bound receptors with extracellular ligands, and trigger transducing extracellular cues to elicit diverse biological responses such as mitogenesis, angiogenesis, cell proliferation, development, differentiation and cell migration.	25446127
FGFR1	8	angiopoietins as downstream effectors of FGFR1 activation in vivo; FGF2 induces endothelial cell migration and angiogenesis through two classes of receptors: receptor tyrosine kinases, such as FGFR1, and heparan sulfate proteoglycans, such as syndecan 4.	17297442; 22569333
DICER1	14	Dicer1 is essential for normal endothelial angiogenic processes; Dicer1-dependent miRNA expression may be a potential mechanism underlying impaired angiogenesis and cerebromicrovascular rarefaction in aging.	23239824

Data was derived from the database of Pubmed (http://www.ncbi.nlm.nih.gov/pubmed) and summarized from the related literature.

Supplementary Table 15. Primer sequences.

SNP_ID	2nd-PCRP	1st-PCRP	UEP_SEQ
rs7554547	ACGTTGGATGTGAGGGCGAATAGATGTTGG	ACGTTGGATGCCTCATCTGTAAAACAGGAC	gagGACCTCCAGTATTCCTGCAAGG
rs9439713	ACGTTGGATGCCCTCTCTCTTCAAATTAGG	ACGTTGGATGTCTCTGTGTCCCCTGTATGG	TCAAATTAGGTTCTGGTTGG
rs742071	ACGTTGGATGTTGCAATCCTGGTTAAGGAC	ACGTTGGATGTTGATCCACTGTAACCGTGC	gggTGTTGTTGCAACGGT
rs11582254	ACGTTGGATGAGTGTGAGCCACACTGTGGT	ACGTTGGATGAGCTGCCTGTTCTGATCACT	ggtTGAGGCCTAGCTTCAT
rs1883567	ACGTTGGATGGGGCTCTGGAGGTTCAAATC	ACGTTGGATGCAACCCTGTCTCTAAAAAGC	tcGGTTCAAATCCAATCCTG
rs6588607	ACGTTGGATGCTGAGTTCTTCCCAATAAGC	ACGTTGGATGGACTCATGCCTGTGATGTAG	CCAATAAGCTACATTGAATTTTTT
rs1331862	ACGTTGGATGCCATTCATTAACCTGTTCTC	ACGTTGGATGTAGGAGATCAGGTGCCATAC	gtcACCTGTTCTCAGGTTAGTAAGT
rs277354	ACGTTGGATGGCTGTTAATAATTCCCAGAG	ACGTTGGATGTAGACCACCAAAACTTGCTG	gCCCAGAGAGAATAATCTGC
rs7367789	ACGTTGGATGTTACTCAAGGCCATATCCCC	ACGTTGGATGGTTGTGTAAGTGTGATTGGC	CCTGAAATACTACAGCCA
rs4147827	ACGTTGGATGCTGTAGCAGGTGAGCATTAG	ACGTTGGATGTCTCAACCTGGGCCGAGTAT	ggGAGCATTAGTACAATGGGCT
rs17461953	ACGTTGGATGAATCTACTTGGGAGGAAGC	ACGTTGGATGGGCAAACTTCTACTCATCCC	GGAAGCTGTTTCTCACA
rs481931	ACGTTGGATGTGCTGTTCAGGGAAAGGCAC	ACGTTGGATGTCTCCAAATTCTCCCCTCCC	tTCCAGCACACAGGGG
rs4147803	ACGTTGGATGCATCCCTGTCCTTGACGCTT	ACGTTGGATGGTCTGAGCTGCTGAGAAAAC	TCCTTGACGCTTAACTGC
rs12047887	ACGTTGGATGCCCTTGCCACTTGGATCTTA	ACGTTGGATGTCAAAGCAGGGGATCCATTC	cGTCACTTTCACAAACCTTT
rs4839542	ACGTTGGATGCTGCTTGCCATTCACATGAC	ACGTTGGATGAGAGCAAGCATTGGGCATTA	TGTTTTTCCTCTCTGAGT
rs10918593	ACGTTGGATGATGTTGGAGATGGATGTTAC	ACGTTGGATGCACACAACCATGGCACATAG	cGATGGATGTTACTTCGTTC
rs861020	ACGTTGGATGTGAAAACCCAAATGGCCAAG	ACGTTGGATGCACCTGTTGTTAATGGCTAC	aaacTGGCCAAGACAGAAAC
rs642961	ACGTTGGATGTCTTACCCAAAGGCCTGAAG	ACGTTGGATGGTCAAGGATAGAGCATGCTG	tgatGCCTGAAGTAATACCCCAG
rs2064163	ACGTTGGATGGGCCTGGTGGTTAAGAAAAC	ACGTTGGATGCAACCATGCATTAGTCCTCC	atgtTGGTTAAGAAAACAAAATGAATTT
rs9430019	ACGTTGGATGCTCTGAAACAGCAGCAGTTA	ACGTTGGATGTTTCTTTTGCAGTGAGAGCC	cATAAATTCTTTTTTGCTACAGTTT
rs11583395	ACGTTGGATGGGGATTAAAATGGCTGAGGG	ACGTTGGATGCGTTCTTTACAAATAACCTC	ccgcGGGCATTTAAAAAAATTTATGCT
rs201830371	ACGTTGGATGGTTCTTAAAGCTTTGACACTC	ACGTTGGATGACTAGGAATCCCTAACCGTG	ctcgTGAATTTCCATCTGCACA
rs287980	ACGTTGGATGTGACTGATGATCTTGACGTG	ACGTTGGATGGAGCAACTCTTGGCTCATTG	aTGATGATCTTGACGTGATATAC

rs287982	ACGTTGGATGACTTGCTTCATGTGGCTTCC	ACGTTGGATGGCATTAGATGTCAGGGACAG	ccCTCCTCTGGTTCTCTTC
rs10172734	ACGTTGGATGGTGTTTAAGCTTTCTGAGTC	ACGTTGGATGTCGGCATTAACCTTACAGGG	GCTTTCTGAGTCTTAACCTC
rs7552	ACGTTGGATGCTTTGCTACTGGAGTCCTTT	ACGTTGGATGTCCCAGAGAGATAACACATC	aTACTGGAGTCCTTTAACAACA
rs7590255	ACGTTGGATGGTTCTTTATCTTTGTCCTAC	ACGTTGGATGCAATCTGAAAGAACCATCCC	gtagTTTCTCTCTCATTTCAAAGCA
rs17491637	ACGTTGGATGTTAATACAGTTTTAGATCAG	ACGTTGGATGGTGGGTCCATCAACAGAAAC	aagACAGTTTTAGATCAGATGATT
rs11677862	ACGTTGGATGAAAGATGTTTTATGATGCC	ACGTTGGATGGCAACTGTTTTTCCTAACTC	ATGTTTTATGATGCCTGTATG
rs12623132	ACGTTGGATGCAAAACATGCTGCTTTAGAG	ACGTTGGATGATTCTAAAATCAACCTACC	agTGAATACTTGGTTAGAGATGA
rs6734545	ACGTTGGATGAGAAGACTGGCTGGGAAAAG	ACGTTGGATGGGAGACATGAGGAGGAAATC	GGCTGGGAAAAGTTGATGAG
rs77866552	ACGTTGGATGGCCAGTTACTGAATGTGGTC	ACGTTGGATGAGAGTCAGCAAGGAGAAATC	CAGCTGTGGTTTTGTC
rs78991648	ACGTTGGATGGCTGGCTTTAATATTTCCTG	ACGTTGGATGTGGGTCCTAATTACAGTCAC	ctaCTGTTTTTAAGACCTTTTTTCT
rs1395165	ACGTTGGATGGGTTTTGTTCTTTAACCGCC	ACGTTGGATGACAGGCAGTCTCTAGACCTC	tcACCGCCTTCCAGCAT
rs12485312	ACGTTGGATGCTCACACTATGGGAGTAGAC	ACGTTGGATGCAACCATGCTCATCTGATAG	ATGGGAGTAGACAGATAAGTGA
rs1038294	ACGTTGGATGGAGTTACTTACCTGAGGGTC	ACGTTGGATGAGGATATGTACCTTTGTTGC	GTCATAGATCAATATAGTTAGAGTC
rs1688766	ACGTTGGATGATAAACTCTGGCAACCTACC	ACGTTGGATGGGCAAGACAGTAGTCTCTTC	aacgCTGGCAACCTACCTCAGGA
rs9847858	ACGTTGGATGGAAGGTTGGCAAGTTGCATC	ACGTTGGATGCTGCCAAAGAGGAAATCGAC	AACAGCTGCTCTTGT
rs10936656	ACGTTGGATGAGCAGAAAGGTATGAGCAGG	ACGTTGGATGCTTTTCCTCCAATGTTCCCC	acgATAGTACATGGCATGAGTG
rs17597256	ACGTTGGATGACCAGGGAAAAACATGGGTG	ACGTTGGATGGGAGGGAGATAAATGGAATC	cccctTGTCACTTTCCCGACC
rs7649443	ACGTTGGATGGGTATATGACATTTCCTTCC	ACGTTGGATGAAACCACTGACAGCAATCGC	GTTCTTCTTGGCCCTTA
rs34246903	ACGTTGGATGTTCCACACTGTTTCTGTGAG	ACGTTGGATGGACCTATGGAGTTCTCAATG	ggggtTGAGAATTCATTAAGGATCAC
rs1907989	ACGTTGGATGTTTGAGGCTTCCTCTGATCC	ACGTTGGATGCAGTAGAAAGGGTTCCCTTG	TTCTCAAAGAAACATCACTG
rs7692299	ACGTTGGATGAAAGGTGCTCAATACATGGG	ACGTTGGATGCAAAACACATTCTAGTCCAGC	GGATGATGGAAAAATATTTTATTATGA
rs908822	ACGTTGGATGCAGAAATGCTCCAACAGCAG	ACGTTGGATGCTGCTGCAGGGAAGCAGGG	GGCTATCAAAGCCCC
rs7686589	ACGTTGGATGCTTCTATCAGAGTTGGTATC	ACGTTGGATGCAGTTTAGCCATGGTTTCTC	aagcGTTGGTATCTTTAAACTTGACC
rs66509882	ACGTTGGATGGTAGACCATCAAAAGAGCAG	ACGTTGGATGCCTCCCAAAGTGCTGGAGT	tgATGAGGAATTTCTAAAAGACAGATC
rs4417922	ACGTTGGATGAGGTTATGGTCCACAGACAG	ACGTTGGATGTCACAGCTTCTAGTCAGCAG	ccggCCACAGACAGTCATTTATG
rs2333571	ACGTTGGATGCATGGGTACTCTGGACAAAG	ACGTTGGATGGGACATCTTTGAATAGTGTG	TGGAGAGGGACAGTG

rs13124707	ACGTTGGATGCTGGCTGTCAATCTTGCAAT	ACGTTGGATGGAGTATAGTATGATCTATG	aaTATCTCCCTCTCTCTCTTC
rs10462065	ACGTTGGATGGTGTGGTTCTAAAGCTGCTG	ACGTTGGATGGGTCTATAGGAGTGTTGTAG	GCTGGCAATTTAGGTG
rs4242107	ACGTTGGATGTTGGGTCTTACAGTCTAGAG	ACGTTGGATGTCTTACTACTCCCTGTCTTC	gTTACAGTCTAGAGAGAATAGAG
rs10462084	ACGTTGGATGTCTGCTGCCTTATTCAAGCC	ACGTTGGATGAAAGGTAGAAGCAGACAGCC	cttagTCAAGCCAGTACACTC
rs7771515	ACGTTGGATGAAAGGCACTGGGCTCTGAAG	ACGTTGGATGTTCCCCTTCACTACTCATTC	tcctTGGGCTCTGAAGAAAAGAGG
rs9381107	ACGTTGGATGACTTACTACCTGCCCTGTTG	ACGTTGGATGCATCCACTGGGATCTTTAAG	gaggaCCTGTTGCTCAAAAAAGAG
rs11965246	ACGTTGGATGGGTTGACTCATAAGTGATGG	ACGTTGGATGTTCAGGTAGTGGGCACAATG	aataACACTTTTGCTACCAAAAGAAA
rs12203226	ACGTTGGATGCTCCCCTACTCCCAAAATTC	ACGTTGGATGCTTCTTAAAGACCCTAGCTC	agcaCTAACTCCCAGTATCTCC
rs10260512	ACGTTGGATGCCCTTCTAAAGCTCAGATCC	ACGTTGGATGGGAGATGGACTTCAGGAAAC	tcTTTGAGATTTCAGGAAATAGT
rs76523544	ACGTTGGATGGGAAGAAAATGTTTGTGCCC	ACGTTGGATGGAAAGTGGTCAGAGTGTGTC	TGCCCTCTAATTTAATTCAA
rs10256809	ACGTTGGATGCTGAAGAGCTATGCAGTGGG	ACGTTGGATGGCTATGACCATTGTGCTGAG	CTCAGAGATGCTCCAG
rs17227506	ACGTTGGATGTCAGACAGGGCCCTATACAC	ACGTTGGATGATCAGATTTCTGACTAGCTC	gatagGTTTACTGGAAGACTGGAATTT
rs13317	ACGTTGGATGCAAAACTAGCCTCAGGTGAC	ACGTTGGATGAAACCCAGACATGTGTCTGC	CCTGCACTTAGAAGCAC
rs3925	ACGTTGGATGCAAGCGTTTCCCAGTCTTAG	ACGTTGGATGGATGCTCTTATCATGCACCC	ccgtgTCCCAGTCTTAGCCCTGTGT
rs12543318	ACGTTGGATGGTTTCTTGTTACAAATGGC	ACGTTGGATGCAGAATAGCCTGTTTTCCCC	gagTGTTACAAATGGCTTTCAT
rs1034832	ACGTTGGATGCTACAGTTCCTCAATAGGAC	ACGTTGGATGTCATGGACACAGTTTTTAC	ctgTTCTGGCACTATTATTCAAA
rs12681366	ACGTTGGATGCAGACACCTTCTGTTAGTGG	ACGTTGGATGGACCAATCAGTCTATGCACG	aaTGGGGACGTTCTTTCAT
rs957448	ACGTTGGATGAGTCCTAGCTAACATAACCC	ACGTTGGATGTAGACAGTATTCCTGAGGAG	gAGCTAACATAACCCACCTTC
rs11774066	ACGTTGGATGTAGTCACATCTCTGGAGTTC	ACGTTGGATGAGATGTGAGGCATCTGTCTG	gAGAAAGCATGTCTAGACC
rs1487022	ACGTTGGATGAGCTGGAAGTCAAAGACTTG	ACGTTGGATGGTCTGCTTTTCAACCTGGAT	CTTGTAGATTTAGAATCCTGATAGTAA
rs1993392	ACGTTGGATGACAGAATTGTGAAACCACCC	ACGTTGGATGGCAGCCTTTTCACAACTTGG	ACTTTGAAATAGGGGCA
rs7845615	ACGTTGGATGTTCATTTCCACCTTGCACGC	ACGTTGGATGAAGGCACCCTGATTTGGTAG	GAATTACCTTGTTTTGAATGG
rs7017252	ACGTTGGATGTGGGAACCTCAACACCTATG	ACGTTGGATGGAACCCAGATCTTTCTGAC	ccttgGCAAGGCCTTTAATATCTCG
rs327753	ACGTTGGATGAGGAAACCCCTCTCATCTAC	ACGTTGGATGGCAGCAAAGATACTGAATGG	CTCTCATCTACTCTCCACT
rs2990600	ACGTTGGATGGAGAAGATCAGACTCATCCC	ACGTTGGATGGAAGTATACTCCAAAATCTG	GATGTCTGGGTTAGCAC
rs408960	ACGTTGGATGCTGGAAGAGGAGCAGTATAA	ACGTTGGATGGCAATAGATGATCCTCAAGC	GGAGCAGTATAAAAATGAGTT

rs324475	ACGTTGGATGCCCAATCAGGGTGCATTAAG	ACGTTGGATGGATGTGAAGAGCCAGTTTTC	tttatTCACAAATCTTTGCATGACACT
rs77401103	ACGTTGGATGTGCTTTCCATATTTTGACC	ACGTTGGATGGGGTCTATTAGTTAAAAGTG	ggggCCATATTTTGACCCAGGAA
rs542733	ACGTTGGATGACCAAAGCTGACTCTTTGGG	ACGTTGGATGGTTCACAAAATTTTAAGGTC	AGGTTACAACCCTGAAG
rs498010	ACGTTGGATGATCCAGCACACTGCGCATAG	ACGTTGGATGACTACCAACCATTGACTGGC	CTCTCGAGTCGGAGT
rs35825512	ACGTTGGATGATACCATTCCCCAGTAAAGG	ACGTTGGATGGTCTCACCTTATACCTTGCC	CCCAGTAAAGGATCAGTGT
rs7863754	ACGTTGGATGTGCTGGCAAGAATGTAGAGG	ACGTTGGATGTTCTCCACAGTGGCTGCATC	aagcATGTAGAGGAATTGAAGCCCT
rs7871395	ACGTTGGATGACCTCCTTCCCTGCTAGAAT	ACGTTGGATGCCAGGCAGTCTCTAAATAAG	gaTTCCCTGCTAGAATTCATTC
rs10512248	ACGTTGGATGGCAGGAGAAAACTGGAAAGC	ACGTTGGATGAGCCAGTCTGGTGTTCTGTG	AAACTGGAAAGCCATTTAC
rs28361526	ACGTTGGATGTGAACACTACGTCTTAGTCC	ACGTTGGATGCTACCAGAAGCTGGGAATCG	AATTGTATGTTTGTATCCGT
rs13294988	ACGTTGGATGCATCCCACTTTACTCTCAGC	ACGTTGGATGAAGAGGAAAGGTTGTGGAGG	AGGTAGTGAAAAGTACTGTTT
rs474558	ACGTTGGATGCCGTCATCCATTCCTTAGTG	ACGTTGGATGCCAGGCTATGATGATGATGC	ggaaTTGTTACTCAAAGTGTGGT
rs6585429	ACGTTGGATGTCTAGAGCAAACGTCCTGTG	ACGTTGGATGAATGCCCGAGTTAGTCAACC	atGCTGGGCACGGGGTC
rs12792211	ACGTTGGATGGCCCATGTTATTCCCTCTAC	ACGTTGGATGCTCTCTTCCTGTCTTGATG	ccTCTCTGGGCCAGCTC
rs3741442	ACGTTGGATGTGTGCACAAATGCCCTAACC	ACGTTGGATGCTTCCTTCTACACCCCAATC	caccAATGCCCTAACCCAACAGTC
rs705704	ACGTTGGATGCAAGTGCTTAGACCGAAGGC	ACGTTGGATGCCTCTTCGTAATCCTACACC	TTCTCTGACAGTCCTTCTA
rs2304269	ACGTTGGATGTTTTGCGGTGAGGCGTTGAC	ACGTTGGATGCAAACTCCTTCCGGAAACTC	cccttACCACGCCCATATGAA
rs7967428	ACGTTGGATGCAGCCACCTTAAAATACCAC	ACGTTGGATGCCATTCCCTCAAAGCTTGTC	tATGAAGCAATTAATGTTCATG
rs2188380	ACGTTGGATGCCACATTCTTCCCCAGCATT	ACGTTGGATGTAAGACCCCCAGTTTGTGGC	gGCATTTGGAATGGTCAATA
rs12229654	ACGTTGGATGTTATGCATAGGGTACCAGGC	ACGTTGGATGGTTTTGGAGAAAGGTACTTGC	GTTTGAAATAATACAAATAATTTCCATA
rs4766453	ACGTTGGATGCTATGTATTTAGGCACTGGG	ACGTTGGATGTCTGCAACAGCAGCAGATGG	gggaGAGGGCTGGCTTGATTC
rs11066150	ACGTTGGATGGCATTCAGAATGGGATTGAG	ACGTTGGATGAAAAACCAGTTCAGAAGAC	TGGTTTTATTTTTTATATAGTTATTGTC
rs12229892	ACGTTGGATGGATGACTAGATTTAGGCTCC	ACGTTGGATGGAGGTTTAGAGATACCCAAG	cctaCAATTCCAGGCACTCACACTCA
rs9545308	ACGTTGGATGCAAAAGGTGATCCAGGTCTC	ACGTTGGATGGGAGCTTCCAAGGTCAGTAA	aggttATCCAGGTCTCTTAGAATAA
rs1168980	ACGTTGGATGATTGAGTCCTGTGCCACAAC	ACGTTGGATGCAATGCTGCATATGTGTACC	aacgCAACTCACACACGCA
rs17103683	ACGTTGGATGAGGAACACGTAGGTCAGAAG	ACGTTGGATGGTGTTCCCTTTTCAGCTCAG	ccacaAAGTGGACAAAAGCAGACA
rs1956424	ACGTTGGATGGTTATGCTTCTTATTTCCC	ACGTTGGATGAACAGCTTGTCAGGGTGAAC	ctGTCACTCCCCTGTCC

rs10133673	ACGTTGGATGCAGAGTCAGGGTTCATAGTC	ACGTTGGATGTCCTTCGGAGCTCCTTATTC	ggccGGGTTCATAGTCACCACAT
rs7148069	ACGTTGGATGTACTGCTATTGCAATCACCC	ACGTTGGATGGATGCCAAACTTTTCTTAGAG	cAATTTTGCCCTCCACC
rs10134081	ACGTTGGATGCTTCCCTTTCATCACTGGAC	ACGTTGGATGAAAGGCAAGTGCTAATGCCC	CCTCCCTGTTGCTCC
rs9671386	ACGTTGGATGGTGCTGGCCTGGAATGTTAA	ACGTTGGATGGTGACTCTTCAGATAGTAGC	tctgTTCTCTTGTTTTTCAACTCC
rs1243572	ACGTTGGATGTTCCCTCAGTAAGCAGCTTG	ACGTTGGATGTTCCAATTGGTCTGTTTCCC	cttcgTTGTGCAAAGCTCACTGCATTA
rs1243573	ACGTTGGATGAGACAAGCTGCTTACTGAGG	ACGTTGGATGTCTTCTTTCCCACTGCTTAG	GGGAAGGGCTAGGAC
rs78512626	ACGTTGGATGAACTGCTGTAAGGTCAGCTC	ACGTTGGATGCAAGGAATAGCAATGTGGAG	CTGCTATGAGGTTTTTTTTTC
rs2289187	ACGTTGGATGTAATACAACCTGCCCGTATG	ACGTTGGATGGCCAAAGTCGAAGAATGAGG	gggGCCCGTATGGTGACA
rs6495117	ACGTTGGATGTCTCCTGGGCAAGTGTATGT	ACGTTGGATGGCAGATGTCTGATGTGTATC	tCAAGTGTATGTGTGCATGG
rs2283487	ACGTTGGATGACTTTGCTCTGGAAGAAGGG	ACGTTGGATGAAGCTTTGGAGGTGCTCCAG	GGTTTTCCAGATGGCTC
rs17136624	ACGTTGGATGTGCTCAAGCACATTCCTTCG	ACGTTGGATGGTTCTCTGAAAAAGGCGCTG	ctATTCCTTCGTGTTCAAGTATA
rs4786640	ACGTTGGATGGTCTCTCCCTTGGAATAAAG	ACGTTGGATGTTCATCTACGTCTCCATGGG	ATGTCTGAGTTTTCTTCCTCAA
rs7198766	ACGTTGGATGTCCTGTCTGGGCCTCCTAAA	ACGTTGGATGGACAGCACATATGTGTGCTA	ggtcTGCCCTGCCCAGACC
rs7188937	ACGTTGGATGCTTTTTAAGTGCCACCGACC	ACGTTGGATGACAGGTTGTGGAAGATCCTC	gCATGTCCAATGTATCCTCTTC
rs217181	ACGTTGGATGGGGTTGAGCACATTGTCAG	ACGTTGGATGGCTTACAGATTCAAGAATCC	gggttAGCACATTGTCAGTGGTTA
rs2872615	ACGTTGGATGTTCTCATGGCTGCTGGTATC	ACGTTGGATGAGGATAAAGCTGCGTGAGAC	gcgGCATGGCTCCAATCTC
rs1880646	ACGTTGGATGGCATGGGTACTTCTTTACAC	ACGTTGGATGCTTCCGCCCATATGTATCAG	GAATGTGAATGCACATGTGTA
rs4968247	ACGTTGGATGCCACATCTTAATGGTCCAGG	ACGTTGGATGCTCAATGTACTTCACCCCTC	ttGGGTCCCAAAAGGAGA
rs1838105	ACGTTGGATGGCATTTCATTATGGGTGGAC	ACGTTGGATGTTCTGCTCTTTGGTTCCTGC	gaTGGACTGATTGAAGTGA
rs12951993	ACGTTGGATGTTGCCAGATGAGCATGATCC	ACGTTGGATGCTGCACAAGGCAAACTTGAC	ATGTCCAATTCTCTGAAATC
rs227731	ACGTTGGATGACCTTGAGTATGGTGATGAC	ACGTTGGATGGAAAGCCAGCTTATCTGTTAC	gggGATGACTTTTTAGATACCACA
rs3903236	ACGTTGGATGCACAGAAGATGCAGTAAAACC	ACGTTGGATGTCTCCCTACATGAAATATGG	AGAAGATGCAGTAAAACCATTAAATA
rs79280643	ACGTTGGATGAGCTGCTACTATGAAGTTGG	ACGTTGGATGTATTTGCTCTTTGGGGCCTC	GAACTTGATATTGTGACTGTA
rs117386717	ACGTTGGATGCTCCATTTCTCTTTGATTCG	ACGTTGGATGGTAACTGTCACATCCACTAC	AAATGGTTTTATTCAAAAAGGA
rs739461	ACGTTGGATGGATCACACGAGATATTCAGC	ACGTTGGATGGATGAGCTATAATCAGAGTC	AAACATTTTCTTTCCATAGAAG
rs6129653	ACGTTGGATGCAGCTATGGTCAGAGGGAG	ACGTTGGATGTGCAGGGTAGAGTGTGCGT	ccccCTATGGTCAGAGGGAGTGGCTA

rs6126344	ACGTTGGATGACTAATCCCAAGGACCTCAC	ACGTTGGATGTCACTTTCTGGAGAAGGCCC	CCTTGCCCGGTGACC
rs2018054	ACGTTGGATGGTCTACTTTCTATGCAGACAC	ACGTTGGATGTCCCAGCAACTTGGCTTTCC	ggttATCTCTCTTTCTTTTCCCCAC
rs933150	ACGTTGGATGACTGGGTCTGTGCACAGTAG	ACGTTGGATGATGAGCATTGATAACGGCCC	cccgtTCTGTGCACAGTAGACCTACTA
rs7287092	ACGTTGGATGTGGAAACCTTGAACTGAGGG	ACGTTGGATGAGTCATCCTTCGGTTCTCAG	ACTGAGGGGAGGGTC
rs2073764	ACGTTGGATGTTCACCAGGGACTTGGTCTC	ACGTTGGATGGAGGACCTTAGACCCAAGTG	CCTCCTGCCAGCTGC
rs8135014	ACGTTGGATGATGTGCCATGTCCTACTGTC	ACGTTGGATGTGACTTGGTCGGGTACATGC	TACCTCCCAGCCACC
rs36604	ACGTTGGATGAATGTCTCAAAAGGAACCAC	ACGTTGGATGTCCGTGTGGACAACTTAAGG	GGGCTTTAAGACCCC
rs5763674	ACGTTGGATGCTCATCCCAATCATGGTGGC	ACGTTGGATGTACTGCATTCCTCTGACCTC	gggCATGGTGGCTCATCA
rs11089442	ACGTTGGATGTCGCTGTTGCAGAAGGTGTC	ACGTTGGATGAAGGCGCACATGAACCATTC	ccGATCGACCCGCTCCT
rs2839998	ACGTTGGATGCTCCAACACCCACAGCATTG	ACGTTGGATGCCTGGTTTCTGAGTAAAGGC	ctTCTCCCTGTGATAGCTT
rs2072196	ACGTTGGATGAGAGGAAATTCCAAGACCCC	ACGTTGGATGGGATGACTGAGCAGAAGAAC	cttTCGCCTCCAATGTAAA
rs117353193	ACGTTGGATGCTAGCTACTACCAGTATGGC	ACGTTGGATGAGTTTGTCCACCACGCTGTC	GTCTCCACCAGAAGC
rs5753388	ACGTTGGATGCAATTACAAACTAATGTCAC	ACGTTGGATGACTAACCCTGCATTTTTGG	ACAAACTAATGTCACTTGTAA
rs1003480	ACGTTGGATGGGCAGAAGTGATAGTAATCC	ACGTTGGATGCCCACTACCACTGAATATGC	GATAGTAATCCAGGTGGAG
rs2073856	ACGTTGGATGCACCTATGCTATCACTACCC	ACGTTGGATGCACCCAGCATGGTCCTTTG	TTAAAGGGCAGAGGGG
rs17220418	ACGTTGGATGTACAGATTGAGAGCTTCTGC	ACGTTGGATGCCAGCCACAATCACTCTATG	GCTTCTGCTATTATTCCATTA
rs2006771	ACGTTGGATGTTCTCTCCTTCCTGTCCTGC	ACGTTGGATGGGTGCCACATGAACCAAGAC	TCCTTCACGCAGAGC
rs78212183	ACGTTGGATGCTTGGCCATCATTATCTACC	ACGTTGGATGTCCTGCAAAGCAACTTGAGG	gaccTCATTATCTACCCAATGCCT
rs5756130	ACGTTGGATGCTTCCGCAGCTGTTTGATGG	ACGTTGGATGAGCTGGAGATGGACCTGAAG	cctcaGCAGCTGTTTGATGGCTTCGTC
